# Mechanistic basis of breast cancer resistance protein inhibition by new indeno[1,2-*b*]indoles

**DOI:** 10.1038/s41598-020-79892-w

**Published:** 2021-01-19

**Authors:** Diogo Henrique Kita, Nathalie Guragossian, Ingrid Fatima Zattoni, Vivian Rotuno Moure, Fabiane Gomes de Moraes Rego, Sabrina Lusvarghi, Thomas Moulenat, Billel Belhani, Geraldo Picheth, Sofiane Bouacida, Zouhair Bouaziz, Christelle Marminon, Malika Berredjem, Joachim Jose, Marcos Brown Gonçalves, Suresh V. Ambudkar, Glaucio Valdameri, Marc Le Borgne

**Affiliations:** 1 Pharmaceutical Sciences Graduate Program, Laboratory of Cancer Drug Resistance, Federal University of Parana, Curitiba, PR 80210-170 Brazil; 2 Laboratory of Cell Biology, Center for Cancer Research, National Cancer Institute, National Institutes of Health, Bethesda, MD USA; 3 EA 4446 Bioactive Molecules and Medicinal Chemistry, Faculté de Pharmacie - ISPB, SFR Santé Lyon-Est CNRS UMS3453 - INSERM US7, Université Claude Bernard Lyon 1, Univ Lyon, 69373 Lyon, France; 4 Department of Clinical Analysis, Federal University of Parana, Curitiba, PR 80210-170 Brazil; 5 Laboratory of Applied Organic Chemistry, Synthesis of Biomolecules and Molecular Modelling Group, Badji-Mokhtar-Annaba University, Box 12, 23000 Annaba, Algeria; 6 Département Sciences de la Matière, Faculté des Sciences exactes et Sciences de la nature et de la vie, Université Larbi Ben M’hidi, Oum El Bouaghi, Algeria; 7 Research Unit for Chemistry of the Environment and Molecular Structural, University of Constantine 1, Constantine, Algeria; 8 Small Molecules for Biological Targets Team, Centre de recherche en cancérologie de Lyon, Centre Léon Bérard, CNRS 5286, INSERM 1052, Université Claude Bernard Lyon 1, Univ Lyon, 69373 Lyon, France; 9 Institut für Pharmazeutische und Medizinische Chemie, PharmaCampus, Westfälische Wilhelms-Universität Münster, Corrensstr. 48, 48149 Münster, Germany; 10 Department of Physics, Federal Technological University of Paraná, Curitiba, PR 80230-901 Brazil

**Keywords:** Medicinal chemistry, Biochemistry, Cancer

## Abstract

The ATP-binding cassette transporter ABCG2 mediates the efflux of several chemotherapeutic drugs, contributing to the development of multidrug resistance (MDR) in many cancers. The most promising strategy to overcome ABCG2-mediated MDR is the use of specific inhibitors. Despite many efforts, the identification of new potent and specific ABCG2 inhibitors remains urgent. In this study, a structural optimization of indeno[1,2-*b*]indole was performed and a new generation of 18 compounds was synthesized and tested as ABCG2 inhibitors. Most compounds showed ABCG2 inhibition with IC_50_ values below 0.5 µM. The ratio between cytotoxicity (IG_50_) and ABCG2 inhibition potency (IC_50_) was used to identify the best inhibitors. In addition, it was observed that some indeno[1,2-*b*]indole derivatives produced complete inhibition, while others only partially inhibited the transport function of ABCG2. All indeno[1,2-*b*]indole derivatives are not transported by ABCG2, and even the partial inhibitors are able to fully chemosensitize cancer cells overexpressing ABCG2. The high affinity of these indeno[1,2-*b*]indole derivatives was confirmed by the strong stimulatory effect on ABCG2 ATPase activity. These compounds did not affect the binding of conformation-sensitive antibody 5D3 binding, but stabilized the protein structure, as revealed by the thermostabilization assay. Finally, a docking study showed the indeno[1,2-*b*]indole derivatives share the same binding site as the substrate estrone-3-sulfate.

## Introduction

Multidrug resistance (MDR) has been described as a major challenge in cancer therapy^1^. Although there are several known mechanisms of MDR, the overexpression of ATP-binding cassette (ABC) transporters is considered the leading cause for the development of drug resistance in many cancers^2^. These overexpressed ABC transporters increase the efflux of chemotherapeutic drugs, decreasing intracellular accumulation to subclinical concentrations^3^. ABC proteins are described as polyspecific due to their ability to export a wide range of drugs with unrelated chemical structures and cellular targets^4^. Actually the human genome codes for 48 ABC proteins^5^. However, the precise number of members contributing to clinical MDR is still under investigation. Three extensively studied ABC transporters have been implicated in the development of MDR: P-glycoprotein (P-gp/ABCB1), multidrug resistance associated protein 1 (MRP1/ABCC1), and breast cancer resistance protein (BCRP/ABCG2)^2, 6^.

A common strategy to overcome MDR is the use of inhibitors. Potent small molecule inhibitors have become a source of potential drug leads^7^. Despite the in vitro success of P-gp inhibitors, preclinical and clinical studies failed to improve the chemotherapeutic efficacy^8^. There are various reasons for failure in the clinic, including the fact that many drugs transported by P-gp are also transported by ABCG2 and other ABC transporters^9^. Since P-gp and ABCG2 are both overexpressed in several cancers, and despite recent advances, there are very few potent, non-toxic ABCG2 inhibitors available for clinical studies, demonstrating that the development of new ABCG2 inhibitors is an urgent necessity^2, 10^.

The first inhibitor of ABCG2 described was the fungal toxin fumitremorgin C (FTC)^11^. Although its selectivity toward ABCG2 is very good, FTC is neurotropic and triggers abnormal excitation of the central nervous system^12, 13^. Because such neurotoxicity precludes its clinical use, FTC analogues have been synthesized and tested as ABCG2 inhibitors, with the most promising candidate being Ko143. To date, this compound is considered as a reference inhibitor of ABCG2 for in vitro assays. However, the effect of Ko143 is not specific to ABCG2, because at higher concentrations, it also inhibits P-gp- and MRP1-mediated drug transport^13, 14^.

During the last few years we have identified certain classes of compounds as potent ABCG2 inhibitors, including chromones^15, 16^, stilbenes^17^, chalcones^18^ and indeno[1,2-*b*]indoles^19, 20^. Designed initially as casein kinase II (CK2) inhibitors, the indeno[1,2-*b*]indole derivatives seem to be the most promising. Previously, we showed that indeno[1,2-*b*]indole derivatives that inhibit CK2 can be successfully converted into potent ABCG2 inhibitors^19, 20^. In this study, further structural insights on rings A, B and D led us to synthesize new indeno[1,2-*b*]indole derivatives. In addition, the mechanism of ABCG2 inhibition and the differences between compounds that produce a complete versus partial inhibition of mitoxantrone transport were also studied.

## Results

### Chemistry

Tetrahydroindeno[1,2-*b*]indole-9,10-diones **5a–5n** and 9-hydroxyindeno[1,2-*b*]indol-10-ones **6a**, **6c–6e** were synthesized on the basis of previous reported methods^21, 22^ as shown in Supplementary Fig. S1.

The synthesis began with the preparation of enaminones **2** by reacting primary amines on commercially available cyclohexane-1,3-diones **1a** and **1b** or cyclohexane-1,3-dione **1c**^23–25^ (Supplementary Fig. S2). The latter was prepared by a consecutive Michael-Claisen cyclization^26^. Then, condensation of **2** with ninhydrins^27^ **3a**, **3b**, **3c** or **3d** (Supplementary Fig. S2) led to the 4b,9b-dihydroxylated derivatives **4**. It should be noted that the reaction with 4-hydroxyninhydrin **3b** led to a mixture of 1-OH and 4-OH regioisomers not separable under classical conditions. The reaction with **3c** led only to the 3-OH regioisomer, while the also expected 2-OH regioisomer was not obtained. Compounds **4** were then deoxygenated using tetraethylthionylamide (TETA) to obtain target compounds **5a–5n**.

Generally, at this step, the regioisomers were separated by a chromatography column. Their structures were determined by NOESY experiments, as indicated in the supporting information and summarized in Supplementary Fig. S3.

For **5f**, the assigned regiochemistry was confirmed by X-ray crystallography. ORTEP representation of **5f** is shown in Supplementary Fig. S4. Structural resolution revealed that the crystal structure of **5f** crystallizes in the monoclinic space group P 2_1_/n with 4 formula units in the cell and confirmed results of RMN analysis. The structure was refined with a disorder in the cyclohex-2-enone moiety at a ratio of 35:65.

The *O*-prenylated derivatives **5j–5n** (Supplementary Fig. S5) were obtained from their hydroxylated homologues by Williamson reaction with 3,3-dimethylallylbromide in the presence of NaOH at room temperature. The confirmation of their structures was also based on NOESY correlations and discussed in the supporting information.

In the last step, aromatization of the ketonic compounds **5** into the phenolic compounds **6**, was carried out with 10% Pd–C at refluxing diphenyl ether^28^.

**Figure 1:**
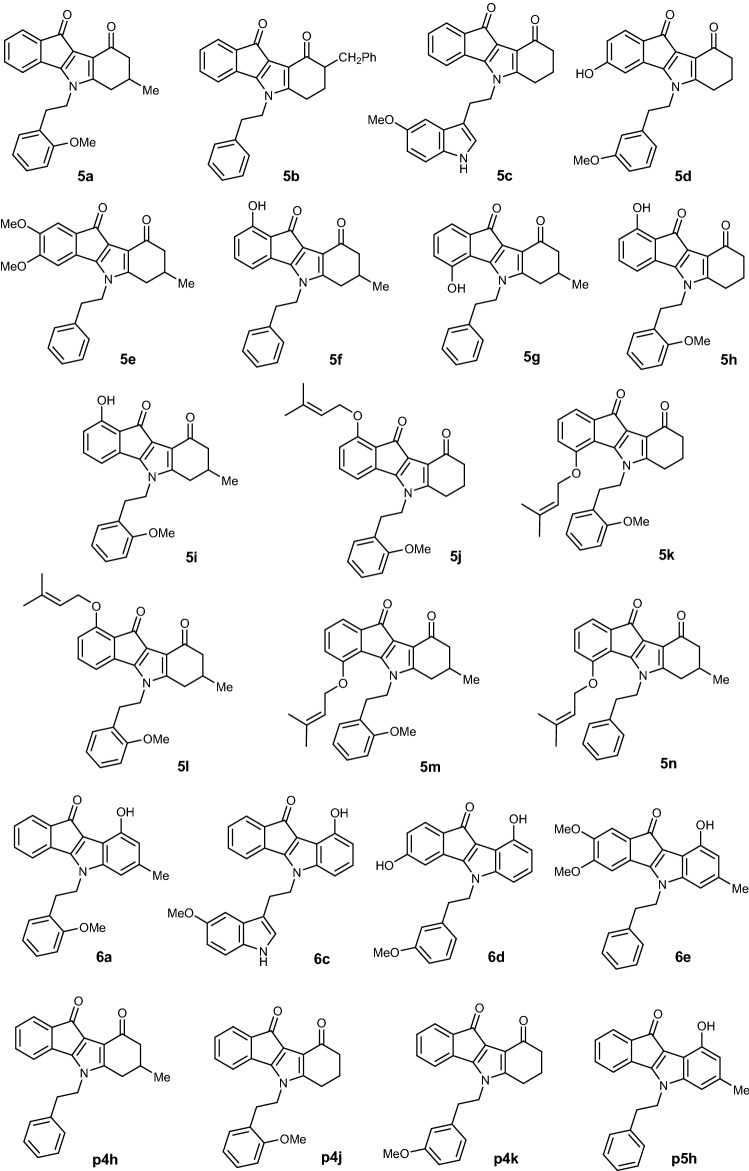
Structures of all studied ketonic and phenolic indeno[1,2-*b*]indole derivatives (compounds **p4h**, **p4j**, **p4k** and **p5h** were already published^19, 20^).

Altogether, 18 ketonic and phenolic indeno[1,2-*b*]indole derivatives were synthesized, and their various substituents are shown in Fig. 1.

### Structure–activity relationship (SAR) study of indeno[1,2-*b*]indole derivatives: ABCG2 inhibition and cell cytotoxicity

Indeno[1,2-*b*]indole derivatives have been described as promising potent ABCG2 inhibitors^19, 20^. Structural optimization was done with compounds initially synthesized and tested on ABCG2^20^. We introduced structural modifications favoring ABCG2 inhibition to obtain the eighteen novel derivatives (Fig. 1).

**Figure 2:**
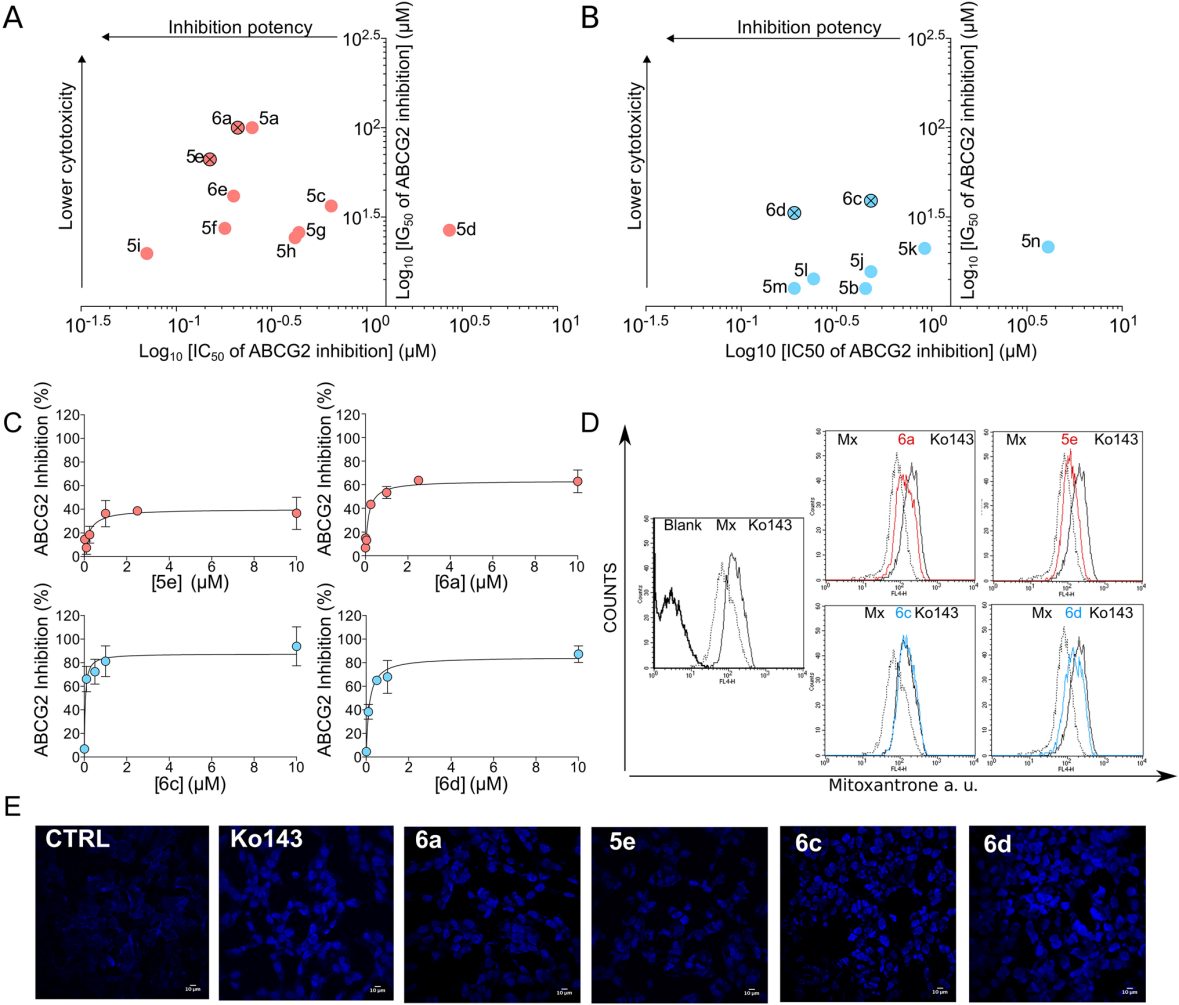
Inhibition potency and cytotoxicity of compounds with the best therapeutic ratios. (**A**) Partial inhibitors (red) and (**B**) complete inhibitors (blue). (**C**) Representative IC_50_ curves of the partial and complete inhibitors. (**D**) Representative flow cytometry histograms of mitoxantrone accumulation in HEK293-*ABCG2* cells. Overlay of histograms obtained by compounds at 10 µM mitoxantrone and Ko143. (**E**) Representative confocal microscopy images of Hoechst 33342 (1 µM) accumulation in HEK293-*ABCG2* cells, using the program ImageJ2 (URL: https://imagej.net/Fiji). Effect produced by compounds at 10 µM compared to the reference inhibitor Ko143 at 0.5 µM.

As shown in Supplementary Fig. S6, all compounds inhibited the transport activity of ABCG2 at 1 and 10 µM concentrations. In addition, many compounds showed a very high potency of inhibition, saturating at a concentration of 1 µM. To perform a precise SAR, a range of concentrations was assayed for each compound to determine and then compare their half maximal inhibition (IC_50_) values (Fig. 2C and S7). These results are summarized in Table 1.

**Table 1:** Inhibition of ABCG2-mediated efflux and cytotoxicity. The inhibition of ABCG2-mediated mitoxantrone efflux was assayed in HEK293-*ABCG2* cells.

Compound	IC_50_ (µM) ± SD^a^	I_MAX_ (%) ± SD^b^	IG_50_ (µM) ± SD^c^	TR^d^
**5a**	0.25 ± 0.01	72.55 ± 3.72	> 100	> 393.60
**5b**	0.45 ± 0.10	112.80 ± 3.67	12.58 ± 5.12	28.10
**5c**	0.65 ± 0.03	60.05 ± 4.04	36.50 ± 20.08	56.41
**5d**	2.71 ± 0.14	68.50 ± 0.68	26.70 ± 5.52	9.85
**5e**	0.15 ± 0.08	36.88 ± 1.16	66.50 ± 7.78	453.11
**5f.**	0.18 ± 0.02	48.39 ± 0.80	27.35 ± 7.57	153.24
**5g**	0.44 ± 0.03	65.23 ± 0.94	25.95 ± 14.35	58.79
**5h**	0.42 ± 0.07	69.62 ± 1.14	24.25 ± 3.32	57.21
**5i**	0.07 ± 0.02	62.83 ± 0.48	19.80 ± 3.39	293.66
**5j**	0.48 ± 0.08	85.57 ± 2.59	15.65 ± 7.28	32.86
**5k**	0.92 ± 0.10	115.70 ± 2.36	21.10 ± 2.55	22.85
**5l**	0.24 ± 0.03	82.35 ± 3.44	14.25 ± 0.07	59.72
**5m**	0.19 ± 0.07	87.42 ± 2.46	12.60 ± 7.22	66.23
**5n**	4.09 ± 0.56	133.18 ± 3.35	21.50 ± 5.09	5.26
**6a**	0.21 ± 0.03	62.65 ± 2.30	> 100	> 469.65
**6c**	0.48 ± 0.10	90.51 ± 8.07	39.05 ± 26.94	81.35
**6d**	0.19 ± 0.04	84.79 ± 2.51	33.30 ± 13.86	176.12
**6e**	0.20 ± 0.04	66.80 ± 1.79	41.50 ± 33.52	212.38
**p4h**^e,19^	0.23 ± 0.02	100.00 ± 21.00	> 100	> 435.00
**p4j**^e,19^	0.21 ± 0.07	106.00 ± 21.00	27.20 ± 0.70	130.00
**p4k**^e,19^	0.31 ± 0.09	78.00 ± 14.00	12.70 ± 3.10	41.00
**p5h**^e,20^	0.15 ± 0.01	85.00 ± 11.00	54.00 ± 14.00	360.00

^a^The affinity of inhibitor interaction was expressed as IC_50_ values (compound concentrations giving a half-maximal inhibition).

^b^The maximal inhibition percentage (I_MAX_) was determined by IC_50_ fitted curves.

^c^The cytotoxicity was expressed as IG_50_ values (compound concentrations giving a half-maximal cell viability).

^d^The therapeutic ratio (TR) corresponds to the ratio between IG_50_ and IC_50_ values.

^e^Experimental data of compounds **p4h**, **p4j**, **p4k** and **p5h** correspond to those previously published.

The addition of a methoxy substituent in the *ortho* position of *N*^5^-phenethyl (**5a**) did not increase affinity (IC_50_ of 0.25 μM) by comparison with **p4h** (IC_50_ of 0.23 μM)^19^. The introduction of a polar substituent (hydroxyl) at position 1 (**5i**) increased by 3.5-fold the potency of ABCG2 inhibition comparing with **5a**, as determined by IC_50_ values (**5i** 0.07 μM vs **5a** 0.25 μM). In contrast with **p4j** (0.21 μM)^19^, we observed a 2-fold lower affinity for **5h** (0.42 μM). Moving the hydroxyl substituent to position 4 was unfavorable, decreasing by 2.5-fold the compound’s affinity (**5f** 0.18 μM vs **5 g** 0.44 μM). A relevant negative effect was observed when the hydroxyl substituent was located in position 3 (**5d** toward **p4k**), IC_50_ of 2.71 µM vs 0.31 µM^19^, respectively.

We found that the presence of *O*-prenylated group at position 4 led to a lower affinity, increasing 18-fold the IC_50_ values (**p4h**^19^ 0.23 μM vs **5n** 4.09 μM). However, the replacement of hydroxyl by *O*-prenyl at position 1 either decreased the affinity (**5i** 0.07 vs μM **5l** 0.24 μM) or did not produce any effect (**5h** 0.42 μM vs **5j** 0.48 μM). Shifting this substituent to position 4 either reduced the compound’s affinity (**5j** vs **5k**, 0.48–0.92 μM) or did not produce any effect (**5l** vs **5m**).

The presence of a methoxy group in *ortho* of *N*^5^-phenethyl increased the inhibition potency 22-fold (**5m** vs **5n**) and 2.5-fold (**5i** vs **5f**). With a methyl substituent at position 7 of the D-ring, the affinity was increased from 5- to 6-fold (**5m** vs **5k** and **5i** vs **5h**). This favorable effect was also found in **5l** by comparison to **5j** (2-fold). In addition, phenolic derivatives with a methoxy substituent in *meta* of *N*^5^-phenethyl (**6d**) were 14-fold more potent than the ketonic derivative **5d** (0.19 vs 2.71 μM).

### Selection of the best inhibitors

Considering two important parameters, potency of ABCG2 inhibition and intrinsic cell cytotoxicity, we determined the therapeutic ratio (TR) values (Table 1). The highest TRs were observed with the less cytotoxic compounds **5e** and **6a**, with TRs higher than 400.

Surprisingly the indeno[1,2-*b*]indole derivatives showed different patterns related to the maximal inhibition (I_MAX_). Most derivatives produced a partial inhibition, with maximal inhibition of approximately 60%. To better understand this effect, the compounds were classified as either complete (I_MAX_ ≥ 80% of ABCG2 inhibition) or partial (I_MAX_ ≤ 80% of ABCG2 inhibition) inhibitors, by a threshold at 80% on I_MAX_ values.

Considering that the best parameter to describe a promising inhibitor is the TR, the two best inhibitors of each class were selected. As shown in Fig. **2A**, **5e** and **6a** were selected as the best partial inhibitors, showing TRs of > 470 and 453, respectively (Table 1). In addition, **6c** and **6d** were selected as the best complete inhibitors of the study, with TRs of 81 and 176, respectively (Fig. 2B and Table 1). These differences between partial and complete ABCG2 inhibitors identified by flow cytometry-based assays are illustrated by the IC_50_ curves of inhibition (Fig. 2C), and by flow cytometry histograms (Fig. 2D).

To confirm this pattern of inhibition, a different ABCG2 substrate, Hoechst 33342, was used. The intracellular fluorescence of this substrate was recorded by confocal microscopy (Fig. 2E). The Hoechst 33342-mediated efflux by ABCG2 confirmed the existence of two different patterns in the maximal inhibition produced by these derivatives. In addition, this results suggest that these indeno[1,2-*b*]indole derivatives are not substrate-specific inhibitors.

### Indeno[1,2-*b*]indole derivatives are not transported by ABCG2

**Figure 3:**
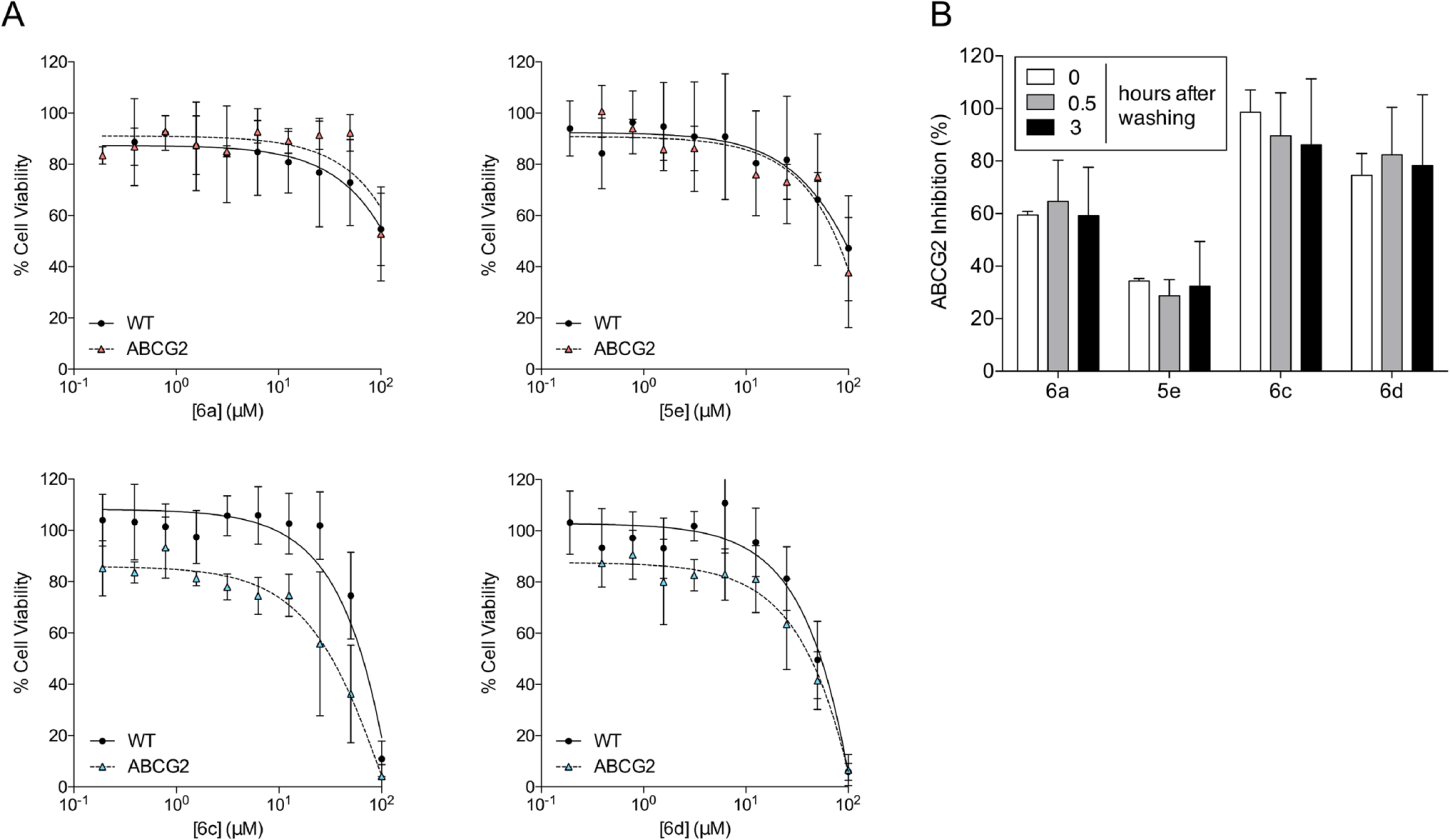
Cytotoxicity of inhibitors and washout assay. Cell viability was determined by MTT assay. (**A**) Cell viability of HEK293-*ABCG2* cells and HEK293 (wild-type) control cells upon 72 h treatment with inhibitors at increasing concentrations (0.1–100 µM), as indicated. (**B**) Washout assay performed on HEK293-*ABCG2* cells with the compounds at 10 µM. The white bars represent the classical experiment (30 min of concomitant incubation with compounds and mitoxantrone before analysis), grey and black bars represent pre-treatment with compounds for 30 min followed by washing procedure at 0.5 and 3 h, as described in methods. The data are the mean ± SD of three independent experiments performed in duplicate.

The selected indeno[1,2-*b*]indole derivatives showed a low intrinsic cytotoxicity, an advantageous characteristic when screening new inhibitors. However, the compounds produced a quantifiable cytotoxic effect at higher concentrations, leading us to check for possible cross-resistance. As shown in Fig. 3A, the intrinsic cytotoxicity produced by **6a** and **5e** was the same in *ABCG2*-transfected cells or control cells, indicating no apparent cross-resistance. In addition, a preferential cell death was observed in *ABCG2*-transfected cells treated with **6c** and **6d**, suggesting a mild collateral sensitivity (CS) effect. CS is characterized by compounds that trigger a preferential cell death in cells overexpressing ABC transporters and constitute a promising strategy to target resistant cancer cells; however, this suggestion needs further investigation. In summary, these results indicate that all the four indeno[1,2-*b*]indole derivatives are not transported by ABCG2.

To support the hypothesis of absence of transport, a washing assay was used to investigate if the compounds retain their inhibitory effect after removal from the culture medium. As shown in Fig. 3B, even after 3 h in the absence of compounds in the culture medium, the potency of inhibition was similar. Together, these data confirm that these compounds are not transported by ABCG2.

### Selected indeno[1,2-*b*]indole derivatives sensitize ABCG2 expressing cells to anticancer drugs

**Figure 4:**
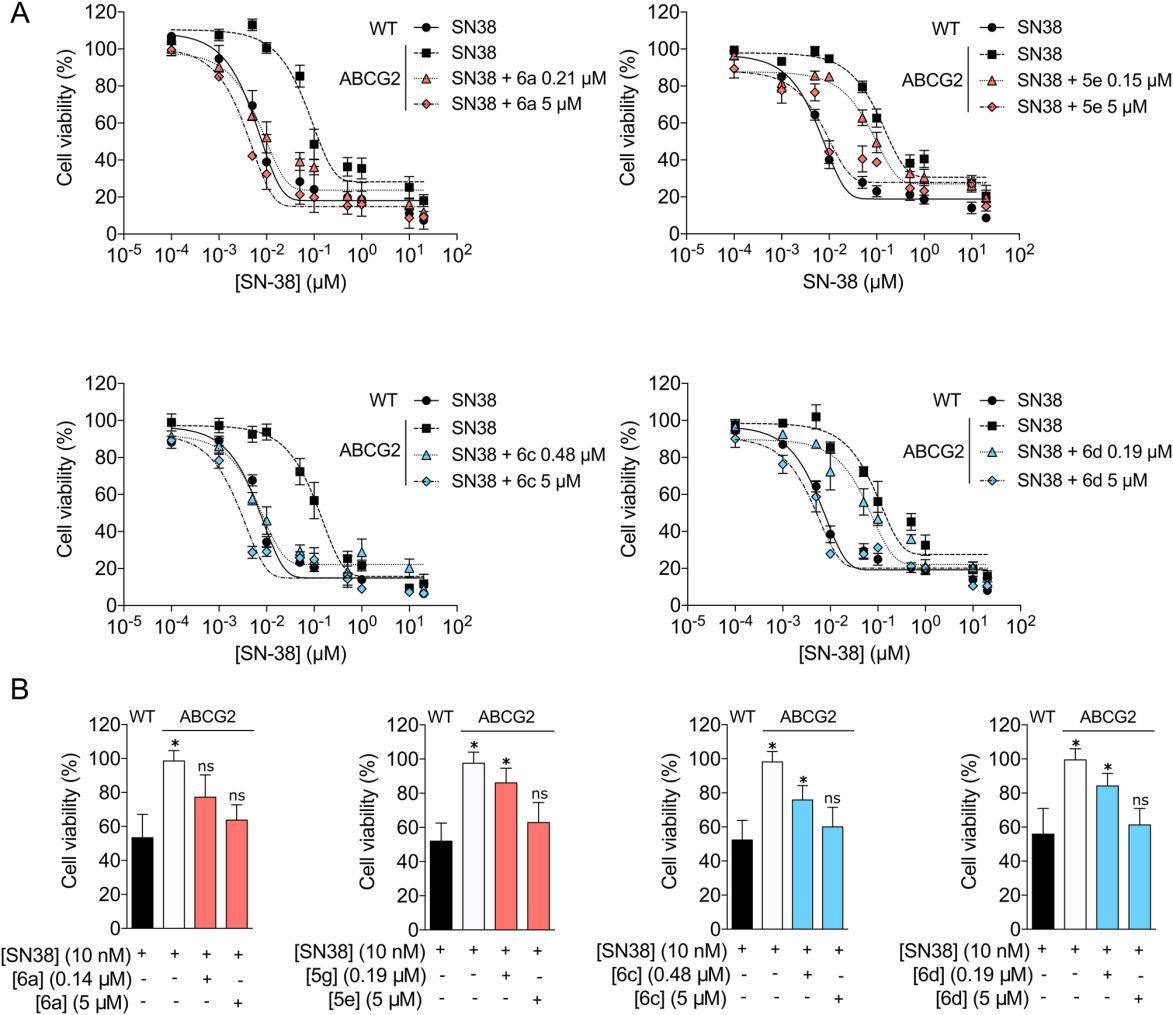
Sensitization of transfected and cancer cells overexpressing ABCG2 to SN-38. Cell viability was determined by MTT assay. (**A**) Cell viability of HEK293-*ABCG2* and HEK293 (wild-type) control cells upon 72 h treatment with SN-38 at increasing concentrations, as indicated, and HEK293-*ABCG2* cells upon co-treatment with SN-38 and inhibitors at either IC_50_ values or 5 µM. (**B**) Cell viability of H460MX20 and H460 control cells upon 72 h treatment with SN-38 at 10 nM, as indicated, and H460MX20 cells upon co-treatment with SN-38 and inhibitors at either IC_50_ values or 5 µM. The data are the mean ± SEM of three independent experiments performed in triplicate and compared using the Student's *t* test (2-sided) for independent samples. *p < 0.05 were considered significant for all tests.

To confirm that the indeno[1,2-*b*]indole derivatives sensitize the cells overexpressing ABCG2 to a chemotherapeutic, a cell viability assay was performed after 72 h of co-treatment using the active metabolite of Irinotecan, SN-38, with inhibitors in two conditions: a concentration that corresponds to the IC_50_ value and a saturation concentration. As shown in Fig. 4A, all compounds chemosensitized the *ABCG2*-transfected cells. The fold reversal values are summarized in Supplementary Table S1. In addition, these results also suggest that these compounds are not metabolized during a period of 72 h.

Surprisingly, full chemosensitization was obtained with both partial inhibitors (Fig. 4A and Supplementary Table S1), suggesting that partial inhibition is enough to produce a full reversion of the chemoresistant phenotype. To confirm this hypothesis, a lung cancer cell line was used. The parental cancer cells, H460, and the cell line overexpressing ABCG2, H460MX20, were treated with SN-38 in the absence or presence of inhibitors (Fig. 4B). A similar behavior was observed, confirming that a partial inhibitor can chemosensitize cancer cells overexpressing ABCG2.

### Specificity of indeno[1,2-*b*]indole derivatives toward ABCG2

**Figure 5:**
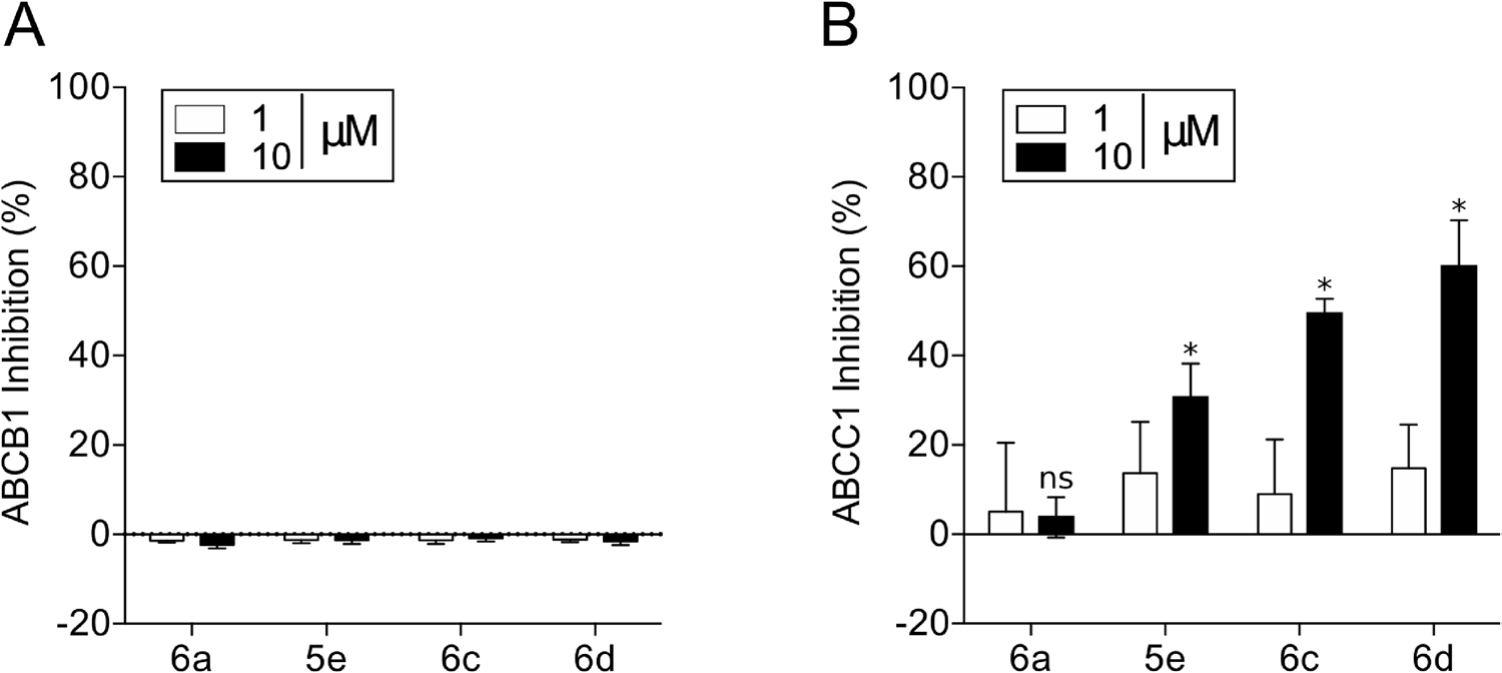
Selectivity of indeno[1,2-*b*]indole derivatives toward ABCG2. (**A**, **B**) Effect of compounds at 1 and 10 µM on ABCB1 (P-gp) for rhodamine 123 transport inhibition and on ABCC1 (MRP1) for calcein-AM transport inhibition. The data are the mean ± SD of three independent experiments performed in duplicate and compared using the Student's *t* test (2-sided) for independent samples. *p < 0.05 were considered significant for all tests.

The four selected indeno[1,2-*b*]indole derivatives were tested as inhibitors against two other important multidrug ABC transporters, ABCB1 (P-gp) and ABCC1 (MRP1). None of these four compounds inhibited P-gp-mediated transport (Fig. 5A). In contrast, three compounds produced a partial inhibition of MRP1-mediated transport at 10 μM, ranging from 30 to 60% of inhibition (Fig. 5B). These data suggest that indeno[1,2-*b*]indole derivatives inhibit preferentially ABCG2. Nevertheless, only **6a** can be described as a specific ABCG2 inhibitor, while the selectivity toward ABCG2 for the other compounds is linked to the concentration used.

### Effect of indeno[1,2-*b*]indole derivatives on the ATPase activity and 5D3 binding to ABCG2

**Figure 6:**
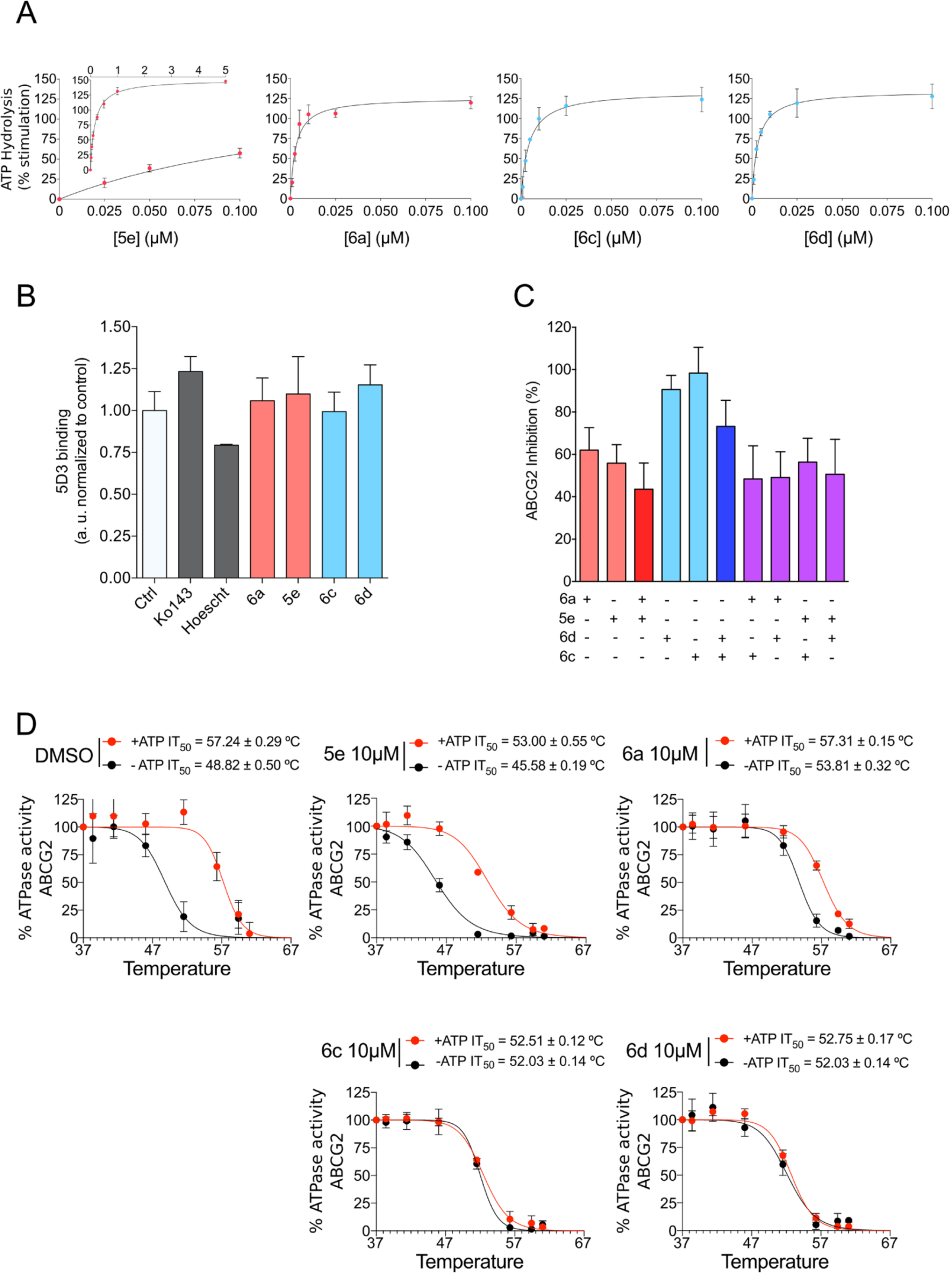
Studies on ABCG2 ATPase activity, binding of conformational antibody 5D3, biomodulation assay and thermostabilization assay. (**A**) Effect of compounds at increasing concentrations (0.001–5 µM) on basal ABCG2 ATPase activity. (**B**) Effect of compounds at 10 µM on the binding of conformational antibody 5D3. (**C**) Bimodulation assay using partial and complete inhibitors at 10 µM on ABCG2 transport activity using mitoxantrone as a substrate. (**D**) Thermostabilization assay with partial and complete inhibitors at saturating concentration of 10 µM. The data are the mean ± SD of three independent experiments performed in duplicate.

The transport activity of ABC transporters is dependent on ATP binding and hydrolysis. To further investigate the mechanism of inhibition, the ATPase activity was evaluated using High-Five insect cell total membranes overexpressing ABCG2 after incubation with indeno[1,2-*b*]indole derivatives at increasing concentrations. These compounds showed a strong stimulation effect on ABCG2 ATPase activity, with EC_50_ values of approximately 0.003 µM with phenolic compounds (**6a**, **6c** and **6d**) and 0.17 µM for ketonic compound (**5e**) (Fig. 6A and Supplementary Table S2). A second approach was used to evaluate possible conformational changes induced by these derivatives using the conformation-sensitive antibody 5D3, which recognizes extracellular loops of the ABCG2 transporter. As shown in Fig. 6B, the indeno[1,2-*b*]indole derivatives did not promote conformational changes that affect the 5D3 binding. Together, these results suggest that the binding of these compounds in the drug-binding pocket of ABCG2 promotes conformational changes affecting ATPase activity, but these conformational changes do not affect the extracellular loops recognized by the 5D3 antibody.

### Bimodulation and thermostabilization assays

As previously described, the compounds were classified as either complete (I_MAX_ ≥ 80%) or partial inhibitors (I_MAX_ ≤ 80%). Partial inhibition at a saturating concentration allows the combination of these inhibitors (**6a**, **5e**) with complete inhibitors (**6c**, **6d**) to check for additive or antagonistic effects. As shown in Fig. 6C, all possible combinations decrease the maximal inhibition, indicating a possible antagonism effect between the indeno[1,2-*b*]indole derivatives. The decrease in the maximal inhibition was more evident when combining a partial and a complete inhibitor (purple bars). Since a partial inhibitor moderately suppresses the effect of a complete inhibitor, we hypothesize that compounds **6a** and **5e** may prefer to bind at sites that overlap with the binding sites of the complete inhibitors **6c** and **6d**.

The thermal stability assay showed that the indeno[1,2-*b*]indole derivatives trigger ABCG2 conformational changes stabilizing the protein structure (Fig. 6D). The partial inhibitors (**5e** and **6a**) presented a moderate effect compared to complete inhibitors (**6c** and **6d**). This effect can be observed by comparing the IT_50_ (temperature that reduces 50% of the ATPase activity) values from curves with or without ATP (Fig. 6D). For complete inhibitors, it was observed an overlap of both curves (-ATP and + ATP), suggesting a plausible explanation for the complete inhibition of substrate transport observed by flow cytometry.

### Docking analysis of indeno[1,2-*b*]indole derivatives with ABCG2

**Figure 7:**
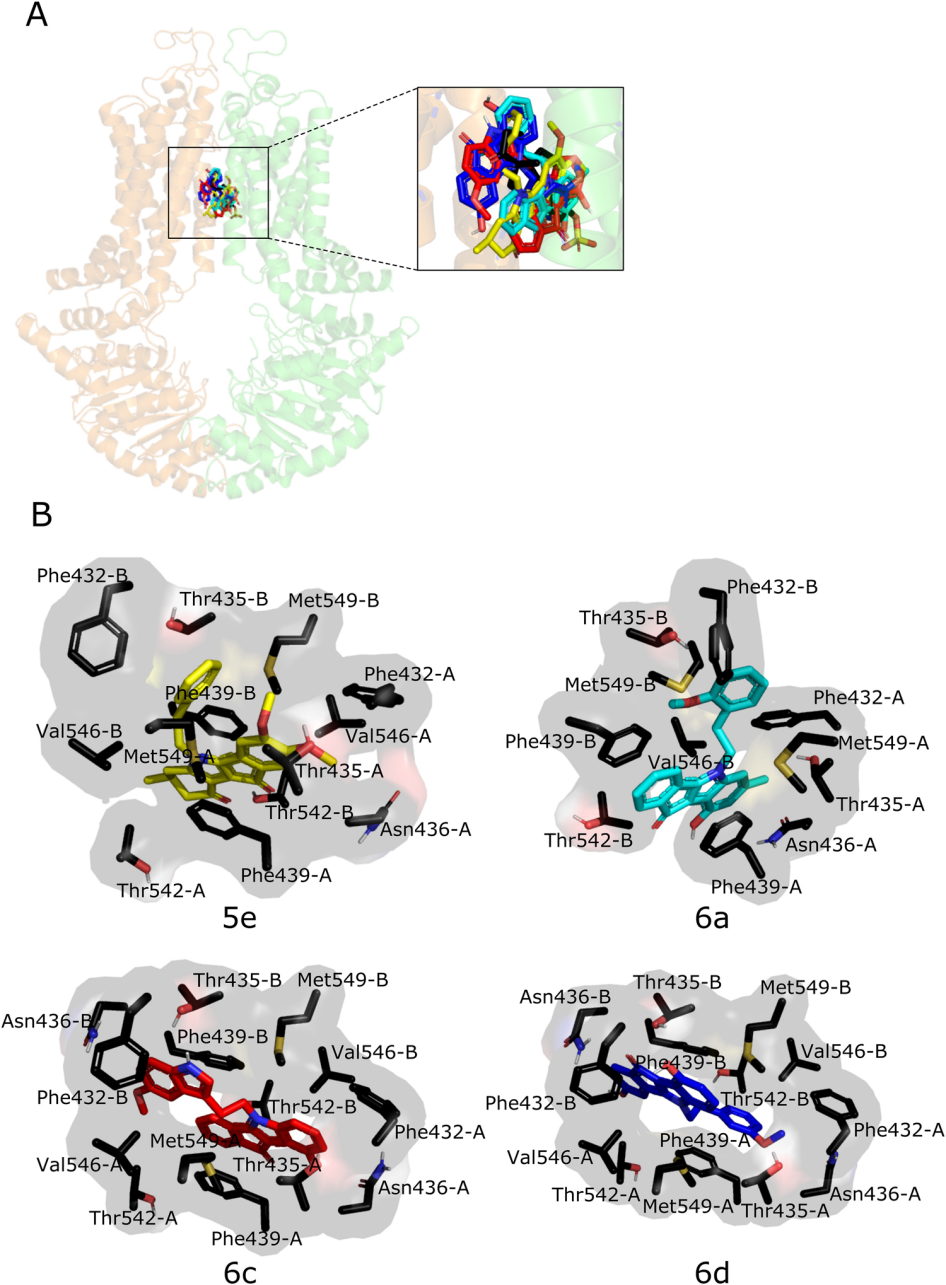
Docking analysis in human ABCG2 (PDB 6HCO). (**A**) Representative docking poses of the inhibitors overlapping the binding site of estrone-3-sulfate (E3S – black, **6c**—red, **6d**—blue, **6a**—cyan and **5e**—yellow), in stick representation. The two monomers are colored differently, orange (chain A) and green (chain B). (**B**) Interacting residues on the best energy score pose of the inhibitors are represented as stick models. The images were generated using the program PyMOL Molecular Graphics System, Version 1.2r3pre, Schrödinger, LLC (URL: https://pymol.org/2/).

Molecular docking was performed in order to gain insight into the structural determinants responsible for the behavior of partial and complete inhibitors with the ABCG2 transporter. The structure used for this study was the PDB 6HCO which shows a molecule of estrone-3-sulfate co-crystallized^29^. Docking results showed that the indeno[1,2-*b*]indole derivatives **5e**, **6a**, **6c** and **6d** share the same binding site as the estrone-3-sulfate (Fig. 7A), with similar binding energies, ranging from − 12.8 to − 12.3 kcal/mol and also share 6 common amino acids residues (Thr435, Asn436, Phe439, Thr542, Val546 and Met549) with estrone-3-sulfate (Supplementary Table S3). In agreement, recent studies showed that substrates (e.g. mitoxantrone, SN38, estrone-3-sulfate) and inhibitors (e.g. FTC, Ko143, MZ29) bind in the same drug-binding pocket in ABCG2^30–32^. This small difference in energy is less than that of a weak hydrogen bond. Therefore, the energy profile of only four inhibitors did not provide a clear picture of their inhibitory profile. For this reason, the inhibitor-transporter interactions for all nine poses were examined carefully to distinguish their specific interactions. Hydrophobic interactions appear to be the most prevalent type of interactions between the central core of indeno[1,2-*b*]indole derivatives and hydrophobic amino acids residues, in particular the most frequent interactions with Phe439. Residues Phe432, Thr435, Asn436, Thr542, Val546 and Met549 are also involved (Fig. 7B). Although the hydrophobic interactions are clearly relevant, it is important to notice that some hydrogen bonds are present. An additional molecular docking analysis was performed with the PDB 6FFC^30^. The results confirmed that all four indeno[1,2-*b*]indole derivatives share the same binding site, showing the same interactions observed with the PDB 6HCO (Supplementary Fig. S8). Therefore, a molecular docking analysis was insufficient to highlight the residues responsible for discriminating between partial and complete inhibitors.

## Discussion

Currently, clinical trials using ABC drug transporter inhibitors were conducted exclusively targeting P-glycoprotein. The majority of these clinical trials failed to improve the chemotherapy efficacy, probably due several factors, including the myriad of multidrug resistance mechanisms found in tumors, and the absence of standardized protocols to identify a subset of patients based on tumor expression levels of ABC transporters. Considering the clinical relevance of P-glycoprotein and ABCG2, and the availability of many potent P-glycoprotein inhibitors, there is an urgent need for the identification of potent and specific ABCG2 inhibitors suitable for clinical use along with P-glycoprotein inhibitors.

As previously reported, indeno[1,2-*b*]indole derivatives that were initially designed as human casein kinase II (CK2) inhibitors can be converted into potent ABCG2 inhibitors^19, 20^. The novel screened compounds confirmed that derivatives containing *N*^5^-phenethyl on the C-ring and hydrophobic groups on the D-ring produce the most powerful ABCG2 inhibitors (Supplementary Fig. S9).

The potency of inhibition, normally reported as the IC_50_ of inhibition, gives a general idea about the affinity of compounds. Considering the IC_50_ values alone, the best indeno[1,2-*b*]indole derivative identified was the compound **5i** (0.07 µM). However, this compound was one of the most cytotoxic (IG_50_ of 20 µM). Since the evidence that some potent and specific ABCG2 inhibitors, as FTC, cannot be used in pre-clinical models because of their neurotoxic effects^13^, an evaluation of intrinsic cell cytotoxicity has been included in studies describing new ABCG2 inhibitors. Considering that intrinsic cytotoxicity (IG_50_) and potency of inhibition (IC_50_) are the two most important parameters for screening new ABCG2 inhibitors, the concept of a therapeutic ratio (TR) was introduced^16–20, 29, 33^. Therefore, we selected the best inhibitors based exclusively on their TR values (Fig. 2A,B).

Another important consideration concerning ABCG2 inhibitors is their maximal inhibition. Many compounds produce a partial inhibition, such as 6-prenylchrysin^34^, methoxy stilbenes^17^, some chalcones^18^ and chromones^15^. These compounds reach approximately 70% inhibition. Some of the indeno[1,2-*b*]indole derivatives screened in this study also showed this effect when mitoxantrone was used as a substrate. The differences were explored in this study; we classified the inhibitors as either partial or complete (Table 1). To our knowledge, this is the first study investigating such characteristics of ABCG2 inhibitors. We selected the two best partial and complete inhibitors based on their TR values to continue characterization of the effects (Fig. 2A,B).

Surprisingly, the partial inhibitors **6a** and **5e** exhibited TR values (> 450) higher than the complete inhibitors **6c** and **6d**, with TRs of 176 and 81, respectively. In order to determine if a partial inhibitor would be useful in pre-clinical studies, the effects of both classes of compounds were evaluated in chemosensibilization assays. Despite their partial inhibition, **6a** and **5e** efficiently chemosensitize cells overexpressing ABCG2 (Fig. 4, Supplementary Table S1). This effect was confirmed in lung cancer cells overexpressing ABCG2, constituting a proof of concept that partial inhibitors can be useful clinically, since the chemoresistance phenotype was fully reversed. Further, the indeno[1,2-*b*]indole derivatives are not transported by ABCG2, and this is true for both partial and complete inhibitors (Fig. 3A,B). Additionally, the indeno[1,2-*b*]indole derivatives are not substrate-specific inhibitors, since they inhibit mitoxantrone (Fig. 2D and Supplementary Fig. S6 and Table 1), Hoechst 33342 (Fig. 2E) and SN-38 (Fig. 4A,B, Supplementary Table S1) efflux-mediated by ABCG2.

ABCG2 inhibitors are also classified as either selective toward ABCG2 or dual inhibitors, which include compounds that inhibit more than one ABC transporter. Despite the selectivity of the indeno[1,2-*b*]indole derivatives toward ABCG2 in comparison to P-gp, only compound **6a** was completely selective to ABCG2, whereas **5e**, **6c** and **6d** partially inhibited MRP1-mediated transport (Fig. 5B). These data are in agreement with those previously described for indeno[1,2-*b*]indole derivatives, which also showed a non-selective effect but a higher affinity for ABCG2 than MRP1^19,20^. This dual effect of ABCG2/MRP1 inhibition is not common, because dual inhibition is normally observed for compounds that inhibit ABCG2 and P-gp^35^. Some of these non-selective inhibitors, such as GF120918, present higher affinity for P-gp than ABCG2^36^. However, other compounds, such as the reference inhibitor of ABCG2, Ko143, show higher affinity for ABCG2 than P-gp^14^.

The transport of substrates through the cellular membrane mediated by ABC transporters is coupled to the binding and hydrolysis of ATP^2^. In contrast to the effect of Ko143, which inhibit the ATPase activity of ABCG2 transporter^14^, the indeno[1,2-*b*]indole derivatives were found to stimulate ATPase activity (Fig. 6A). Generally, compounds that inhibit the transport of substrates also inhibit the ATPase activity, however, several chemically unrelated ABCG2 inhibitors stimulate the ATPase activity, such as: sildenafil^37^, curcumin analogs^38^, alectinib^39^, stilbenes^17^, icotinib^40^, lapatinib^41^, imatinib^42^, AST1306^43^, WHI-P154^44^, telatinib^45^ and selonsertib^46^. This effect is even more complex for some compounds, as nilotinib, which produces a dual effect: ATPase stimulation at low concentrations and ATPase inhibition at high concentrations^47^. In addition, some classical substrates, as mitoxantrone^48^, produce a mild stimulation of the ATPase activity of ABCG2 (40%), when compared to prazosin (100%). Although some of these compounds that stimulate the ATPase activity were suggested to be transported by ABCG2, further investigations are required to understand why certain ABCG2 modulators stimulate the ATPase activity. A plausible explanation about this complexity probably is related to the recent evidences about the overlap of substrate and inhibitor binding sites on ABCG2^32^.

Considering only the effect on ATPase activity, the affinity of phenolic compounds was approximately tenfold higher than Ko143. The observed stimulation is similar to previous reports indicating that indeno[1,2-*b*]indole derivatives either stimulated or did not affect the ATPase activity^20^, and suggest allosteric interactions between the nucleotide-binding domain and the binding site of these new derivatives. ABCG2 inhibitors have also been found to trigger conformational changes that increase the 5D3 antibody binding^49^. However, the indeno[1,2-*b*]indole derivatives did not have any effect on 5D3 binding (Fig. 6B), suggesting an absence of allosteric interactions between the extracellular loop ECL3 and their respective binding sites.

Interestingly, combining indeno[1,2-*b*]indole derivatives produced an antagonistic effect (Fig. 6C), suggesting a partial binding site overlap and competition. To verify this hypothesis, docking experiments were carried out using the cryo-EM structure of ABCG2 (PDB 6HCO co-crystallized with estrone-6-sulfate)^29^. The analysis showed that the inhibitors share the same binding site and three amino acid residues appear to be important for the inhibition behavior, Phe439 (the most interactive) followed by Val546 and Met549. Hydrophobic interactions are the most prevalent, with the most frequent interaction being π-π stacking between the phenyl ring of the Phe amino acid residue and the polycyclic core of the inhibitors. Asn436 in both ABCG2 monomers is involved in this interaction with the hydrophilic groups from the compounds, such as carbonyl, indolic nitrogen, methoxy and phenolic groups. This amino acid residue was already reported to be involved in interactions with an ABCG2 inhibitor (MZ29) in an ABCG2 cryo-EM structure^30^, and its mutation resulted in the loss of substrate transport (estrone-3 sulfate)^29^. The indeno[1,2-*b*]indole derivatives promoted conformal changes thus stabilizing the protein structure (Fig. 6D). The most pronounced effect was observed with the complete inhibitors **6c** and **6d**, suggesting a possible relationship with a more closed form of protein and the complete inhibition.

Considering the chemical structure of the indeno[1,2-*b*]indole derivatives, it is important to note that compound **6d** (complete inhibitor) has a methoxy group in the *meta* position and compound **6a** (partial inhibitor) has that group in the *ortho* position on the phenethyl moiety. Docking results for **6d** showed interactions between 3-OMe-phenethyl group and Thr435-A, Phe432-A and Val546-B. In the case of **6a**, interactions between 2-OMe-phenethyl group and Thr435-B, Phe432-B and Met549-B were noted. The different behavior of compounds **6a** and **6d** could also be explained by interactions induced by the 7-methyl group present in **6a** but not in **6d** or by the 3-hydroxyl group present in **6d** but not in **6a**. As **6d**, multiple interactions are present between the indolic part of **6c** (east part of this second complete inhibitor, Fig. 7B) and residues Thr435-A, Val546-B, Phe439-A, Phe432-A and Asn436-A. The result observed with **5e** (partial inhibitor) showed that the presence of a ketonic ring D does not interact with any amino acid residues. The presence of aromatic rings A, B and C seemed to be as well important with several interactions (e.g. Phe439-A, Phe439-B, Met549-B, Phe432-A, Asn436-A, Thr435-A and Val546-B).

In summary, we describe in this report new indeno[1,2-*b*]indole derivatives that are potent ABCG2 inhibitors. These compounds chemosensitize cancer cells overexpressing ABCG2. Their very high TR values suggest that they are promising compounds that could be used in pre-clinical assays. Our findings are similar to those reported for chromones, which showed high TR values during in vitro screening^16^ and were successfully applied in vivo^50^. Further structural investigations are engaged to define more precisely the roles of the ketone function in position 10 (east part of partial inhibitors, Fig. 7B) and of the substituent in position 5 (west part of complete inhibitors, Fig. 7B).

## Materials and methods

### Chemistry

All intermediates and tested compounds used in this study are fully described in the Supporting Information. The chemical reactions were monitored by thin-layer chromatography (TLC) on GF254 plates that were visualized under an ultraviolet lamp (254 nm). Chromatographic separations of the compounds of a mixture were performed on silica gel columns by column chromatography (Kieselgel 300 − 400 mesh). Evaporation of solvent was performed in a vacuum with a rotary evaporator. Melting points were determined on an Electrothermal 9200 capillary apparatus^19, 20^.

The IR spectra were recorded on a PerkinElmer SPECTRUM TWO FT-IR spectrometer. The ^1^H and ^13^C NMR spectra were recorded at 400 MHz on a Bruker DRX 400 spectrometer. Chemical shifts are expressed in ppm (δ) downfield from internal tetramethylsilane (TMS), and coupling constants J are reported in hertz (Hz). The following abbreviations are used: bs, broad singlet; Cquat, quaternary carbons; d, doublet; dd, doubled doublet; dt, doubled triplet; q, quartet; m, multiplet; s, singlet; t, triplet. The mass spectra were performed by direct ionization (EI or CI) on a Thermo Finnigan MAT 95 XL apparatus^19, 20^.

The purity of the final compounds (greater than 95%) was determined by UHPLC-MS on an Agilent 1290 system using an Agilent 1290 Infinity ZORBAX Eclipse Plus C18 column (2.1 mm × 50 mm, 1.8 μm particle size) with a gradient mobile phase of H_2_O/CH_3_CN (90:10, v/v) with 0.1% of formic acid to H_2_O/CH_3_CN (10:90, v/v) with 0.1% of formic acid at a flow rate of 0.5 mL min^−1^, with UV monitoring at the wavelength of 254 nm with a run time of 10 min^19, 20^.

### Cell lines and cultures

NIH3T3, BHK21 and HEK293 wild-type (WT) cells and transfected cells NIH3T3-*ABCB1*, BHK21-*ABCC1* and HEK293-*ABCG2* were kindly provided by Dr. Attilio Di Pietro (IBCP, Lyon, France). The human non-small lung cancer H460 wild-type cells (H460 WT) and mitoxantrone-induced H460 cells overexpressing ABCG2 (H460MX20) were provided by Dr. Attilio Di Pietro (IBCP, Lyon, France) and Dr. Susan Bates laboratory (HIH, Bethesda, MD). The human fibroblast HEK293 cell line was transfected with plasmid coding for human ABCG2 (HEK293-*ABCG2*) as previously described^51^. The NIH3T3/ABCB1 drug-resistant cell line was transfected with human MDR1/A-G185^52^ and BHK21 cells were transfected with plasmid coding for human ABCC1 (BHK21-*ABCC1*)^53^. All cells were maintained in high glucose Dulbecco’s modified Eagle’s medium, supplemented with 10% fetal bovine serum (FBS), 1% penicillin/streptomycin supplemented with either 0.75 mg mL^−1^ G418 (HEK293-*ABCG2*), 20 nM mitoxantrone (H460MX20), 60 ng mL^−1^ colchicine (NIH3T3-ABCB1) or 0.1 mg mL^−1^ methotrexate (BHK21-MRP1) at 37 °C and 5% CO_2_ under controlled humidity.

### Cytotoxicity assays

Cell viability was evaluated with a 3-(4,5-dimethylthiazol-2-yl)-2,5-diphenyltetrazolium bromide (MTT) colorimetric assay^54^. HEK293 WT and HEK293-*ABCG2* cells were seeded into 96-well culture plates at a 1.5 × 10^4^ cells/well density. After overnight incubation, the cells were treated with various concentrations of compounds (0–100 µM) for 72 h at 37 °C under 5% CO_2_. For sensitization tests (reversion), cells were concomitantly treated for 72 h with compounds (at a concentration that corresponds to IC_50_ or a saturation concentration) and an increasing concentration of SN-38 (1 nM–20 µM). Sensitization experiments were also carried out on H460 and H460MX20 cells. For these experiments, H460 and H460MX20 cells were seeded into 96-well culture plates at 1.0 × 10^4^ cells/well density. After overnight incubation, the cells were concomitantly treated with compounds (at a concentration that corresponds to IC_50_ or a saturation concentration) and SN-38 at 10 nM for 72 h at 37 °C under 5% CO_2_. After the treatment, the culture medium was removed and 100 μL of a 0.5 mg.mL^−1^ MTT solution was added. Cells were then incubated for 4 h at 37 °C under 5% CO_2_. The formazan crystals were dissolved with a solution of DMSO/ethanol (1:1) and the absorbance was measured at 570 nm using a Multiscan FC microplate reader (Thermo Scientific). The results were expressed as percentage of viable cells versus control cells (0.1% DMSO, v/v) taken as 100%.

### ABCG2 inhibition assay

ABCG2 inhibition assays were carried out on HEK293 WT and HEK293-*ABCG2* cells. Cells were seeded at the density of 1 × 10^5^ cells/well in 24 well culture plates and incubated for 48 h at 37 °C under 5% CO_2_. Cells were treated with compounds and 10 μM of mitoxantrone for 30 min at 37 °C and 5% CO_2_. After incubation the cells were washed with PBS, trypsinized and resuspended in ice cold PBS. The intracellular drug fluorescence was monitored with a FACS Calibur cytometer (Becton Dickinson) using the FL4 channel, with at least 10,000 events collected. The maximal fluorescence (taken as 100%) was determined with the difference between the mean fluorescence of HEK-*ABCG2* cells incubated with 0.5 μM Ko143 (ABCG2 reference inhibitor) or by using the HEK293 WT cell line as a control and cells without inhibitor (mitoxantrone alone). The percent of inhibition was determined as previously described^18^. For bimodulation assays the cells were concomitantly treated with 10 μM of one or two combined inhibitors under similar conditions as described previously. For washing assays, cells were treated with 10 μM concentrations of compounds for 30 min. Then, the medium with the inhibitor was removed and the cells were washed with PBS and incubated only with culture medium for different periods of time (0.5 and 3 h). After incubation, cells were exposed to 5 µM mitoxantrone for 30 min, and then analyzed by flow cytometry. The percentage of transport inhibition was calculated by the following equation:

$$\% \, Inhibition \, = \, \left( {C \, {-} \, S} \right)/\left( {I \, {-} \, S} \right) \, \times \, 100$$

where “*C”* corresponds to the intracellular fluorescence of cells in the presence of the tested compounds and substrate (mitoxantrone to ABCG2, Rhodamine 123 to ABCB1 and calcein to ABCC1), “*S”* corresponds to the intracellular fluorescence of cells in the presence of substrate alone, and “*I*” corresponds to the intracellular fluorescence of cells in the presence of the substrate and reference inhibitor (Ko143 to ABCG2, GF120918 to ABCB1 and verapamil to ABCC1).

### ABCB1 and ABCC1 inhibition assay

ABCB1 (P-gp) and ABCC1 (MRP1) inhibition assays were carried out on NIH3T3 WT and NIH3T3-*ABCB1* or BHK21 WT and BHK21-*ABCC1* cell lines. Cells were treated with 5 μM rhodamine 123 or 0.2 μM calcein-AM as substrates, and with or without GF120918 (0.5 μM) or verapamil (30 μM) as reference inhibitors, for ABCB1 and ABCC1, respectively. Compounds were assayed at 1 and 10 µM. The same procedure and calculation to determine the percentage of inhibition were used for ABCG2 inhibition assays. The intracellular fluorescence was monitored with a FACS Calibur cytometer (Becton Dickinson) using the FL1 channel.

### Confocal images

Microscopy analysis was carried out on HEK293 WT and HEK293-*ABCG2* cells. Cells were seeded at a density of 1 × 10^5^ cells/well in 24-well culture plates containing coverslips for microscopy and incubated for 48 h at 37 °C under 5% CO_2_. Cells that adhered to the coverslips were treated with compounds and 1 μM of Hoechst 33342 for 30 min at 37 °C and 5% CO_2_. After incubation, the coverslips were removed from the plate and placed on slides for microscopy. The slides were then read in a confocal microscopy Nikon A1R MP + (NIKON, Tokyo, Japan) using an oil-immersed 40X objective (with the numerical aperture of 1.15). A laser of 405 nm was used for excitation and the fluorescence emission was recorded using a bandpass filter of 425–475 nm. The software Nis Elements 4.20 (NIKON, Tokyo, Japan) was used for visualization of the images (https://www.microscope.healthcare.nikon.com/pt_AMS/products/software/nis-elements/viewer).

### 5D3 binding

The 5D3 binding assay was carried out on HEK293-*ABCG2* cells. Cells were seeded at a density of 5 × 10^5^ cells/well in 24 well culture plates and incubated for 48 h at 37 °C under 5% CO_2_. After incubation the cells were washed with PBS, trypsinized, resuspended in PBS and washed 1 × with PBS. The cell pellet was resuspended in 100 µL of PBS containing 4 µL of a BSA solution (1 mg/mL). Cells were treated with compounds at 10 μM for 10 min at 37 °C. The 5D3 primary antibody (Purified mouse anti-human CD338, BD Pharmigen—1:100) was added and incubated at 37 °C for 30 min. After incubation, centrifugation was performed at 1000 × g for 3 min. The cell pellet was resuspended in 100 µL of PBS and the secondary antibody conjugated with phycoerythrin (PE Goat anti-mouse IgG, Abcam—1:200) was added and incubated at 37 °C for 30 min. After incubation, centrifugation was performed at 1000 × g for 3 min and the pellet was resuspended in 300 µL of PBS. The fluorescence was monitored with a FACS Calibur cytometer (Becton Dickinson) using the FL2 channel, with at least 10,000 events collected.

### ATPase activity

The ATPase assay was carried out as previously described^55^. High-Five insect cell total membranes overexpressing ABCG2 were used at a concentration of 5 µg protein/tube in a final volume of 100 µL. The membranes were incubated in assay buffer containing 50 mM Tris–HCl, pH 6.8, 150 mM N-methyl-D-glucamine (NMDG)-Cl, 5 mM sodium azide, 1 mM EGTA, 1 mM ouabain, 2 mM DTT, and 10 mM MgCl_2_, in the presence or absence of sodium orthovanadate (0.3 mM). The protein-buffer mix was treated with the compounds at increasing concentrations (0.001–5 µM) and incubated for 20 min at 37 °C in the presence of ATP (5 mM). The reaction was stopped with the addition of 100 µL of 5% SDS, for color development was added 400 µL of P_i_ solution (sulfuric acid 36.2 N, water, ammonium molybdate and antimony potassium tartrate) and 200 µL of 1% ascorbic acid. The absorbance was measured after 10 min at 880 nm using a spectrophotometer Ultrospec 3100 pro (Amersham Biosciences).

### Thermostabilization assay

The thermal stability assay was performed according to the published^56^. Total membrane prepared from High Five insect cells overexpressing the ABCG2 at 3 µg protein/tube concentration were incubated with assay buffer composed by 50 mM Tris–HCl, pH 6.8, 150 mM *N*-methyl-d-glucamine (NMDG)-Cl, 5 mM sodium azide, 1 mM ouabain and 2 mM DTT in the presence or absence of 0.3 mM orthovanadate at final volume of 50 µL. To evaluate the effect of those inhibitors over thermal stability, each sample was prepared with 12.5 mM MgCl_2_ or 6.25 mM ATP and incubated with a temperature range from 37 to 71 °C for 10 min using a thermocycler C1000 Touch (Bio-Rad, Hercules, CA). After incubation, 10 µL of 25 mM ATP or 50 mM MgCl_2_ (5 and 10 mM final concentration, respectively) was added and incubated at 37 °C/20 min to allow ATP hydrolysis. The reaction was stopped with the addition of 50 µL of Pi solution containing 1% ammonium molibdate, 2.5 N H_2_SO_4_ and 0.014% potassium-antimony tartrate. To evaluate the absorbance, the samples were transferred to a 96 well plate (50 µL/well), added 150 µL of 0.33% sodium ascorbate solution. The absorbance was measured after 15 min incubation at room temperature using the microplate reader Spectramax iD3 (Molecular Devices, San Jose, CA). The sensitive activity to vanadate (V_i_) was calculated as the difference between the activity in the absence of Vi minus the activity in the presence of V_i_ to each temperature.

### Computational methods

Molecular docking studies were performed using the ABCG2 structure deposited in the Protein Data Bank under the ID code 6HCO^29^ and ID code PDB 6FFC^30^. The inhibitors were structurally optimized by applying the Mopac 2016 program (MOPAC2016, James J. P. Stewart, Stewart Computational Chemistry, Colorado Springs, CO, USA, http://OpenMOPAC.net (2016) by using the PM6 method^57^. The structures for ABCG2 and inhibitors were prepared for use with AutoDock Vina^58^ using AutoDock Tools (v.1.5.6)^59^. First, molecular docking was performed in the entire protein considering several space sizes to search the region for the best fitting based on the empirical binding free energy scoring function calculated by Autodock Vina. After the identification of this region, we performed a search for a space size of 23 × 19 × 19 Å for 6HCO and 23 × 22 × 10 Å for 6FFC centered in the middle of the transporter. A maximum number of 9 binding modes was used. Selected side-chain residues were flexible to rotate, i.e. Asn436, Phe432, Met549, Phe439, Val546, Thr435 and Thr542, from both ABCG2 monomers, and the remaining residues were treated as rigid. Docked models of the inhibitors, binding sites and amino acid interactions were visualized with ABCG2 using PYMOL (Molecular Graphics System, Version 1.3, Schrödinger, LLC)^60^.

## Supplementary Information

Supplementary Information.
