# Supplementary material for: Mechanistic basis of breast cancer resistance protein inhibition by new indeno[1,2-b]indoles
Source: Sci Rep. 2021 Jan 19;11:1788. doi: 10.1038/s41598-020-79892-w (PMC7815716; doi:10.1038/s41598-020-79892-w)
Supplement: Supplementary file 1 — Supplementary Information. [file 41598_2020_79892_MOESM1_ESM.docx]

**Supporting Information**

**Mechanistic basis of breast cancer resistance protein (BCRP/ABCG2) inhibition by new indeno[1,2-*b*]indoles**

**Diogo Henrique Kita
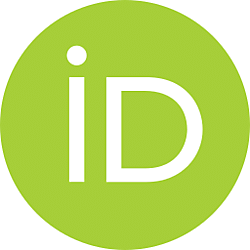
^#,1,2^, Nathalie Guragossian^#,3^, Ingrid Fatima Zattoni
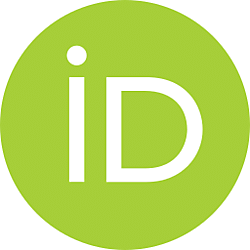
^1^, Vivian Rotuno Moure
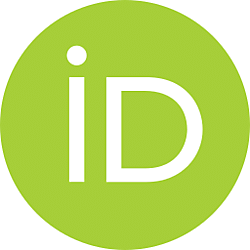
^1,4^, Fabiane Gomes de Moraes Rego
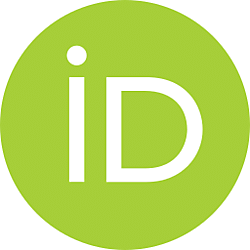
^4^, Sabrina Lusvarghi^2^, Thomas Moulenat^3^, Billel Belhani
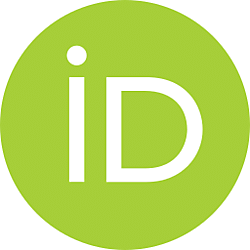
^5^, Geraldo Picheth
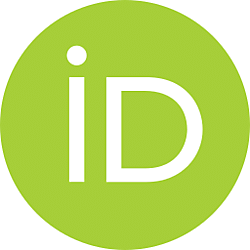
^4^, Sofiane Bouacida
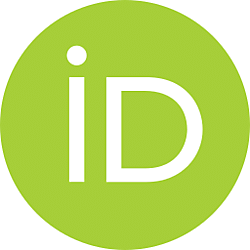
^6,7^, Zouhair Bouaziz
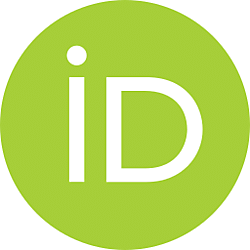
^3^, Christelle Marminon
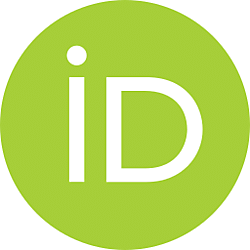
^3,8^, Malika Berredjem
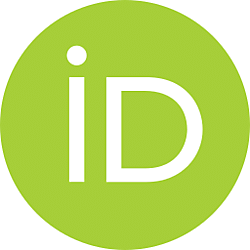
^5^, Joachim Jose
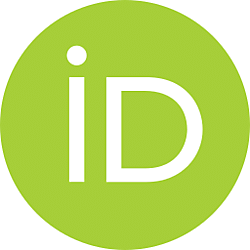
^9^, Marcos Brown Gonçalves
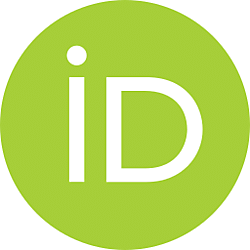
^10^, Suresh V. Ambudkar
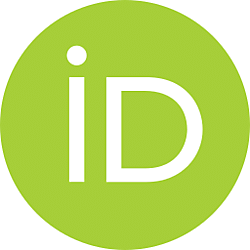
^2^, Glaucio Valdameri
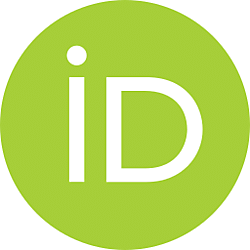
*^,#,1,4^ & Marc Le Borgne
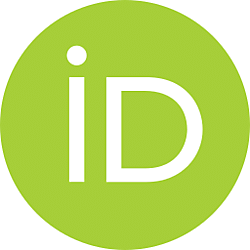
*^,#,3,8^**

^1^Pharmaceutical Sciences Graduate Program, Laboratory of Cancer Drug Resistance, Federal University of Parana, 80210-170 Curitiba, PR, Brazil. ^2^Laboratory of Cell Biology, Center for Cancer Research, National Cancer Institute, National Institutes of Health, Bethesda, Maryland, USA. ^3^EA 4446 Bioactive Molecules and Medicinal Chemistry, Faculté de Pharmacie - ISPB, SFR Santé Lyon-Est CNRS UMS3453 - INSERM US7, Université Claude Bernard Lyon 1, Univ Lyon, 69373 Lyon, France. ^4^Department of Clinical Analysis, Federal University of Parana, 80210-170 Curitiba, PR, Brazil. ^5^Laboratory of Applied Organic Chemistry, Synthesis of Biomolecules and Molecular Modelling Group, Badji-Mokhtar-Annaba University, Box 12, 23000 Annaba, Algeria. ^6^Département Sciences de la matière, Faculté des Sciences exactes et Sciences de la nature et de la vie, Université Larbi Ben M’hidi, Oum El Bouaghi, Algeria. ^7^Research Unit for Chemistry of the Environment and Molecular Structural, University of Constantine 1, Constantine, Algeria. ^8^Small Molecules for Biological Targets Team, Centre de recherche en cancérologie de Lyon, Centre Léon Bérard, CNRS 5286, INSERM 1052, Université Claude Bernard Lyon 1, Univ Lyon, Lyon, 69373, France. ^9^Institut für Pharmazeutische und Medizinische Chemie, PharmaCampus, Westfälische Wilhelms-Universität Münster, Corrensstr. 48, 48149, Münster, Germany. ^10^Department of Physics, Federal Technological University of Paraná, 80230-901 Curitiba, Parana, Brazil.

**^#^** Both Ph.D. students (Diogo Henrique Kita, and Nathalie Guragossian) and senior investigators (Glaucio Valdameri and Marc Le Borgne) contributed equally to this work. Correspondence and requests for materials should be addressed to Glaucio Valdameri email: [gvaldameri@ufpr.br](mailto:gvaldameri@ufpr.br) and Marc Le Borgne [marc.le-borgne@univ-lyon1.fr](mailto:marc.le-borgne@univ-lyon1.fr)).

**Table of contents**

Synthetic pathway of indeno[1,2-*b*]indole-9,10-diones derivatives **5** and **6** S3

NOE experiments part I S4

X-ray data S4

NOE experiments part II S5

Synthetic procedures and data S7

NMR and HR-MS spectra of tested compounds S23

Biology experiments S53

References S58

**Synthetic pathway of indeno[1,2-*b*]indole-9,10-diones derivatives 5 and 6**

**Figure S1. Synthetic route for the preparation of indeno[1,2-*b*]indole-9,10-diones derivatives 5 and 6.** Reagents and conditions: (a) R_5_NH_2_, toluene, reflux, 6 h; (b) MeOH, rt, 20 to 24 h; (c) (Et_2_N)_2_SO (TETA), DMF, AcOH, rt, 20 h; (d) 10% Pd-C, Ph_2_O, reflux, 6 h.

**Figure S2.** Structures of the used cyclohexanediones **1,** enaminones **2** and ninhydrins **3**.

**NOE experiments part I**

The most significant observed correlations for **5f**, **5g**, **5h** and **5i** are summarized in Fig. S3 as blue arrows. For **5f**, **5h** and **5i**, the aromatic proton H-4 showed correlations with the methylene protons of the phenethyl group (CH_2_N and CH_2_Ph) confirming the position 1 for the OH group**.** However, no correlation has been observed between the indenoindole aromatic protons and the methylene protons of the phenethyl group of the regioisomer **5g**, attesting the OH group at position 4.

**Figure S3.** Significant NOE interactions observed in the NOESY spectrum of 1 or 4-hydroxylated compounds **5f**, **5g**, **5h** and **5i**.

**X-ray Data**


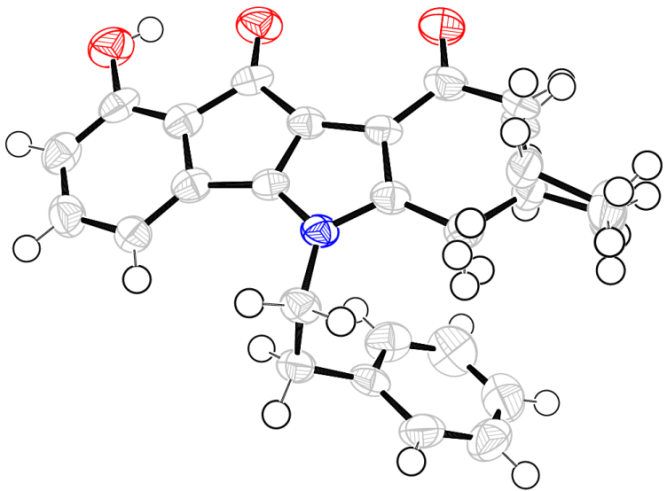


**Figure S4.** View of the crystal structure of **5f**.

Stick orange single crystal was obtained by slow evaporation from dichloromethane solution. The crystal was coated with Paratone oil and mounted on loops for data collection. X-ray data were collected with a Bruker D8 VENTURE CCD area detector diffractometer with a multilayer monochromator Mo-Kα radiation source (0.71073 Å) at 150 K. The reported structure was solved by direct methods with SIR2002 [S1] to locate all the non-H atoms which was refined anisotropically with SHELXL97 [S2] using full-matrix least-squares on F^2^ procedure from within the WinGX [S3] suite of software used to prepare material for publication.

All the H atoms were placed in the calculated positions and constrained to ride on their parent atoms. Drawing of molecule was produced with the program ORTEP-3 (Fig. S4) [S3].

Crystallographic data for the structural analysis of compound **5f** have been deposited with the Cambridge Crystallographic Data Center, CCDC 1914132. Copies of this information may be obtained free of charge from the Director, CCDC, 12 Union Road, Cambridge, CB2 1EZ, UK, by quoting the publication citation and the deposit numbers. [Fax: (int. code) +44 1223 336 033, E-mail: [deposit@ccdc.com.ac.uk](mailto:deposit@ccdc.com.ac.uk), <http://www.ccdc.cam.ac.uk>.].

**NOE experiments part II**

The most significant observed NOESY correlations for the *O*-prenylated compounds that allowed the assignment are given in Figure S5. Compounds **5j** and **5l** substituted at the position 1 are characterized both by the interactions between the aromatic proton H-4 and the methylene protons of the phenethyl group (CH_2_N and CH_2_Ph) and between the aromatic proton H-2 and the methylene protons of the prenyl group. We also noted the correlation between the methylene protons of CH_2_Ph and the aromatic proton H-6’.

For compounds **5k**, **5m** and **5n** substituted at the position 4, no correlation was observed between the methylene protons of the phenethyl group and the aromatic protons of the indenoindole’s ring A. The only aromatic proton that showed a correlation with CH_2_Ph is the proton at the position 6' of the phenyl group. On the other hand; we observed a correlation between the aromatic proton H-3 and the methylene protons of the prenyl group.

**Figure S5.** Significant NOE interactions observed in the NOESY spectrum of 1-substituted compounds **5j** and **5l** and 4-substituted compounds **5k**, **5m** and **5n.**

**Synthetic procedures and data**

**General considerations.** All of the reagents were purchased from Sigma-Aldrich and ThermoFisher Scientific. Melting points were determined on an Electrothermal 9200 capillary apparatus. The IR spectra were recorded on a PerkinElmer Spectrum Two IR spectrometer. The 1H and 13C NMR spectra were recorded at 400 MHz on a Bruker DRX 400 spectrometer. Chemical shifts are expressed in ppm (δ) downfield from internal tetramethylsilane, and coupling constants *J* are reported in hertz (Hz). The following abbreviations are used: s, singlet; bs, broad singlet; d, doublet; t, triplet; bt, broad triplet; dd, doubled doublet; dt, doubled triplet; q, quartet; qui, quintuplet; m, multiplet; Cquat, quaternary carbons. The mass spectra were performed by direct ionization (EI or CI) on a ThermoFinnigan MAT 95 XL apparatus. Chromatographic separations were performed on silica gel columns by column chromatography (Kieselgel 300−400 mesh). All reactions were monitored by TLC on GF254 plates that were visualized under a UV lamp (254 nm). Evaporation of solvent was performed in vacuum with rotating evaporator. The purity of the final compounds (greater than 95%) was determined by uHPLC/MS on an Agilent 1290 system using a Agilent 1290 Infinity ZORBAX Eclipse Plus C18 column (2.1 mm × 50 mm, 1.8 μm particle size) with a gradient mobile phase of H_2_O/CH_3_CN (90:10, v/v) with 0.1% of formic acid to H_2_O/CH_3_CN (10:90, v/v) with 0.1% of formic acid at a flow rate of 0.5 mL/min, with UV monitoring at the wavelength of 254 nm with a run time of 10 min.

**General procedure for synthesis of compounds 2.** Equimolar amounts (6.18 mmol) of primary amine and cyclohexane-1,3-dione **1** were dissolved in 35 mL of toluene. The mixture was refluxed in Dean-Stark trap for 5 h. The solvent was then evaporated under *vacuum* and the residual solid treated with ethyl acetate to obtain a yellow powder after filtration and drying. Purification of the filtrate by silica gel column chromatography afforded a second portion of the enaminone.

***3-((2-Methoxyphenethyl)amino)-5-methylcyclohex-2-en-1-one (2a):*** pale yellow solid; yield 73%; mp 111 °C; IR (ν, cm^-1^): 3268 (NH), 1537 (C=O); ^1^H NMR (CDCl_3_, 400 MHz): δ 7.23 (td, *J* = 7.5, 1.8 Hz, 1H, Harom-4’), 7.11 (dd, *J* = 7.4, 1.8 Hz, 1H, Harom-6’), 6.91 (td, *J* = 7.4, 1.1 Hz, 1H, Harom-5’), 6.87 (d, *J* = 7.5, 1.0 Hz, 1H, Harom-3’), 5.21 (bs, 1 H, NH), 5.15 (s, 1H, H-2), 3.84 (s, 3H, OCH_3_), 3.29 (q, *J* = 6.4 Hz, 2H, NCH_2_), 2.89 (t, *J* = 6.8 Hz, 2H, CH_2_Ph), 2.35 (dd, *J* = 17.5, 3.8 Hz, 1H, He of CH_2_), 2.18-2.05 (m, 3H, Ha and He of CH_2_ and H-5), 1.98 (dd, *J* = 16.2, 11.0 Hz, 1H, Ha of CH_2_), 1.02 (d, *J* = 6.1 Hz, 3H, CH_3_); ^13^C NMR + DEPT (CDCl_3_, 100 MHz): δ 197.00 (C=O), 163.98 (Cquat), 157.36 (Cquat), 130.57 (CH), 128.18 (CH), 126.90 (Cquat), 120.92 (CH), 110.51 (CH), 96.22 (CH), 55.33 (OCH_3_), 44.68 (CH_2_), 43.38 (CH_2_), 38.01 (CH_2_), 29.35 (CH), 29.32 (CH_2_), 21.07 (CH_3_); HRMS calculated for C_16_H_22_NO_2_ ­[M+H]^+^ 260.1645, found 260.1644.

***6-Benzyl-3-(phenethylamino)cyclohex-2-en-1-one (2b)*:** pale yellow solid; yield 68%; mp 118 °C; IR (ν, cm^-1^): 3266 (NH), 1546 (C=O); ^1^H NMR (CDCl_3_, 400 MHz): δ 7.27-7.15 (m, 5H, Harom), 7.13-7.09 (m, 5H, Harom), 5.14 (s, 1H, H-2), 4.45 (bs, 1H, NH), 3.35 (dd, *J* = 13.1, 3.2 Hz, 1H, Ha of CH_2_Ph), 3.29 (dt, *J* = 7.6, 7.0 Hz, 2H, CH_2_NH), 2.81 (t, *J* = 7.0 Hz, 2H, CH_2_-Ph), 2.43 (dd, *J* = 13.1, 10.6 Hz, 1H, Hb of CH_2_Ph), 2.40-2.35 (m, 1H, Ha of CH_2_-4), 2.23-2.09 (m, 2H, Hb of CH_2_-4 and CH_2_-6), 1.78 (dq, *J* = 13.2, 5.0 Hz, 1H, Ha of CH_2_-5), 1.56-1.47 (m, 1H, Hb of CH_2_-5); ^13^C NMR + DEPT (CDCl_3_, 100 MHz): δ 198.09 (C=O), 163.16 (Cquat), 140.73 (Cquat), 138.14 (Cquat), 129.24 (2CH), 128.81 (2CH), 128.65 (2CH), 128.32 (2CH), 126.81 (CH), 125.92 (CH), 96.81 (CH), 46.62 (CH), 43.82 (CH_2_), 36.20 (CH_2_), 34.42 (CH_2_), 28.56 (CH_2_), 25.93 (CH_2_); HRMS calculated for C_21_H_24_NO [M+H]^+^ 306.1852, found 306.1865.

***3-((2-(5-Methoxy-1H-indol-3-yl)ethyl)amino)cyclohex-2-en-1-one (2c)*:** pale yellow solid; yield 82%; mp 99 °C; IR (ν, cm^-1^): 3383 (NH), 3242 (NH), 1590 (C=O); ^1^H NMR (CDCl_3_, 400 MHz): δ 10.74 (s, 1H, NH), 7.27 (d, *J* = 8.9 Hz, 1H, Harom-7’), 7.19 (s, 1H, Harom-2’), 7.14 (bs, 1H, NH), 7.03 (s, *J* = 7.4, 1.1 Hz, 1H, Harom-4’), 6.76 (d, *J* = 8.7 Hz, 1H, Harom-6’), 4.95 (s, 1H, H-2), 3.81 (s, 3H, OCH_3_), 3.29 (q, *J* = 5.5 Hz, 2H, NCH_2_), 2.93 (t, *J* = 7.1 Hz, 2H, CH_2_Ph), 2.35 (t, *J* = 5.7 Hz, 2H, CH_2_-4), 2.13-2.10 (m, 2H, CH_2_-6), 1.84 (bt, *J* = 5.6 Hz, 2H, CH_2_-5); ^13^C NMR + DEPT (CDCl_3_, 100 MHz): δ 195.19 (C=O), 165.22 (Cquat), 153.97 (Cquat), 132.28 (Cquat), 128.37 (Cquat), 124.50 (CH), 113.03 (CH), 112.19 (Cquat), 112.06 (CH), 100.84 (CH), 95.66 (CH), 56.27 (OCH_3_), 43.75 (CH_2_), 37.50 (CH_2_), 29.53 (CH_2_), 24.64 (CH_2_), 22.70 (CH_2_); HRMS calculated for C_17_H_21_N_2_O_2_ [M+H]^+^ 285.1598, found 285.1600.

**General procedure for the synthesis of compounds 4.** A mixture of equimolar amounts (4.62 mmol) of enaminone **2** and ninhydrin **3** in 12 mL of MeOH was stirred at room temperature for 22 h. The precipitate was filtrated and washed with MeOH to obtain **4** as a mixture of two diastereoisomers. The filtrate was then evaporated and treated with diethylether to obtain an additional portion of compound **4**.

***4b,9b-Dihydroxy-5-(2-methoxyphenethyl)-7-methyl-4b,5,6,7,8,9b-hexahydroindeno[1,2-b]indole-9,10-dione (4a):*** pale yellow solid; yield 89%; mp 230 °C; IR (ν, cm^-1^): 3405 (OH), 3272 (OH), 1714 (C=O), 1599 (C=O); ^1^H NMR (DMSO-*d_6_*, 400 MHz): δ 8.01 (t, *J* = 7.5 Hz, 2H, Harom), 7.83-7.78 (m, 2H, Harom), 7.71 (d, *J* = 7.4 Hz, 1H, Harom), 7.70 (d, *J* = 6.2 Hz, 1H, Harom), 7.60-7.56 (m, 2H, Harom), 7.26 (d, *J* = 7.3 Hz, 2H, Harom), 7.23 (t, *J* = 7.7 Hz, 2H, Harom), 7.02 (d, *J* = 7.8 Hz, 1H, Harom), 7.00 (d, *J* = 8.0 Hz, 1H, Harom), 6.91 (t, *J* = 7.3 Hz, 2H, Harom), 6.80 (s, 1H, OH-4a), 6.79 (s, 1H, OH-4a), 5.74 (s, 1H, OH-9b), 5.71 (s, 1H, OH-9b), 3.86 (s, 3H, OCH_3_), 3.83 (s, 3H, OCH_3_), 3.82-3.77 (m, 2H, NCH_2_), 3.76-3.66 (m, 2H, NCH_2_), 3.01-2.96 (m, 2H, CH_2_Ph), 2.94-2.81 (m, 2H, CH_2_Ph), 2.45-2.42 (m, 2H, 2Ha of CH_2_-8), 2.07-1.78 (m, 8H, 2He of CH_2_-8 + 2H of CH-7 + 4H of CH_2_-6), 0.93 (d, *J* = 5.8 Hz, 3H, CH_3_), 0.90 (d, *J* = 5.3 Hz, 3H, CH_3_); ^13^C NMR + DEPT (DMSO-*d_6_*, 100 MHz): δ 198.25 (C=O), 197.82 (C=O), 188.95 (C=O), 188.76 (C=O), 165.48 (Cquat), 165.37 (Cquat), 157.75 (Cquat), 157.65 (Cquat), 148.94 (Cquat), 148.67 (Cquat), 136.02 (CH), 135.90 (CH), 135.35 (Cquat), 135.18 (Cquat), 131.26 (CH), 131.05 (CH), 130.67 (CH), 130.65 (CH), 128.55 (CH), 128.51 (CH), 127.03 (Cquat), 126.87 (Cquat), 125.29 (CH), 125.21 (CH), 123.68 (CH), 123.53 (CH), 120.96 (CH), 120.92 (CH), 111.18 (CH), 111.16 (CH), 104.77 (Cquat), 104.74 (Cquat), 96.35 (Cquat), 96.19 (Cquat), 84.03 (Cquat), 83.75 (Cquat), 55.85 (OCH_3_), 55.81 (OCH_3_), 45.95 (CH_2_), 45.76 (CH_2_), 42.18 (CH_2_), 42.12 (CH_2_), 32.53 (2CH_2_), 30.58 (CH_2_), 30.47 (CH_2_), 29.76 (CH), 29.26 (CH), 21.39 (CH_3_), 21.23 (CH_3_); HRMS calculated for C_25_H_26_NO_5_ [M+H]^+^ 420.1805, found 420.1793.

***8-Benzyl-4b,9b-dihydroxy-5-(2-phenylethyl)-4b,5,6,7,8,9b-hexahydroindeno[1,2-b]indole-9,10-dione (4b):*** white solid; yield 58%; mp 117 °C; IR (ν, cm^-1^): 3050 (OH), 2980 (OH), 1606 (C=O), 1538 (C=O); ^1^H NMR (DMSO-*d_6_*, 400MHz): δ 8.11 (d, *J* = 7.7 Hz, 2H, Harom), 7.80 (t, *J* = 7.5 Hz, 2H, Harom), 7.74 (d, *J* = 7.6 Hz, 1H, Harom), 7.71 (d, *J* = 7.6 Hz, 1H, Harom), 7.62-7.56 (m, 2H, Harom), 7.50-7.21 (m, 20H, Harom), 6.98 (s, 1H, OH-4a), 6.97 (s, 1H, OH-4a), 5.89 (s, 1H, OH-9b), 5.88 (s, 1H, OH-9b), 4.13-4.02 (m, 2H, NCH_2_), 3.84-3.75 (m, 2H, NCH_2_), 3.21 (dd, *J* = 13.2, 3.2 Hz, 2H, Ha of CH_2_-Ph), 3.15-2.97 (m, 4H, CH_2_-Ph), 2.40-2.20 (m, 4H, Hb of CH_2_-Ph and Ha of CH_2_-6), 2.19-2.06 (m, 4H, Hb of CH_2_-6 and H-8), 1.75-1.64 (m, 2H, Hb of CH_2_-7), 1.48-1.28 (m, 2H, Hb of CH_2_-7); ^13^C NMR + DEPT (DMSO-*d_6_*, 100 MHz): δ 198.14 (C=O), 197.91 (C=O), 190.26 (C=O), 190.21 (C=O), 165.20 (Cquat), 165.14 (Cquat), 148.76 (Cquat), 148.63 (Cquat), 141.17 (Cquat), 141.10 (Cquat), 139.51 (Cquat), 139.42 (Cquat), 136.04 (CH), 136.02 (CH), 135.31 (Cquat), 135.23 (Cquat), 130.70 (2CH), 129.62 (2CH), 129.49 (4CH), 129.41 (2CH), 128.96 (2CH), 128.94 (2CH), 128.62 (2CH), 128.57 (2CH), 126.87 (CH), 126.85 (CH), 126.18 (2CH), 125.26 (CH), 125.21 (CH), 123.69 (CH), 123.61 (CH), 104.80 (Cquat), 104.72 (Cquat), 96.28 (Cquat), 96.22 (Cquat), 84.18 (Cquat), 84.07 (Cquat), 47.17 (CH), 46.85 (CH), 43.91 (CH_2_), 43.85 (CH_2_), 37.37 (CH_2_), 37.31 (CH_2_), 35.88 (CH_2_), 35.67 (CH_2_), 25.83 (CH_2_), 25.75 (CH_2_), 21.50 (CH_2_), 21.20 (CH_2_); HRMS calculated for C_30_H_28_NO_4_ [M+H]^+^ 466.2013, found 466.2025.

***4b,9b-Dihydroxy-5-(2-(5-methoxy-1H-indol-3-yl)ethyl)-4b,5,6,7,8,9b-hexahydroindeno[1,2-b]indole-9,10-dione (4c):*** beige solid; yield 95%; mp 225 °C; IR (ν, cm^-1^): 3283 (NH and OH), 1715 (C=O), 1579 (C=O); ^1^H NMR (DMSO-*d*_6_, 400 MHz): δ 10.78 (s, 1H, NH), 7.98 (d, *J* = 7.8 Hz, 1H, Harom), 7.81 (td, *J* = 8.0, 1.1 Hz, 1H, Harom), 7.74 (d, *J* = 7.7 Hz, 1H, Harom), 7.61 (td, *J* = 7.8, 0.7 Hz, 1H, Harom), 7.30 (d, *J* = 8.7 Hz, 1H, Harom), 7.26 (d, *J* = 2.3 Hz, 1H, Harom), 7.15 (d, *J* = 2.3 Hz, 1H, Harom), 6.94 (s, 1H, OH), 6.78 (dd, *J* = 8.8, 2.3 Hz, 1H, Harom), 5.77 (s, 1H, OH), 4.14-4.01 (m, 1H, Ha of NCH_2_), 3.80 (s, 3H, OCH_3_), 3.80-3.73 (m, 1H, Hb of NCH_2_), 3.21-3.13 (m, 1H, Ha of CH_2_Ph), 3.06-3.00 (m, 1H, Hb of CH_2_Ph), 2.36 (t, *J* = 6.0 Hz, 2H, CH_2_-6), 2.12-2.08 (m, 2H, CH_2_-8), 1.78-1.72 (m, 1H, Ha of CH_2_-7), 1.66-1.59 (m, 1H, Hb of CH_2_-7); ^13^C NMR + DEPT (DMSO-*d*_6_, 100 MHz): δ 198.55 (C=O), 189.61 (C=O), 166.14 (Cquat), 154.05 (Cquat), 149.27 (Cquat), 136.48 (CH), 135.75 (Cquat), 132.25 (Cquat), 131.15 (CH), 128.32 (Cquat), 125.53 (CH), 124.93 (CH), 124.12 (CH), 113.06 (CH), 112.12 (CH), 111.90 (Cquat), 105.57 (Cquat), 101.05 (CH), 96.61 (Cquat), 84.57 (Cquat), 56.24 (OCH_3_), 43.57 (NCH_2_), 37.85 (CH_2_), 27.75 (CH_2_), 23.30 (CH_2_), 22.29 (CH_2_); HRMS calculated for C_26_H_25_N_2_O_5_ [M+H]^+^ 445.1758, found 445.1755.

***3,4b,9b-Trihydroxy-5-(3-methoxyphenethyl)-4b,5,6,7,8,9b-hexahydroindeno[1,2-b]indole-9,10-dione (4d):*** beige solid; yield 61%; mp 214 °C; IR (ν, cm^-1^): 3391 (OH), 1703 (C=O), 1593; ^1^H NMR (DMSO-*d*_6_, 400 MHz): δ 10.74 (bs, 1H, OH-3), 7.56 (d, *J* = 8.4 Hz, 1H, H-1), 7.26 (t, *J* = 7.8 Hz, 1H, H-5’), 7.21 (d, *J* = 1.9 Hz, 1H, H-4), 6.97 (dd, *J* = 8.5 Hz, 2.0 Hz, 1H, H-2), 6.90-6.86 (m, 2H, H-6’and H-2’), 6.83 (dd, *J* = 8.0 Hz, 2.2 Hz, 1H, H-4’), 6.78 (bs, 1H, OH-4a), 5.57 (s, 1H, OH-9b), 3.75 (s, 3H, OCH_3_), 3.85-3.61 (m, 2H, NCH_2_), 3.01-2.83 (m, 2H, CH_2_Ph), 2.30-2.24 (m, 2H, CH_2_-8), 2.02 (t, *J* = 5.8 Hz, 2H, CH_2_-6), 1.76-1.60 (m, 2H, CH_2_-7); ^13^C NMR + DEPT (DMSO-*d*_6_, 100 MHz): δ 195.96 (C=O), 188.88 (C=O), 165.11 (Cquat), 164.22 (Cquat), 159.43 (Cquat), 151.40 (Cquat), 140.51 (Cquat), 129.61 (CH), 126.62 (Cquat), 125.60 (CH), 121.14 (CH), 118.71 (CH), 114.56 (CH), 111.99 (CH), 109.64 (CH), 105.39 (Cquat), 95.26 (Cquat), 83.73 (Cquat), 55.04 (OCH_3_), 43.42 (CH_2_), 36.86 (2CH_2_), 22.26 (CH_2_), 21.41 (CH_2_); HRMS calculated for C_24_H_24_NO_6_[M+H]^+^ 422.1598, found 422.1584.

***4b,9b-Dihydroxy-2,3-dimethoxy-7-methyl-5-phenethyl-4b,5,6,7,8,9b-hexahydroindeno[1,2-b]indole-9,10-dione (4e):*** beige solid; yield 55%; mp 123 °C; IR (ν, cm^-1^): 3300 (OH), 1699 (C=O), 1648 (C=O); ^1^H NMR (DMSO-*d*_6_, 400 MHz): δ 7.40 (s, 2H, H-1), 7.35-7.10 (m, 10H, Harom), 7.11 (s, 1H, H-4), 7.10 (s, 1H, H-4), 6.76 (bs, 2H, OH), 5.66 (bs, 2H, OH), 4.05-3.94 (m, 2H, NCH_2_), 3.92 (s, 6H, OCH_3_), 3.83 (s, 6H, OCH_3_), 3.80-3.70 (m, 2H, NCH_2_), 3.05-2.06 (m, 4H, CH_2_Ph), 2.31 (d, *J* = 13.9 Hz, 1H), 2.12-2.10 (m, 1H), 2.08-1.94 (m, 2H), 1.91-1.66 (m, 6H), 0.88 (d, *J* = 5.4 Hz, 3H, CH_3_), 0.83 (d, *J* = 6.0 Hz, 3H, CH_3_); ^13^C NMR + DEPT (DMSO-*d*_6_, 100 MHz): δ 196.45 (C=O), 196.04 (C=O), 188.65 (C=O), 188.46 (C=O), 164.63 (2Cquat), 155.58 (Cquat), 155.49 (Cquat), 151.09 (Cquat), 151.07 (Cquat), 143.31 (Cquat), 142.98 (Cquat), 139.10 (2Cquat), 129.12 (3CH), 128.92 (CH), 128.46 (3CH), 128.32 (CH), 127.92 (Cquat), 127.68 (Cquat), 126.33 (2CH), 105.78 (CH), 105.73 (CH), 104.89 (Cquat), 104.86 (Cquat), 103.64 (CH), 103.59 (CH), 95.80 (Cquat), 95.57 (Cquat), 83.37 (Cquat), 83.05 (Cquat), 56.28 (OCH_3_), 56.24 (OCH_3_), 55.74 (2OCH_3_), 45.37 (NCH_2_), 45.21 (NCH_2_), 43.34 (CH_2_), 43.30 (CH_2_), 36.92 (2CH_2_), 30.12 (CH_2_), 29.94 (CH_2_), 29.31 (CH), 28.86 (CH), 20.76 (2CH_3_); HRMS calculated for C_26_H_27_NNaO_6_[M+Na]^+^ 472.1731, found 472.1713.

**General procedure for the synthesis of compounds 5.** To a mixture of diastereoisomers **4** (8.51 mmol, 1 eq) in 26 mL of DMF, was added TETA (2.5 eq, 21 mmol) in presence of acetic acid (5.25 mL). The mixture was stirred at room temperature for 24 h. The solution was then poured in crushed ice and water (300 mL), and the mixture was stirred for 1 h. The precipitate was filtered, washed with water and dried to get a first quantity of **5**. The filtrate was extracted with diethylether, dried over anhydrous Na_2_SO_4_ and evaporated in vacuum. The residue was purified by silica gel column chromatography using CH_2_Cl_2_/methanol/ (9.5:1, v/v) as the eluent to afford a second part of **5**.

***5-(2-Methoxyphenethyl)-7-methyl-5,6,7,8-tetrahydroindeno[1,2-b]indole-9,10-dione (5a):*** orange solid; yield 89%; mp 204 °C; IR (ν, cm^-1^): 1664 (C=O), 1603 (C=O); ^1^H NMR (CDCl_3_, 400 MHz): δ 7.41 (d, *J* = 6.7 Hz, 1H, Harom), 7.23-7.17 (m, 2H, Harom), 7.09 (dd, *J* = 7.6, 7.2 Hz, 1H, Harom), 6.99 (d, *J* = 7.2 Hz, 1H, Harom), 6.91 (dd, *J* = 7.5, 1.8 Hz, 1H, Harom), 6.82 (d, *J* = 7.5 Hz, 1H, Harom), 6.81 (t, *J* = 6.7 Hz, 1H, Harom), 4.17-4.10 (m, 2H, NCH_2_), 3.79 (s, 3H, OCH_3_), 3.10-3.04 (m, 2H, CH_2_Ph), 2.41 (dd, *J* = 22.4, 9.7 Hz, 1H, Ha of CH_2_-8), 2.33 (dd, *J* = 16.0, 3.9 Hz, 1H, Ha of CH_2_-6), 2.11-1.97 (m, 2H, H-7 + He of CH_2_-8), 1.90 (dd, *J* = 16.0, 10.5 Hz, 1H, He of CH_2_-6), 0.99 (d, *J* = 6.0 Hz, 3H, CH_3_); ^13^C NMR + DEPT (CDCl_3_, 100 MHz): δ 192.05 (C=O), 184.34 (C=O), 157.49 (Cquat), 152.89 (Cquat), 150.25 (Cquat), 138.80 (Cquat), 135.03 (Cquat), 132.26 (CH), 130.84 (CH), 128.87 (CH), 128.20 (CH), 124.82 (Cquat), 123.66 (CH), 120.89 (CH), 119.66 (Cquat), 117.07 (CH), 116.91 (Cquat), 110.23 (CH), 55.29 (OCH_3_), 46.15 (CH_2_), 45.78 (CH_2_), 31.91 (CH_2_), 30.95 (CH), 29.62 (CH_2_), 21.14 (CH_3_); HRMS calculated for C_25_H_24_NO_3_ [M+H]^+^ 386.1751, found 386.1748.

***8-Benzyl-5-phenethyl-5,6,7,8-tetrahydroindeno[1,2-b]indole-9,10-dione (5b):*** beige solid; yield 85%; mp 84 °C; IR (ν, cm^-1^): 1702 (C=O), 1664 (C=O); ^1^H NMR (CDCl_3_, 400 MHz): δ 7.45 (d, *J* = 7.0 Hz, 1H, Harom), 7.28-7.10 (m, 10H, Harom), 6.98-6.96 (m, 2H, Harom), 6.95 (d, *J* = 7.2 Hz, 1H, Harom), 4.20-4.04 (m, 2H, NCH_2_), 3.39-3.26 (m, 1H, Ha of CH_2_Ph), 3.06 (bt, *J* = 7.2 Hz, 2H, CH_2_Ph), 2.48-2.36 (m, 2H, Hb of CH_2_Ph and Ha of CH_2_-6), 2.04-1.91 (m, 2H, Hb of CH_2_-6 and H-8), 1.82-1.75 (dq, *J* = 13.3, 4.4 Hz, 1H, Ha of CH_2_-7), 1.49-1.38 (m, 1H, Hb of CH_2_-7); ^13^C NMR + DEPT (CDCl_3_, 100 MHz): δ 193.42 (C=O), 184.31 (C=O), 152.43 (Cquat), 149.98 (Cquat), 140.17 (Cquat), 138.82 (Cquat), 136.72 (Cquat), 134.85 (Cquat), 132.35 (CH), 129.19 (2CH), 129.0 (CH), 128.99 (CH), 128.98 (2CH), 128.40 (CH), 128.35 (2CH), 127.46 (CH), 126.06 (CH), 123.90 (CH), 120.46 (Cquat), 116.97 (CH), 116.87 (Cquat), 48.13 (CH), 47.41 (CH_2_), 36.95 (CH_2_), 35.14 (CH_2_), 26.72 (CH_2_), 20.36 (CH_2_); HRMS calculated for C_30_H_26_NO_2_ [M+H]^+^ 432.1958, found 432.1959.

***5-(2-(5-Methoxy-1H-indol-3-yl)ethyl)-5,6,7,8-tetrahydroindeno[1,2-b]indole-9,10-dione (5c):*** orange solid; yield 67%; mp 279 °C; IR (ν, cm^-1^): 3345 (NH), 1696 (C=O), 1655 (C=O); ^1^H NMR (DMSO-*d*_6_, 400 MHz): δ 10.78 (s, 1H, NH), 7.31-7.26 (m, 2H, Harom), 7.22-7.15 (m, 3H, Harom), 7.09 (d, *J* = 2.4 Hz, 1H, Harom), 6.73 (d, *J* = 2.3 Hz, 1H, Harom), 6.68 (dd, *J* = 8.7, 2.4 Hz, 1H, Harom), 4.34 (t, *J* = 6.3 Hz, 2H, NCH_2_), 3.65 (s, 3H, OCH_3_), 3.16 (t, *J* = 6.2 Hz, 2H, CH_2_Ph), 2.27 (t, *J* = 6.0 Hz, 2H, CH_2_-6), 2.19-2.15 (m, 2H, CH_2_-8), 1.67 (qui, *J* = 6.0 Hz, 2H, CH_2_-7); ^13^C NMR + DEPT (DMSO-*d*_6_, 100 MHz): δ 191.24 (C=O), 183.59 (C=O), 153.15 (Cquat), 152.52 (Cquat), 151.87 (Cquat), 138.01 (Cquat), 134.39 (Cquat), 132.77 (CH), 131.15 (Cquat), 128.25 (CH), 127.30 (Cquat), 124.31 (CH), 122.84 (CH), 118.68 (Cquat), 117.95 (CH), 116.51 (Cquat), 112.04 (CH), 111.56 (CH), 109.72 (Cquat), 98.94 (CH), 54.96 (OCH_3_), 47.00 (NCH_2_), 37.62 (CH_2_), 25.91 (CH_2_), 22.36 (CH_2_), 21.04 (CH_2_); HRMS calculated for C_26_H_22_N_2_NaO_3_ [M+Na]^+^ 433.1523, found 433.1527.

***3-Hydroxy-5-(3-methoxyphenethyl)-5,6,7,8-tetrahydroindeno[1,2-b]indole-9,10-dione (5d):*** orange solid; yield 68%; mp 222 °C; IR (ν, cm^-1^): 3283 (OH), 1696 (C=O), 1648 (C=O) cm^-1^; ^1^H NMR (DMSO-*d*_6_, 400 MHz): δ 10.30 (s, 1H, OH-3), 7.17 (dd, *J* = 8.3 Hz, *J* = 7.5Hz, 1H, H-5’), 7.13 (d, *J* = 7.9 Hz, 1H, H-1), 6.77 (dd, *J* = 7.3 Hz, *J* = 2.5 Hz, 1H, H-2), 6.71-6.64 (m, 3H, H-4, H-2 and H-6’), 6.43 (dd, *J* = 7.9 Hz, 2.0 Hz, 1H, H-4’), 4.27 (t, *J* = 6.7 Hz, 2H, NCH_2_), 3.66 (s, 3H, OCH_3_), 2.99 (t, *J* = 6.7 Hz, 2H, CH_2_Ph), 2.32 (t, *J* = 6.1 Hz, 2H, CH_2_-6), 2.22 (dd, *J* = 7.3 Hz, *J* = 5.3 Hz, CH_2_-8), 1.80 (qui, *J* = 6.1 Hz, 2H, CH_2_-7); ^13^C NMR + DEPT (DMSO-*d*_6_, 100 MHz): δ 191.29 (C=O), 183.40 (C=O), 161.94 (Cquat), 159.34 (Cquat), 151.14 (Cquat), 150.36 (Cquat), 138.91 (Cquat), 136.85 (Cquat), 129.58 (CH), 128.62 (Cquat), 124.91 (CH), 121.22 (CH), 119.87 (Cquat), 116.29 (Cquat), 114.49 (CH), 112.48 (CH), 111.72 (CH), 107.69 (CH), 54.90 (OCH_3_), 46.76 (CH_2_), 37.72 (CH_2_), 35.99 (CH_2_), 22.58 (CH_2_), 21.09 (CH_2_); HRMS calculated for C_24_H_21_NNaO_4_[M+Na]^+^ 410.1363, found 410.1362.

***2,3-Dimethoxy-7-methyl-5-phenethyl-5,6,7,8-tetrahydroindeno[1,2-b]indole-9,10-dione (5e):*** red solid; yield 74%; mp 235 °C; IR (ν, cm^-1^): 1697 (C=O), 1663 (C=O) cm^-1^; ^1^H NMR (DMSO-*d*_6_, 400 MHz): δ 7.26-7.21 (m, 3H, H-3’, H-4’ and H-5’), 7.05 (s, 1H, H-1), 7.00-6.97 (m, 2H, H-2’ and H-6’), 6.38 (s, 1H, H-4), 4.16-4.02 (m, 2H, NCH_2_), 3.86 (s, 3H, OCH_3_), 3.85 (s, 3H, OCH_3_), 3.06-3.03 (m, 2H, CH_2_Ph), 2.38 (dd, *J* = 22.8 Hz, 10.2 Hz, 1H, Ha of CH_2_-8), 2.13 (dd, *J* = 15.8 Hz, 3.7Hz, 1H, He of CH_2_-6), 2.03-1.94 (m, 2H, He of CH_2_-8 and H-7), 1.74 (dd, *J* = 16.0 Hz, 10.5 Hz, 1H, Ha of CH_2_-6), 0.94 (d, *J* = 5.9 Hz, 3H, CH_3_); ^13^C NMR + DEPT (DMSO-*d*_6_, 100 MHz): δ 192.31 (C=O), 184.36 (C=O), 152.22 (Cquat), 151.43 (Cquat), 149.21 (Cquat), 148.45 (Cquat), 136.83 (Cquat), 131.50 (Cquat), 129.08 (2CH), 129.05 (2CH), 128.90 (Cquat), 127.55 (CH), 119.16 (Cquat), 117.27 (Cquat), 109.09 (CH), 102.84 (CH), 56.56 (OCH_3_), 56.45 (OCH_3_), 47.50 (CH_2_), 46.16 (CH_2_), 37.04 (CH_2_), 30.99 (CH), 29.76 (CH_2_), 21.17 (CH_3_); HRMS calculated for C_26_H_25_NNaO_4_ [M+Na]^+^ 438.1676, found: 438.1667.

**General procedure for hydroxyl derivatives 5f, 5g, 5h and 5i**

Equimolar amounts of 4-hydroxyninhydrin **3b** (10.3 mmol, 1 eq) and the corresponding enaminone **2** (10.3 mmol, 1 eq) were dissolved in 20 mL of methanol. The reaction mixture was stirred at room temperature for 24 h. The precipitate was filtered and washed with methanol to get the corresponding trihydroxyindenoindolediones **4** and **4’** as a mixture of two regioisomers which could not be separated. The mixture of **4** and **4’** (2.4 mmol, 1 eq) was then dissolved in DMF and treated with TETA (6.16 mmol, 2.5 eq) in presence of acetic acid (1.13 mL) under stirring at room temperature during 24 h. A crushed ice was added and the mixture was left under stirring for 1 h. The precipitate was filtered and washed with water and then dried. The filtrate which still contains **5**, was extracted with dichloromethane, dried over Na_2_SO_4_, and evaporated. Finally, the residue was purified and regioisomers of **5** were separated by flash chromatography using acetone/ CH_2_Cl_2_ (1:9, v/v) as the eluent.

***1-Hydroxy-7-methyl-5-phenethyl-5,6,7,8-tetrahydroindeno[1,2-b]indole-9,10-dione (5f):*** obtained according to the general procedure from enaminone **2e**. Non-separable regioisomers **4** and **4’** were obtained with 78% yield. Orange solid; yield 68%; mp 246 °C; IR (ν, cm^-1^): 3380 (OH), 1658 (C=O), 1615 (C=O); ^1^H NMR (400 MHz, CDCl_3_): δ 8.74 (s, 1H, OH), 7.34-7.17 (m, 3H, Harom), 7.09 (dd, *J* = 7.08, 8.6 Hz, 1H, H-3), 7.04-6.94 (m, 2H, Harom), 6.65 (d, 1H, *J* = 8.6 Hz, H-2), 6.49 (d, 1H, *J* = 7.0 Hz, H-4), 4.28-4.01 (m, 2H, NCH_2_), 3.17-2.97 (m, 2H, CH_2_Ph), 2.52-2.34 (m, 1H, Ha of CH_2_-8), 2.15-1.94 (m, 3H, Ha of CH_2_-6, H-7 and He of CH_2_-8), 1.79-1.62 (m, 1H, He of CH_2_-6), 0.94 (d, *J* = 5.8 Hz, 3H, CH_3_); ^13^C NMR + DEPT (100 MHz, CDCl_3_): δ 192.11 (C=O), 188.33 (C=O), 156.57 (2Cquat), 151.68 (Cquat), 150.27 (Cquat), 136.77 (Cquat), 134.98 (CH), 134.41 (Cquat), 129.06 (2CH), 129.04 (2CH), 127.50 (CH), 119.74 (CH), 119.20 (Cquat), 117.02 (Cquat), 110.64 (CH), 47.70 (CH_2_), 46.06 (CH_2_), 36.84 (CH_2_), 30.99 (CH), 29.70 (CH_2_), 21.07 (CH_3_); HRMS calcd. for C_24_H_22_NO_3_[M+H]^+^ 372.1594, found 372.1588.

***4-Hydroxy-7-methyl-5-phenethyl-5,6,7,8-tetrahydroindeno[1,2-b]indole-9,10-dione (5g):*** obtained according to the general procedure from enaminone **2e**. Non-separable regioisomers **4** and **4’** were obtained with 78% yield. Orange solid; yield 18%; mp 283 °C; IR (ν, cm^-1^): 3028 (OH), 1693 (C=O), 1641 (C=O); ^1^H NMR (400 MHz, DMSO-*d*_6_): δ 10.66 (s, 1H, OH), 7.36-7.22 (m, 5H, Harom), 7.08 (dd, *J* = 8.4, 6.9 Hz, 1H, H-2), 6.95 (dd, *J* = 8.3, 1.0 Hz, 1H, H-3), 6.88 (dd, *J* = 6.9, 0.9 Hz, 1H, H-1), 4.57 (t, *J* = 7.4 Hz, 2H, NCH_2_), 3.05 (t, *J* = 7.3 Hz, 2H, CH_2_Ph), 2.76-2.55 (m, 1H, Ha of CH_2_-6), 2.34-2.25 (m, 1H, Hb of CH_2_-6), 2.20-1.99 (m, 3H, CH_2_-8 and H-7), 1.02 (d, *J* = 5.3 Hz, 3H, CH_3_); ^13^C NMR + DEPT (100 MHz, DMSO-*d*_6_): δ 190.95 (C=O), 183.52 (C=O), 153.51 (Cquat), 150.59 (Cquat), 148.14 (Cquat), 139.91 (Cquat), 137.87 (Cquat), 130.12 (CH), 129.06 (2CH), 128.39 (2CH), 126.63 (CH), 123.17 (CH), 118.53 (Cquat), 117.57 (Cquat), 116.18 (Cquat), 115.02 (CH), 47.75 (CH_2_), 45.88 (CH_2_), 37.50 (CH_2_), 30.38 (CH), 29.30 (CH_2_), 20.75 (CH_3_); HRMS calcd. for C_24_H_22_NO_3_[M+H]^+^ 372.1594, found 372.1589.

***1-Hydroxy-5-(2-methoxyphenethyl)-5,6,7,8-tetrahydroindeno[1,2-b]indole-9,10-dione (5h):*** obtained according to the general procedure from enaminone **2f**. Non-separable regioisomers **4** and **4’** were obtained with 64% yield. Separation of **5h** from its regiosiomer was incomplete. Orange solid; mp 173 °C; IR (ν, cm^-1^): 1685 (C=O), 1656 (C=O); ^1^H NMR (CDCl_3_, 400 MHz): δ 8.71 (s, 1H, OH), 7.21 (td, *J* = 7.8, 1.8 Hz, 1H, Harom), 7.06 (dd, *J* = 8.6, 7.1 Hz, 1H, Harom), 6.89 (dd, *J* = 7.8, 1.8 Hz, 1H, Harom), 6.83-6.79 (m, 2H, Harom), 6.61 (d, *J* = 8.7 Hz, 1H, Harom), 6.56 (d, *J* = 6.5 Hz, 1H, Harom), 4.11 (t, *J* = 6.9 Hz, 2H, NCH_2_), 3.78 (s, 3H, OCH_3_), 3.05 (t, *J* = 6.9 Hz, 2H, CH_2_Ph), 2.34 (dd, *J* = 7.2, 5.5 Hz, 2H, CH_2_-8), 2.26 (t, *J* = 6.1 Hz, 2H, CH_2_-6), 1.90 (qui, *J* = 6.3 Hz, 2H, CH_2_-7); ^13^C NMR + DEPT (CDCl_3_, 100 MHz): δ 192.32 (C=O), 188.28 (C=O), 157.46 (Cquat), 156.36 (Cquat), 152.05 (Cquat), 150.52 (Cquat), 134.85 (CH), 134.44 (Cquat), 130.76 (CH), 128.93 (CH), 124.76 (Cquat), 120.90 (CH), 119.52 (CH), 119.39 (Cquat), 119.19 (Cquat), 117.27 (Cquat), 110.72 (CH), 110.29 (CH), 55.32 (OCH_3_), 45.96 (CH_2_), 37.70 (CH_2_), 31.74 (CH_2_), 23.05 (CH_2_), 21.47 (CH_2_); HRMS calculated for C_24_H_22_NO_4_ [M+H]^+^ 388.1543, found 388.1547.

***1-Hydroxy-5-(2-methoxyphenethyl)-7-methyl-5,6,7,8-tetrahydroindeno[1,2-b]indole-9,10-dione (5i):*** obtained according to the general procedure from enaminone **2a**. Non-separable regioisomers **4** and **4’** were obtained with 36% yield. Separation of **5i** from its regiosiomer was incomplete. Orange solid; mp 198 °C; IR (ν, cm^-1^): 3328 (OH), 1676 (C=O), 1622 (C=O); ^1^H NMR (400 MHz, CDCl_3_): δ 8.71 (s, 1H, OH), 7.21 (dd, *J* = 7.8 Hz, 1.7 Hz, 1H, Harom), 7.08 (t, *J* = 7.6 Hz, 1H, Harom), 6.91 (dd, *J* = 7.6, 1.8 Hz, 1H, Harom), 6.84-6.80 (m, 2H, Harom), 6.64 (d, *J* = 8.5 Hz, 1H, Harom), 6.59 (d, *J* = 6.8 Hz, 1H, Harom), 4.14 (t, *J* = 6.8 Hz, 2H, NCH_2_), 3.79 (s, 3H, OCH_3_), 3.08 (t, *J* = 6.5 Hz, 2H, CH_2_Ph), 2.48 (dd, *J* = 22.5, 10.0 Hz, 1H, Ha of CH_2_-8), 2.35 (dd, *J* = 16.0, 3.7 Hz, 1H, He of CH_2_-6), 2.09-2.02 (m, 2H, H of CH_2_-7 and He of CH_2_-8), 1.91 (dd, *J* = 16.0 Hz, 9.7 Hz, 1H, Ha of CH_2_-6), 1.00 (d, *J* = 5.8 Hz, 3H, CH_3_); ^13^C NMR + DEPT (100 MHz, CDCl_3_): δ 192.05 (C=O), 188.29 (C=O), 157.51 (Cquat), 156.48 (Cquat), 152.22 (Cquat), 150.02 (Cquat), 134.85 (CH), 134.55 (Cquat), 130.85 (CH), 128.95 (CH), 124.80 (Cquat), 120.96 (CH), 119.64 (CH), 119.35 (Cquat), 119.22 (Cquat), 116.99 (Cquat), 110.65 (CH), 110.29 (CH), 58.50 (NCH_2_), 55.32 (OCH_3_), 46.01 (CH_2_), 31.80 (CH_2_), 31.03 (CH), 29.69 (CH_2_), 21.12 (CH_3_); HRMS calculated for C_25_H_24_NO_4_ [M+H]^+^ 402.1700, found 402.1708.

**General procedure for the synthesis of the *O*-prenyl derivatives 5j, 5k, 5l and 5m**. The 1- and 4-hydroxy derivatives were obtained from 4-hydroxyninhydrin **3b** and the corresponding enaminone **2** according to the procedure described above, as a mixture of regiosiomers **5** and **5’** which were not separated. To this mixture (0.871 mmol, 1 eq) in 7.5 mL of dry DMF was then added NaOH (1.05 mmol, 1.2 eq), and 3,3-dimethylallylbromide (2.61 mmol, 3 eq). The mixture was stirred at room temperature for 48 h and then poured into H_2_O and extracted with ethylacetate. The organic layers were then dried over Na_2_SO_4_, filtered, and evaporated in vacuum. The residue was purified by flash chromatography using cyclohexane/ethyl acetate (1:5, v/v) as the eluent.

***5-(2-Methoxyphenethyl)-1-(3-methylbut-2-enyloxy)-5,6,7,8-tetrahydroindeno[1,2-b]indole-9,10-dione (5j):*** prepared from enaminone **2f**. Non-separable 1- and 4-hydroxyregioisomers **5h** and **5h’** were obtained with 12% yield. Orange solid; yield 20%; mp 118 °C; IR (ν, cm^-1^): 1736 (C=O), 1699 (C=O); ^1^H NMR (CDCl_3_, 400 MHz): δ 7.23 (td, *J* = 7.8, 1.8 Hz, 1H, Harom), 7.17 (dd, *J* = 8.7, 7.1 Hz, 1H, Harom), 6.93 (dd, *J* = 7.6, 1.9 Hz, 1H, Harom), 6.85-6.82 (m, 2H, Harom), 6.74 (d, *J* = 8.7 Hz, 1H, Harom), 6.73 (d, *J* = 7.1 Hz, 1H, Harom), 5.50 (bt, *J* = 6.8 Hz, 1H, Me_2_C-CH), 4.70 (d, *J* = 6.6 Hz, 2H, OCH_2_), 4.16 (t, *J* = 6.9 Hz, 2H, NCH_2_), 3.81 (s, 3H, OCH_3_), 3.08 (t, *J* = 6.9 Hz, 2H, CH_2_Ph), 2.38-2.35 (m, 2H, CH_2_-8), 2.27 (t, *J* = 6.1 Hz, 2H, CH_2_-6), 1.90 (qui, *J* = 6.3 Hz, 2H, CH_2_-7), 1.76 (d, *J* = 0.9 Hz, 3H, Me_2_C=CH), 1.73 (s, 3H, Me_2_C=CH); ^13^C NMR + DEPT (CDCl_3_, 100 MHz): δ 192.11 (C=O), 183.10 (C=O), 157.50 (Cquat), 156.85 (Cquat), 150.41 (Cquat), 150.05 (Cquat), 137.76 (Cquat), 137.29 (Cquat), 134.01 (CH), 130.79 (CH), 128.82 (CH), 124.97 (Cquat), 123.45 (Cquat), 120.89 (CH), 120.36 (Cquat), 119.93 (CH), 117.45 (CH), 117.12 (Cquat), 110.78 (CH), 110.26 (CH), 66.70 (OCH_2_), 55.30 (OCH_3_), 45.67 (CH_2_), 37.89 (CH_2_), 31.84 (CH_2_), 25.78 (CH_3_), 23.07 (CH_2_), 21.53 (CH_2_), 18.38 (CH_3_); HRMS calculated for C_29_H_30_NO_4_ [M+H]^+^ 456.2169, found 456.2180.

***5-(2-Methoxyphenethyl)-4-(3-methylbut-2-enyloxy)-5,6,7,8-tetrahydroindeno[1,2-b]indole-9,10-dione (5k):*** prepared from enaminone **2f**. Non-separable 1- and 4-hydroxyregioisomers **5h** and **5h’** were obtained with 12% yield. Orange solid; yield 15%; mp 154 °C; IR (ν, cm^−1^): 1737 (C=O), 1701 (C=O); ^1^H NMR (CDCl_3_, 400 MHz): δ 7.20 (td, *J* = 7.3, 1.7 Hz, 1H, Harom), 7.18-7.13 (m, 1H, Harom), 7.10 (d, *J* = 7.2 Hz, 1H, Harom), 6.90 (dd, *J* = 8.2, 1.2 Hz, 1H, Harom), 6.86 (dd, *J* = 7.3, 1.2 Hz, 1H, Harom), 6.81-6.77 (m, 2H, Harom), 5.47 (bt, *J* = 6.8 Hz, 1H, Me_2_C-CH), 4.63 (d, *J* = 7.0 Hz, 2H, OCH_2_), 4.43 (t, *J* = 6.3 Hz, 2H, NCH_2_), 3.75 (s, 3H, OCH_3_), 2.99 (t, *J* = 6.2 Hz, 2H, CH_2_Ph), 2.30 (dd, *J* = 7.1, 5.5 Hz, 2H, CH_2_-8), 2.05 (t, *J* = 6.0 Hz, 2H, CH_2_-6), 1.79 (s, 3H, Me_2_C=CH), 1.75 (s, 3H, Me_2_C=CH), 1.74-1.60 (m, 2H, CH_2_-7); ^13^C NMR + DEPT (CDCl_3_, 100 MHz): δ 192.42 (C=O), 184.41 (C=O), 157.58 (Cquat), 153.18 (Cquat), 150.96 (Cquat), 149.13 (Cquat), 140.50 (Cquat), 138.97 (Cquat), 130.93 (CH), 130.11 (CH), 128.45 (CH), 125.93 (Cquat), 122.18 (Cquat), 120.65 (CH), 119.55 (Cquat), 118.83 (CH), 118.20 (CH), 116.98 (Cquat), 116.87 (CH), 109.95 (CH), 65.28 (OCH_2_), 55.27 (OCH_3_), 46.73 (CH_2_), 37.76 (CH_2_), 33.08 (CH_2_), 25.79 (CH_3_), 23.07 (CH_2_), 21.92 (CH_2_), 18.30 (CH_3_); HRMS calculated for C_29_H_30_NO_4_ [M+H]^+^ 456.2169, found 456.2166.

***5-(2-Methoxyphenethyl)-7-methyl-1-(3-methylbut-2-enyloxy)-5,6,7,8-tetrahydroindeno[1,2-b]indole-9,10-dione (5l):*** prepared from enaminone **2a**. Non-separable 1- and 4-hydroxyregioisomers **5i** and **5i’** were obtained with 15% yield. Orange solid; yield 30%; mp 135 °C; IR (ν, cm^-1^): 1792 (C=O), 1666 (C=O); ^1^H NMR (CDCl_3_, 400 MHz): δ 7.18 (td, *J* = 7.8, 1.8 Hz, 1H, Harom), 7.11 (td, *J* = 7.0, 1.1 Hz, 1H, Harom), 7.07 (d, *J* = 7.1 Hz, 1H, Harom), 6.88 (d, *J* = 8.1 Hz, 1H, Harom), 6.85 (dd, *J* = 7.3, 1.8 Hz, 1H, Harom), 6.79 (d, *J* = 8.5 Hz, 1H, Harom), 6.77 (t, *J* = 7.3 Hz, 1H, Harom), 5.45 (bt, *J* = 6.4 Hz, 1H, Me_2_C-CH), 4.62 (d, *J* = 6.8 Hz, 2H, OCH_2_), 4.46 (dt, *J* = 13.5, 6.0 Hz, 1H, NCH_2_), 4.36 (dt, *J* = 13.5, 6.5 Hz, 1H, NCH_2_), 3.74 (s, 3H, OCH_3_), 2.97 (t, *J* = 6.2 Hz, 2H, CH_2_Ph), 2.36 (dd, *J* = 15.7, 3.1 Hz, 1H, Ha of CH_2_-8), 2.15 (dd, *J* = 16.2, 4.2 Hz, 1H, Ha of CH_2_-6), 1.96 (dd, *J* = 15.7, 11.9 Hz, 1H, He of CH_2_-8), 1.77 (s, 3H, Me_2_C=CH), 1.73 (s, 3H, Me_2_C=CH), 1.76-1.57 (m, 2H, He of CH_2_-6 and H-7), 0.89 (d, *J* = 6.3 Hz, 3H, CH_3_); ^13^C NMR + DEPT (CDCl_3_, 100 MHz): δ 192.22 (C=O), 184.43 (C=O), 157.62 (Cquat), 153.34 (Cquat), 150.65 (Cquat), 149.13 (Cquat), 140.47 (Cquat), 138.97 (Cquat), 130.99 (CH), 130.11 (CH), 128.45 (CH), 125.95 (Cquat), 122.20 (Cquat), 120.67 (CH), 119.37 (Cquat), 118.83 (CH), 118.21 (CH), 116.86 (CH), 116.62 (Cquat), 109.90 (CH), 65.29 (OCH_2_), 55.24 (OCH_3_), 46.66 (CH_2_), 46.15 (CH_2_), 33.15 (CH_2_), 30.92 (CH), 30.13 (CH_2_), 25.79 (CH_3_), 21.13 (CH_3_), 18.30 (CH_3_); HRMS calculated for C_30_H_32_NO_4_ [M+H]^+^ 470.2326, found 470.2337.

***5-(2-Methoxyphenethyl)-7-methyl-4-(3-methylbut-2-enyloxy)-5,6,7,8-tetrahydroindeno[1,2-b]indole-9,10-dione (5m):*** prepared from enaminone **2a**. Non-separable 1- and 4-hydroxyregioisomers **5i** and **5i’** were obtained with 15% yield. Orange solid; yield 14%; mp 144 °C; IR (ν, cm^-1^): 1702 (C=O), 1664 (C=O); ^1^H NMR (CDCl_3_, 400 MHz): δ 7.20 (td, *J* = 7.9, 1.8 Hz, 1H, Harom), 7.14 (dd, *J* = 8.7, 7.1 Hz, 1H, Harom), 6.91 (dd, *J* = 7.6, 1.9 Hz, 1H, Harom), 6.81 (d, *J* = 8.8 Hz, 1H, Harom), 6.80 (t, *J* = 7.3 Hz, 1H, Harom), 6.70 (d, *J* = 8.7 Hz, 1H, Harom), 6.68 (d, *J* = 7.3 Hz, 1H, Harom), 5.47 (bt, *J* = 6.4 Hz, 1H, Me_2_C-CH), 4.67 (d, *J* = 6.5 Hz, 2H, OCH_2_), 4.13 (bd, *J* = 6.9 Hz, 2H, NCH_2_), 3.78 (s, 3H, OCH_3_), 3.06 (t, *J* = 6.9 Hz, 2H, CH_2_Ph), 2.40 (dd, *J* = 22.4, 10.1 Hz, 1H, He of CH_2_-8), 2.30 (dd, *J* = 15.6, 3.5 Hz, 1H, Ha of CH_2_-6), 2.06-1.98 (m, 2H, H-7 and Ha of CH_2_-8), 1.87 (dd, *J* = 15.7, 10.4 Hz, 1H, Ha of CH_2_-6), 1.74 (s, 3H, Me_2_C=CH), 1.71 (s, 3H, Me_2_C=CH), 0.96 (d, *J* = 5.4 Hz, 3H, CH_3_); ^13^C NMR + DEPT (CDCl_3_, 100 MHz): δ 191.93 (C=O), 183.16 (C=O), 157.51 (Cquat), 156.81 (Cquat), 150.57 (Cquat), 149.83 (Cquat), 137.77 (Cquat), 137.31 (Cquat), 134.06 (CH), 130.86 (CH), 128.80 (CH), 125.00 (Cquat), 123.37 (Cquat), 120.88 (CH), 120.16 (Cquat), 119.93 (CH), 117.35 (CH), 116.71 (Cquat), 110.80 (CH), 110.20 (CH), 66.66 (OCH_2_), 55.29 (OCH_3_), 46.28 (CH_2_), 45.61 (CH_2_), 31.84 (CH_2_), 30.96 (CH), 29.67 (CH_2_), 25.80 (CH_3_), 21.18 (CH_3_), 18.40 (CH_3_); HRMS calculated for C_30_H_32_NO_4_ [M+H]^+^ 470.2326, found 470.2327.

***7-Methyl-4-(3-methylbut-2-enyloxy)-5-phenethyl)-5,6,7,8-tetrahydroindeno[1,2-b]indole-9,10-dione (5n):*** prepared from hydroxyindenoindole **5g**. Orange solid; yield 56%; mp 191 °C; IR (ν, cm^-1^): 1699 (C=O), 1665 (C=O); ^1^H NMR (CDCl_3_, 400 MHz): δ 7.23-7.20 (m, 3H, Harom), 7.13 (dd, *J* = 7.0, 1.2 Hz, 1H, Harom), 7.10 (dd, *J* = 8.1, 7.0 Hz, 1H, Harom), 6.97-6.95 (m, 2H, Harom), 6.88 (dd, *J* = 8.1, 1.3 Hz, 1H, Harom), 5.46 (bt, *J* = 6.9 Hz, 1H, Me_2_C-CH), 4.61 (d, *J* = 6.8 Hz, 2H, OCH_2_), 4.46 (dt, *J* = 13.6, 6.1 Hz, 1H, NCH_2_), 4.36 (dt, *J* = 13.6, 6.5 Hz, 1H, NCH_2_), 2.95 (t, *J* = 6.4 Hz, 2H, CH_2_Ph), 2.36 (dd, *J* = 15.2, 2.3 Hz, 1H, He of CH_2_), 2.00-1.92 (m, 3H, H-7, Ha and He of CH_2_), 1.79 (s, 3H, Me_2_C=C), 1.75 (s, 3H, Me_2_C=C), 1.53 (dd, *J* = 16.0, 9.7 Hz, 1H, Ha of CH_2_), 0.87 (d, *J* = 6.1 Hz, 3H, CH_3_); ^13^C NMR + DEPT (CDCl_3_, 100 MHz): δ 192.21 (C=O), 184.37 (C=O), 152.79 (Cquat), 150.49 (Cquat), 149.10 (Cquat), 140.38 (Cquat), 139.18 (Cquat), 137.81 (Cquat), 130.19 (CH), 129.13 (2CH), 128.69 (2CH), 126.99 (CH), 121.99 (Cquat), 119.61 (Cquat), 118.68 (CH), 118.19 (CH), 116.88 (CH), 116.75 (Cquat), 65.30 (OCH_2_), 48.20 (CH_2_), 46.06 (CH_2_), 38.63 (CH_2_), 30.84 (CH), 30.22 (CH_2_), 25.83 (CH_3_), 21.03 (CH_3_), 18.28 (CH_3_); HRMS calculated for C_29_H_30_NO_3_ [M+H]^+^ 440.2220, found 440.2221.

**General procedure for the synthesis of compounds 6.** To a solution of compound **5** (2.4 mmol) in Ph_2_O (15 mL) was added 0.48 g of 10% Pd-C. The mixture was then heated to reflux for 6 h. After cooling, 25 mL of MeOH was added and the solution filtered on celite. Evaporation of the solvent left a residue which was purified by silica gel column chromatography using ethyl acetate (EtOAc)/cyclohexane (1:2, v/v) as the eluent.

***9-Hydroxy-5-(2-methoxyphenethyl)-7-methyl-5H-indeno[1,2-b]indol-10(5H)-one* *(6a):*** brown solid; yield 32%; mp 170 °C; IR (ν, cm^-1^): 3417 (OH), 1664 (C=O), 1603 (C=O); ^1^H NMR (CDCl_3_, 400 MHz): δ 7.29 (dd, 7.1, 1.8 Hz, 1H, Harom), 7.19 (td, *J* = 7.4, 1.8 Hz, 1H, Harom), 7.13 (td, *J* = 7.0, 1.4 Hz, 1H, Harom), 7.08 (td, *J* = 7.0, 1.0 Hz, 1H, Harom), 7.00 (dd, *J* = 7.7, 1.7 Hz, 1H, Harom), 6.98 (d, *J* = 7.2 Hz, 1H, Harom), 6.85-6.81 (m, 2H, Harom), 6.58 (s, 1H, Harom), 6.50 (s, 1H, Harom), 6.23 (bs, 1H, OH), 4.30 (t, *J* = 7.5 Hz, 1H, NCH_2_), 3.86 (s, 3H, OCH_3_), 3.13 (t, *J* = 7.5 Hz, 2H, CH_2_Ph), 2.38 (s, 3H, CH_3_); ^13^C NMR + DEPT (CDCl_3_, 100 MHz): δ 185.78 (C=O), 157.51 (Cquat), 156.58 (Cquat), 149.39 (Cquat), 143.78 (Cquat), 140.50 (Cquat), 135.93 (Cquat), 135.80 (Cquat), 131.97 (CH), 130.74 (CH), 129.08 (CH), 128.67 (CH), 125.31 (Cquat), 123.01 (CH), 120.84 (CH), 118.37 (CH), 114.61 (Cquat), 110.95 (Cquat), 110.34 (CH), 109.37 (CH), 103.43 (CH), 55.23 (CH_3_), 45.66 (CH_2_), 31.77 (CH_2_), 22.20 (CH_3_); HRMS calculated for C_25_H_21_NO_3_ [M+H]^+^ 384.1594, found 384.1593.

***9-Hydroxy-5-(2-(5-methoxy-1H-indol-3-yl)ethyl)indeno[1,2-b]indol-10(5H)-one (6c):*** dark red solid; yield 35%; mp 95 °C; IR (ν, cm^-1^): 3350 (OH and NH), 1662 (C=O); ^1^H NMR (CDCl_3_, 400 MHz): δ 7.83 (d, *J* = 7.7 Hz, 1H, Harom), 7.80 (bs, 1H, Harom), 7.69 (d, *J* = 7.2 Hz, 1H, Harom), 7.57 (d, *J* = 7.7 Hz, 1H, Harom), 7.50 (t, *J* = 7.3 Hz, 1H, Harom), 7.13 (d, *J* = 8.8 Hz, 1H, Harom), 7.04 (t, *J* = 8.1 Hz, 1H, Harom), 6.99 (d, *J* = 2.3 Hz, 1H, Harom), 6.82 (dd, *J* = 8.7, 2.4 Hz, 1H, Harom), 6.32 (d, *J* = 7.9 Hz, 1H, Harom), 6.22 (d, *J* = 8.0 Hz, 1H, Harom), 4.65 (s, 1H, OH), 4.04 (dd, *J* = 14.4, 4.4 Hz, 1H, Ha of NCH_2_), 4.02 (s, 3H, OCH_3_), 3.74-3.66 (m, 1H, Hb of NCH_2_), 3.04-2.96 (m, Ha of CH_2_Ph), 2.83 (dd, *J* = 15.3, 3.0 Hz, 1H, Hb of CH_2_Ph); ^13^C NMR + DEPT (CDCl_3_, 100 MHz): δ 206.08 (C=O), 157.14 (Cquat), 154.63 (Cquat), 154.41 (Cquat), 151.37 (Cquat), 137.28 (CH), 134.34 (Cquat), 133.24 (Cquat), 131.86 (Cquat), 131.15 (CH), 130.09 (CH), 127.21 (Cquat), 12.92 (CH), 124.92 (CH), 112.73 (CH), 112.08 (CH), 111.92 (Cquat), 108.91 (Cquat), 107.70 (CH), 100.78 (CH), 100.66 (CH), 71.41 (Cquat), 61.42 (CH), 56.11 (OCH_3_), 41.44 (NCH_2_), 19.57 (CH_2_); HRMS calculated for C_26_H_20_N_2_NaO_3_ [M+Na]^+^ 431.1366, found 431.1373.

***3,9-Dihydroxy-5-(3-methoxyphenethyl)indeno[1,2-b]indol-10(5H)-one (6d):*** red solid; yield 12%; mp 198 °C; IR (ν, cm^-1^): 3460 - 3300 (OH), 1649 (C=O); ^1^H NMR (DMSO-*d*_6_, 400 MHz): δ 10.21 (bs, 1H, OH-3), 9.31 (bs, 1H, OH-9), 7.13-7.10 (m, 2H, Harom), 6.98-6.93 (m, 2H, Harom), 6.74-6.70 (m, 3H, Harom), 6.67 (d, *J* = 2.0 Hz, 1H, Harom), 6.55 (dd, *J* = 6.3 Hz, 2.1 Hz, 1H, Harom), 6.47 (dd, *J* = 7.9 Hz, 2.0 Hz, 1H, Harom), 4.52 (t, *J* = 7.1 Hz, 2H, NCH_2_), 3.65 (s, 3H, OCH_3_), 3.05 (t, *J* = 7.1 Hz, 2H, CH_2_Ph); ^13^C NMR + DEPT (DMSO-*d*_6_, 100 MHz): δ 183.09 (C=O), 161.38 (Cquat), 159.31 (Cquat), 155.24 (Cquat), 150.86 (Cquat), 143.54 (Cquat), 139.12 (Cquat), 136.52 (Cquat), 130.55 (Cquat), 129.42 (CH), 124.06 (2CH), 121.16 (CH), 114.45 (CH), 114.27 (Cquat), 112.68 (Cquat), 112.65 (CH), 112.42 (CH), 108.49 (CH), 107.71 (CH), 102.94 (CH), 54.88 (OCH_3_), 46.25 (NCH_2_), 36.51 (CH_2_); HRMS calculated for C_24_H_19_NNaO_4_ [M+Na]^+^ 408.1206, found 408.1214.

***9-Hydroxy-2,3-dimethoxy-7-methyl-5-phenethylindeno[1,2-b]indol-10(5H)-one (6e):*** orange solid; yield 35%; mp 198 °C; IR (ν, cm^-1^): 3460 (OH), 1649 (C=O); ^1^H NMR (CDCl_3_, 400 MHz): δ 7.26-7.11 (m, 3H, H-3’, H-4’ and H-5’), 6.98 (d, *J* = 6.7 Hz, 2H, H-2’ and H-6’), 6.80 (s, 1H, OH), 6.46 (s, 1H, H-1), 6.42 (s, 1H, H-4), 6.17 (s, 1H, H-6), 6.02 (s, 1H, H-8), 4.14 (t, *J* = 6.7 Hz, 2H, NCH_2_), 3.81 (s, 3H, OCH_3_), 3.78 (s, 3H, OCH_3_), 3.06 (t, *J* = 6.7 Hz, 2H, CH_2_Ph), 2.35 (s, 3H, CH_3_); ^13^C NMR + DEPT (CDCl_3_, 100 MHz): δ 185.74 (C=O), 156.63 (Cquat), 150.76 (Cquat), 148.93 (Cquat), 148.62 (Cquat), 142.92 (Cquat), 137.58 (Cquat), 135.03 (Cquat), 132.94 (Cquat), 129.44 (Cquat), 128.95 (2CH), 128.86 (2CH), 127.33 (CH), 113.45 (Cquat), 111.29 (Cquat), 109.48 (CH), 108.10 (CH), 103.87 (CH), 103.43 (CH), 56.27 (OCH_3_), 56.20 (OCH_3_), 47.48 (NCH_2_), 36.15 (CH_2_), 22.17 (CH_3_); HRMS calculated for C_26_H_23_NNaO_4_ [M+Na]^+^ 436.1519, found 436.1505.

**NMR and HR-MS spectra of tested compounds**

***5-(2-Methoxyphenethyl)-7-methyl-5,6,7,8-tetrahydroindeno[1,2-b]indole-9,10-dione (5a)***

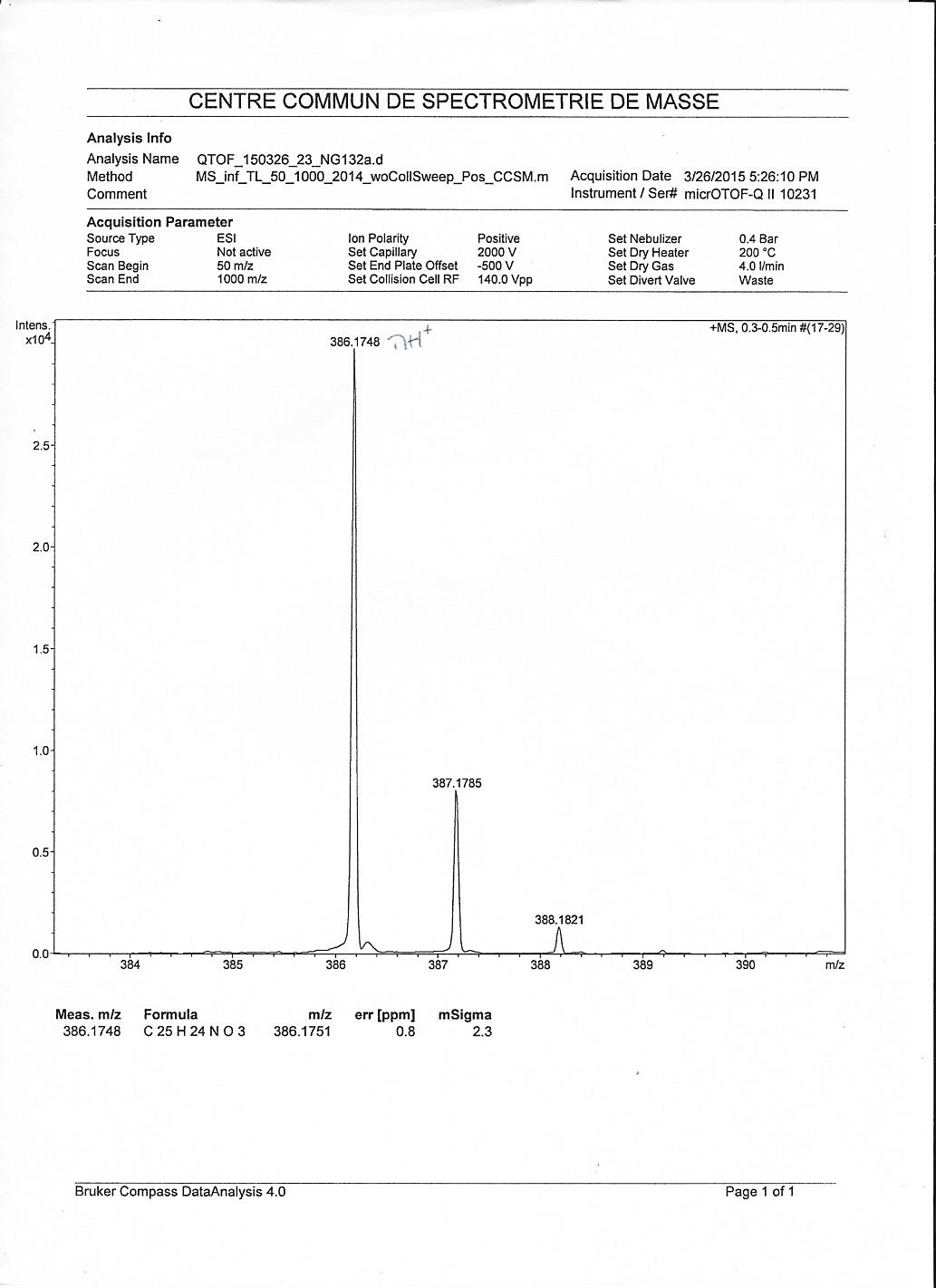


***8-Benzyl-5-phenethyl-5,6,7,8-hexahydroindeno[1,2-b]indole-9,10-dione (5b)***

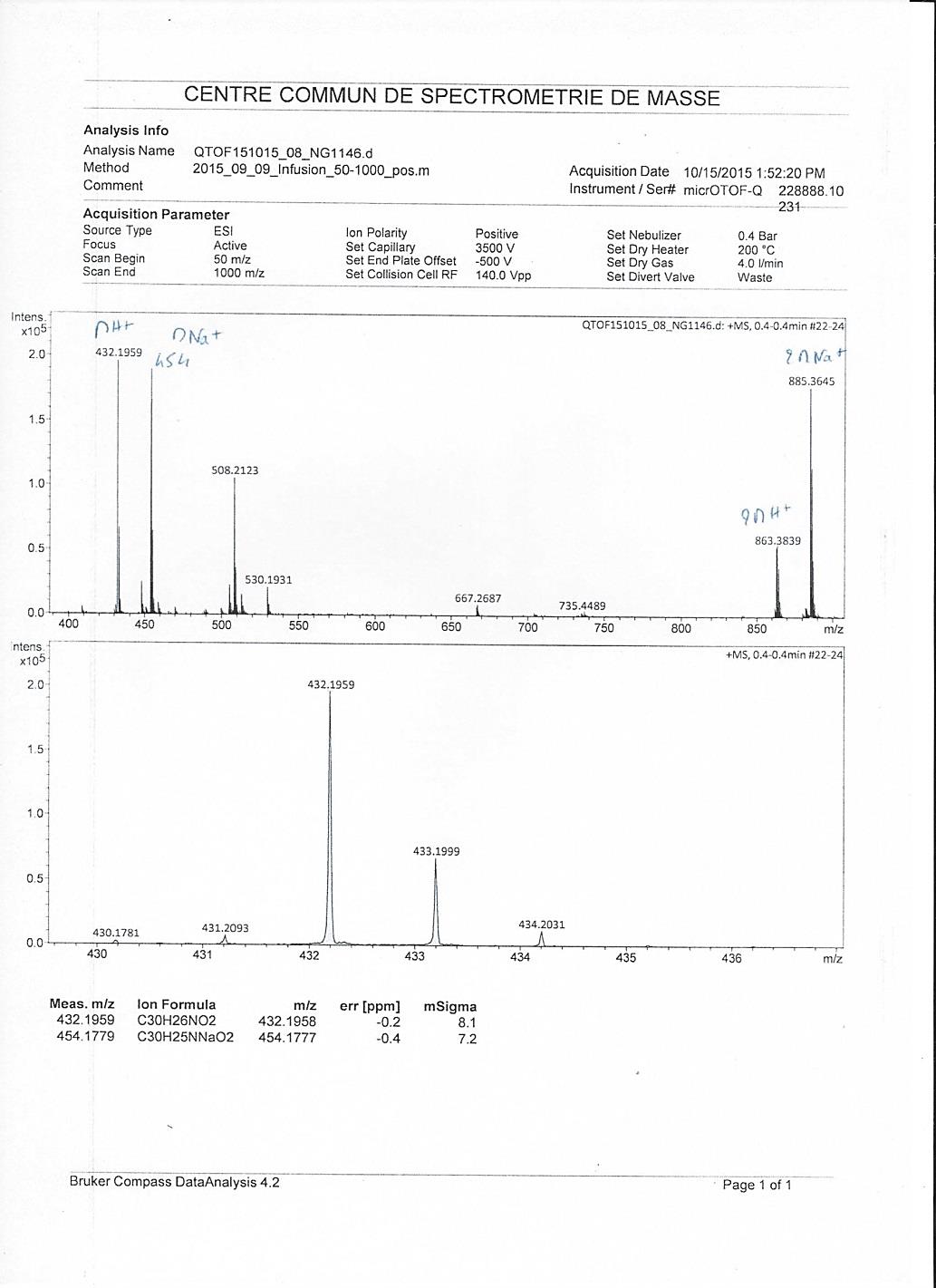


***5-(2-(5-Methoxy-1H-indol-3-yl)ethyl)-5,6,7,8-tetrahydroindeno[1,2-b]indole-9,10-dione (5c)***

**
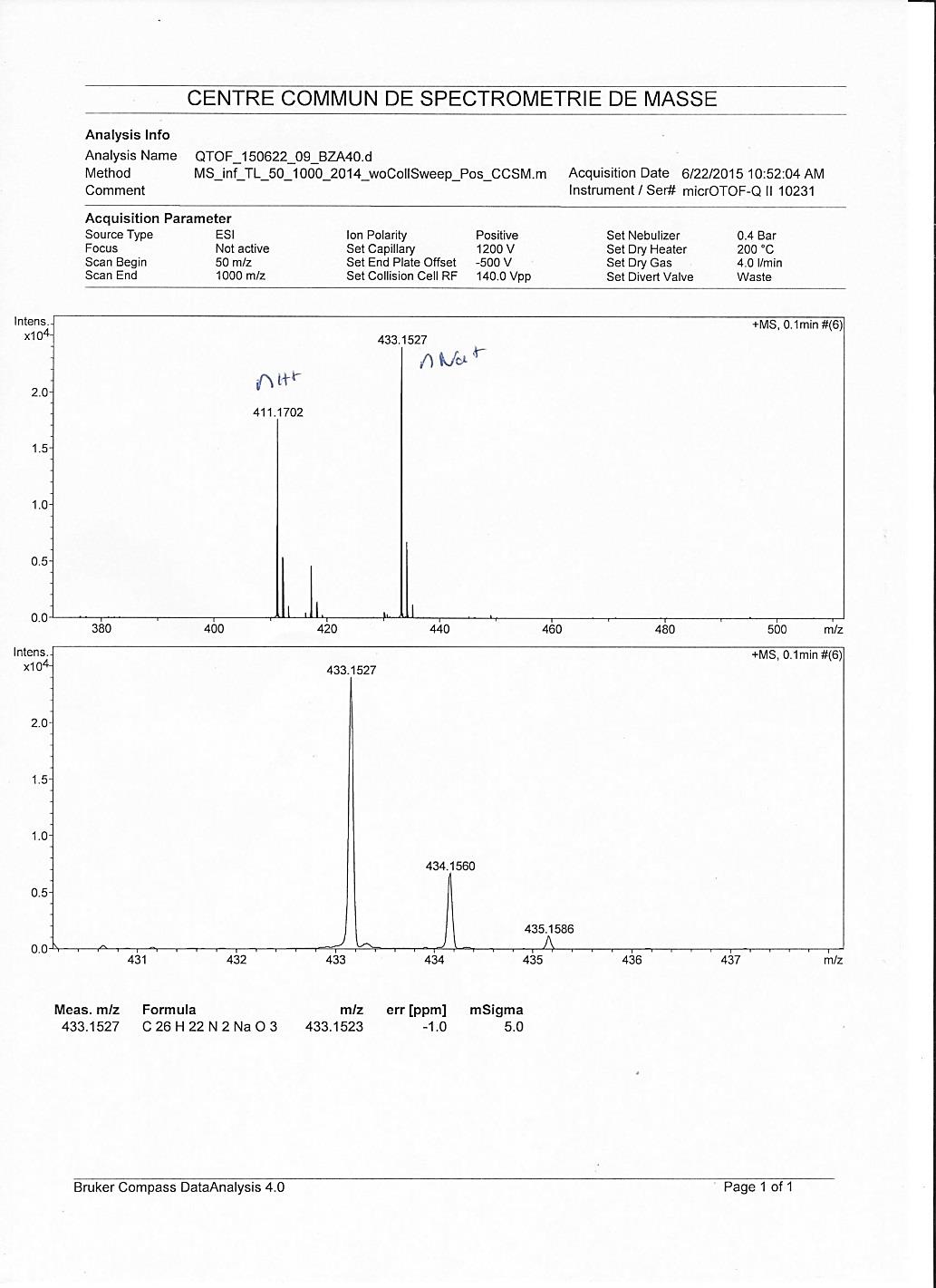
**

***3-Hydroxy-5-(3-methoxyphenethyl)-5,6,7,8-tetrahydroindeno[1,2-b]indole-9,10-dione (5d)***

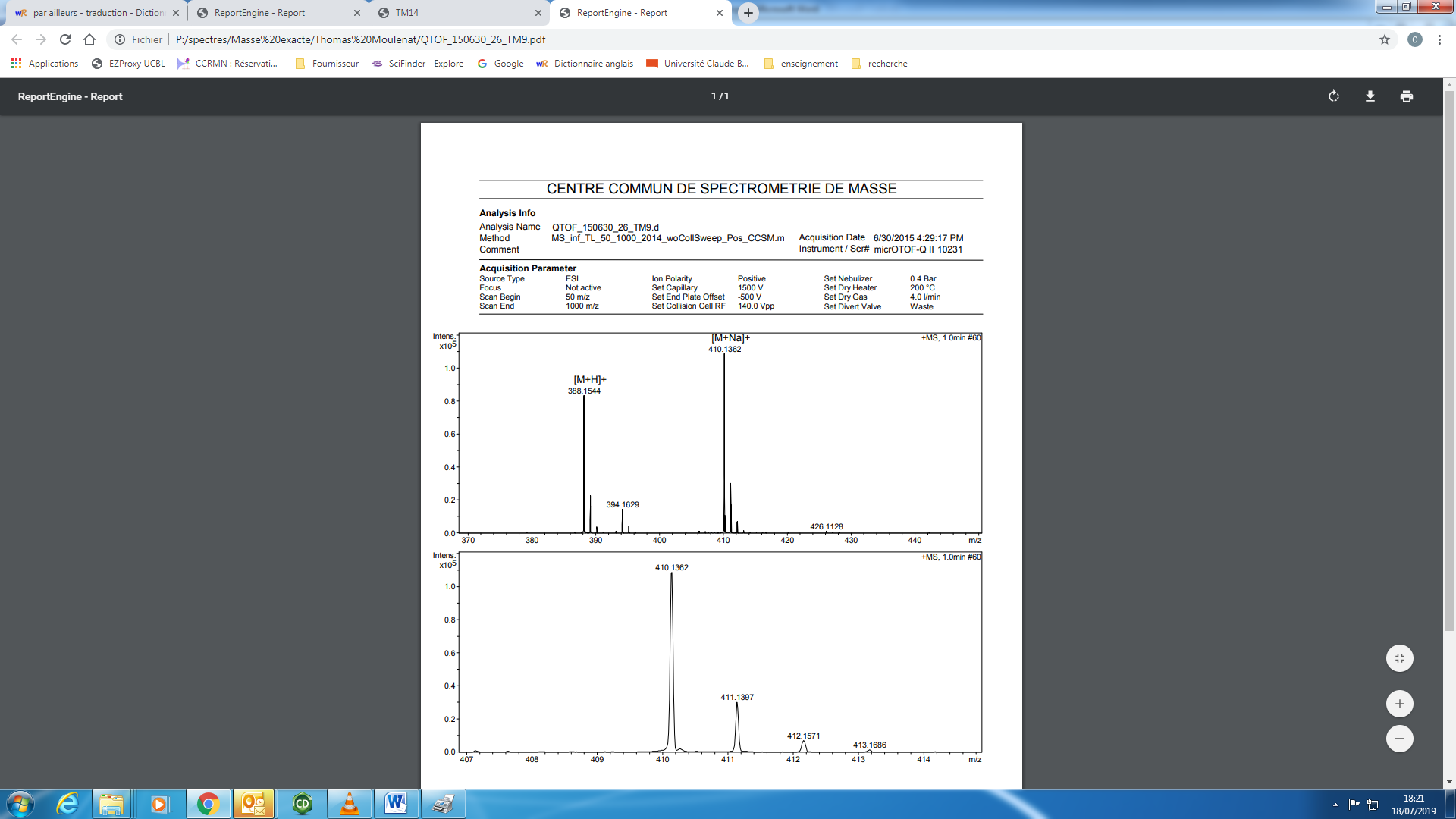


***2,3-Dimethoxy-7-methyl-5-phenethyl-5,6,7,8-tetrahydroindeno[1,2-b]indole-9,10-dione (5e)***

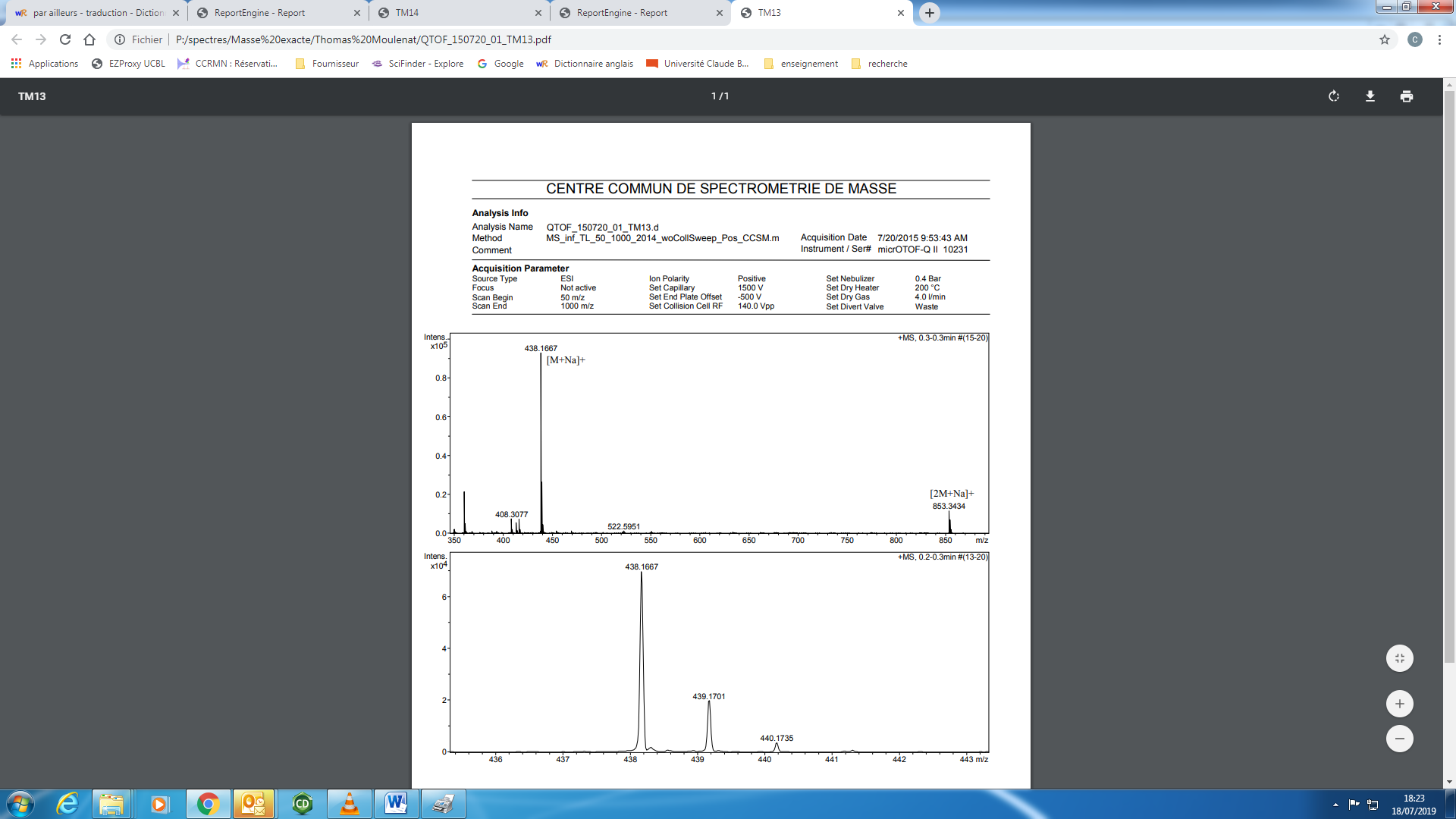


***1-Hydroxy-7-Methyl-5-phenethyl-5,6,7,8-tetrahydroindeno[1,2-b]indole-9,10-dione (5f)***

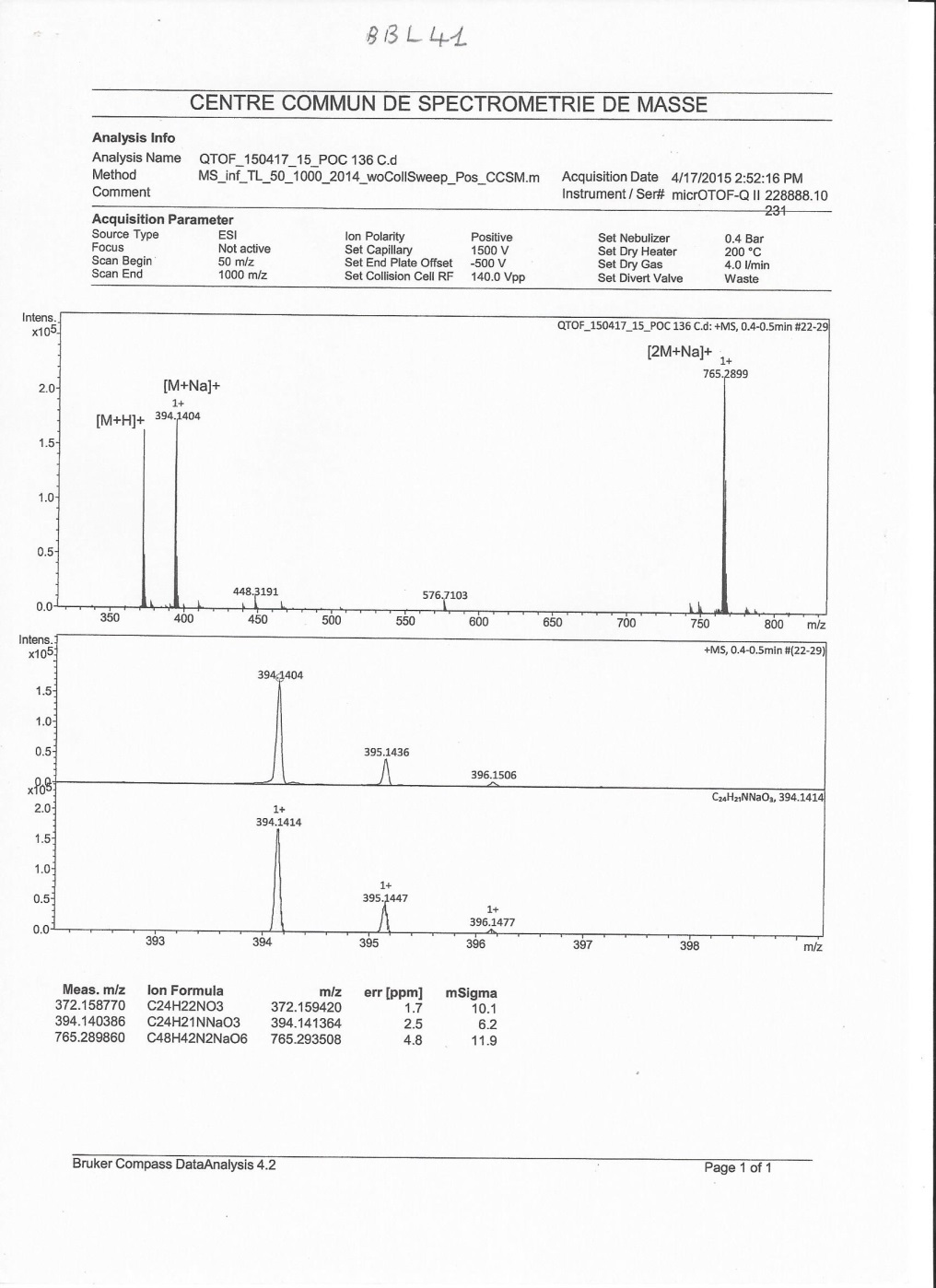


***4-Hydroxy-7-methyl-5-phenethyl-5,6,7,8-tetrahydroindeno[1,2-b]indole-9,10-dione (5g)***

**
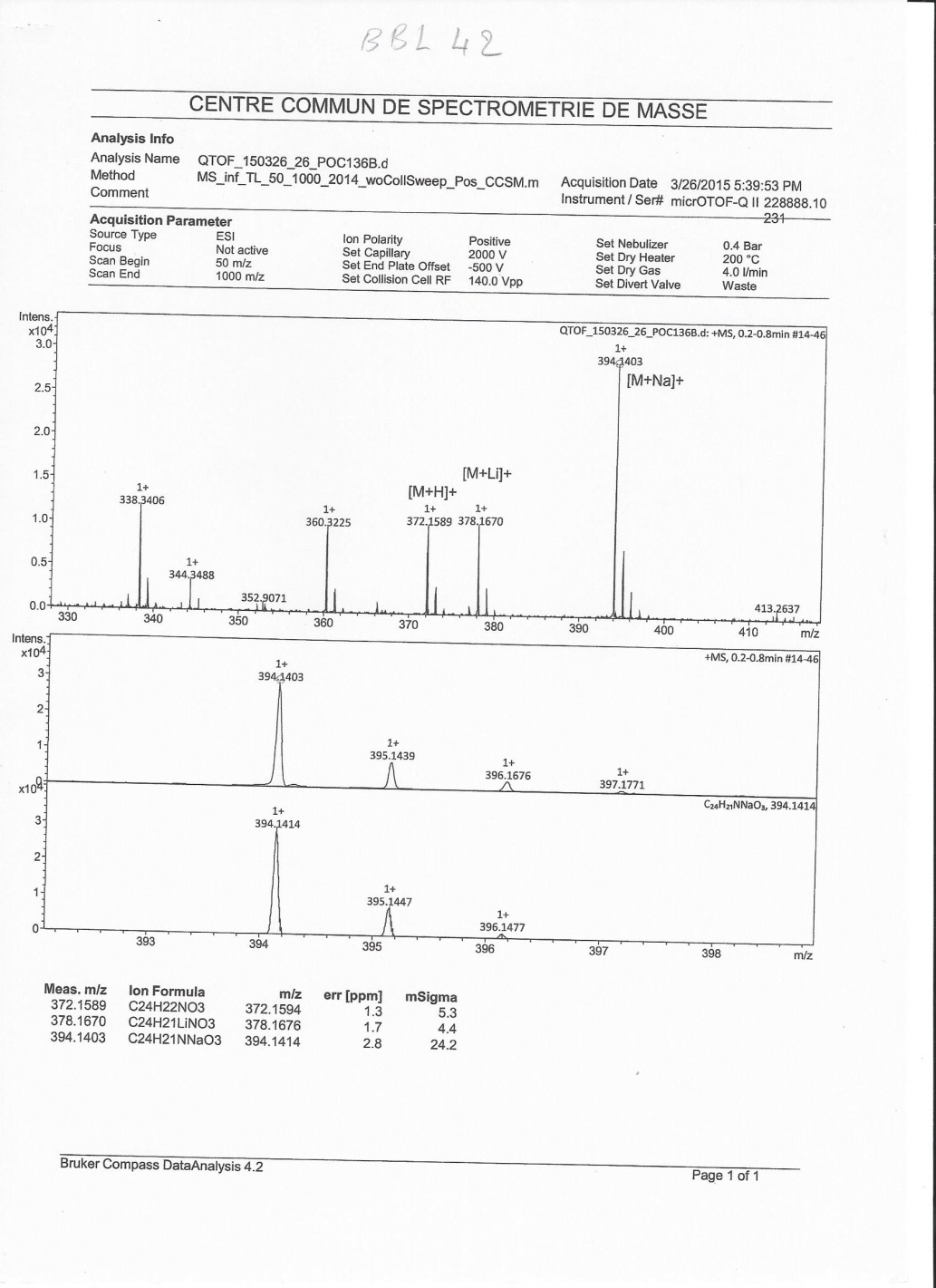
**

***1-Hydroxy-5-(2-methoxyphenethyl)-5,6,7,8-tetrahydroindeno[1,2-b]indole-9,10-dione (5h)***

**
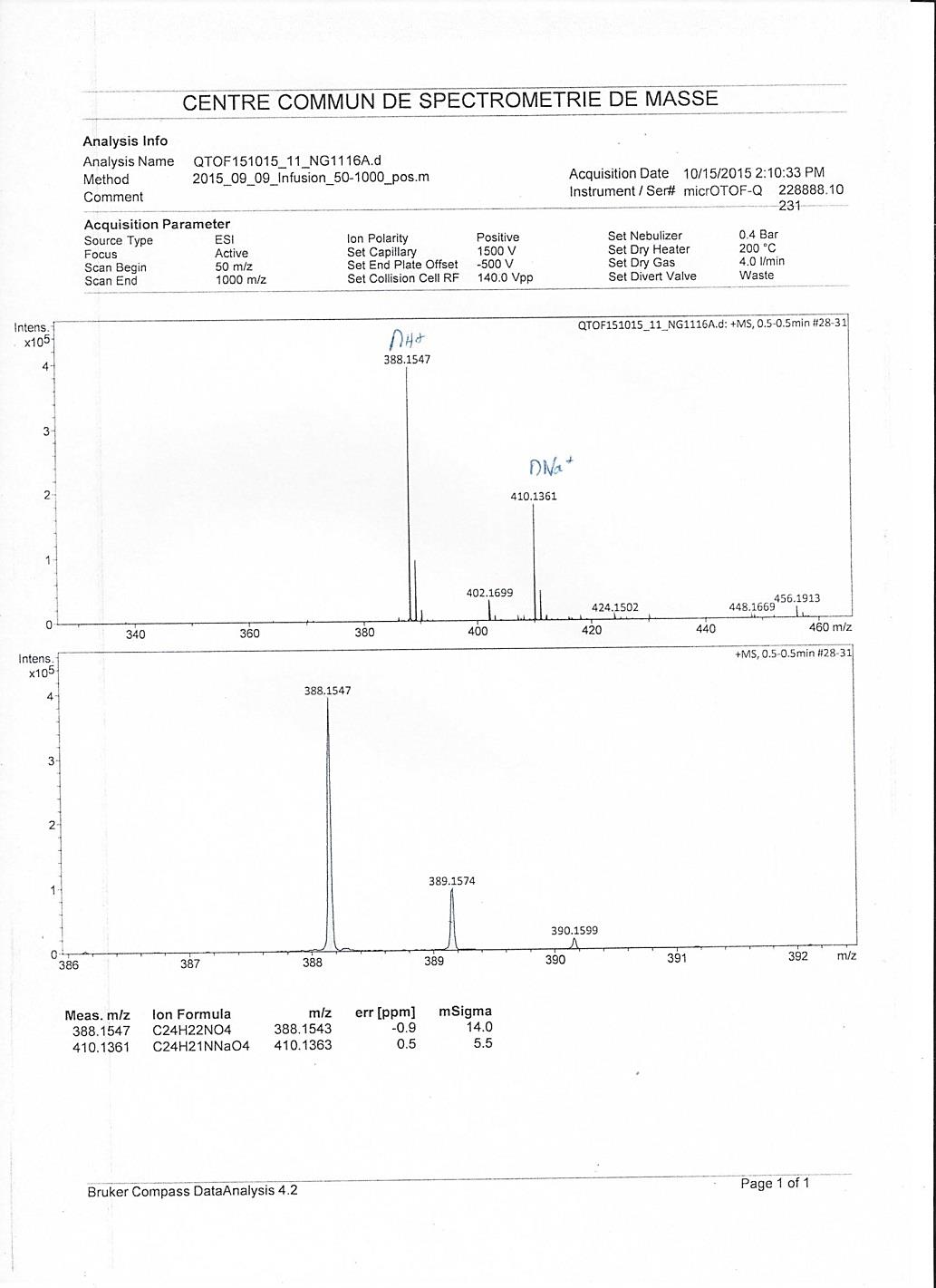
**

***1-Hydroxy-7-methyl-5-(2-methoxyphenethyl)-5,6,7,8-tetrahydroindeno[1,2-b]indole-9,10-dione (5i)***

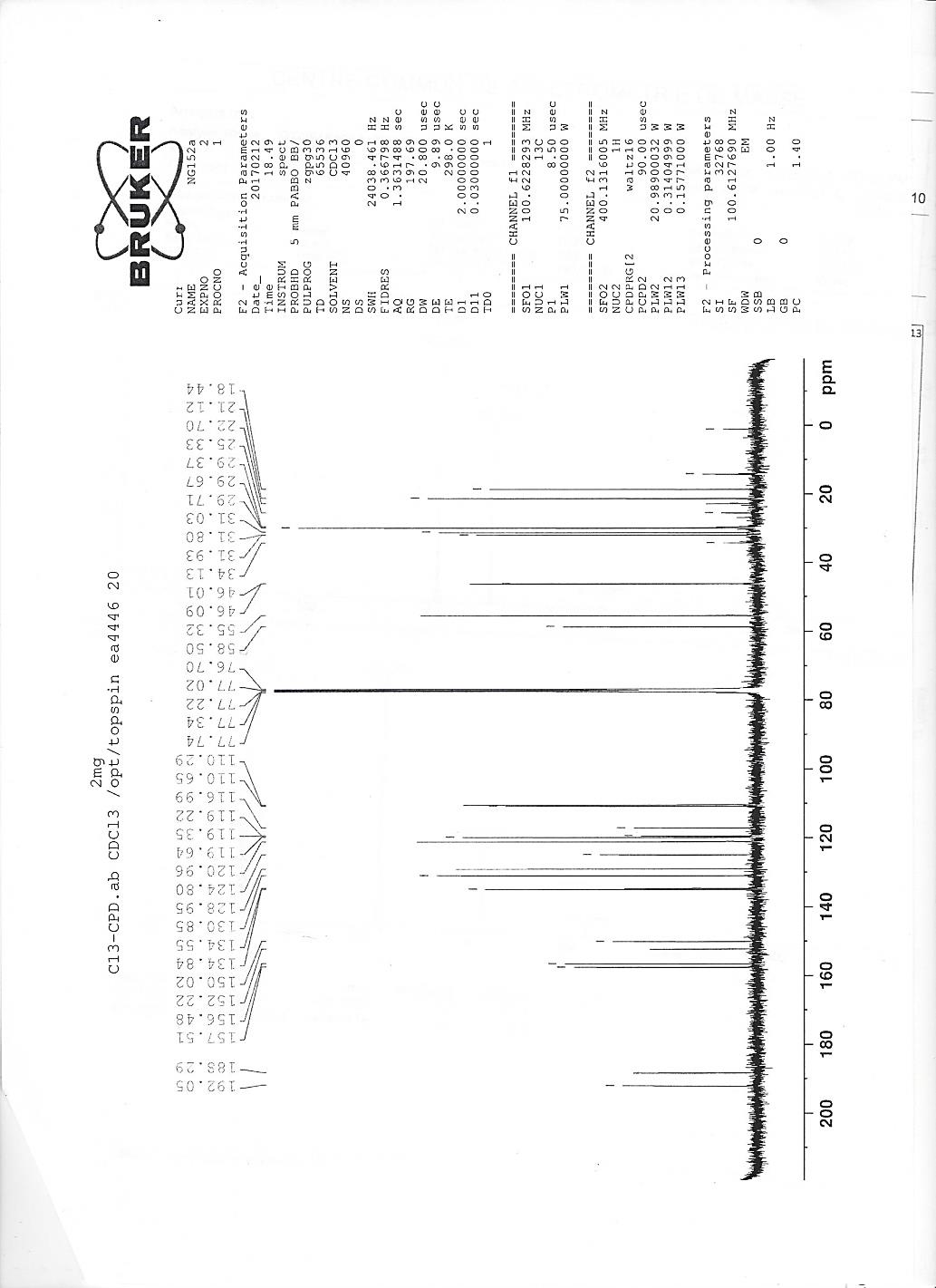


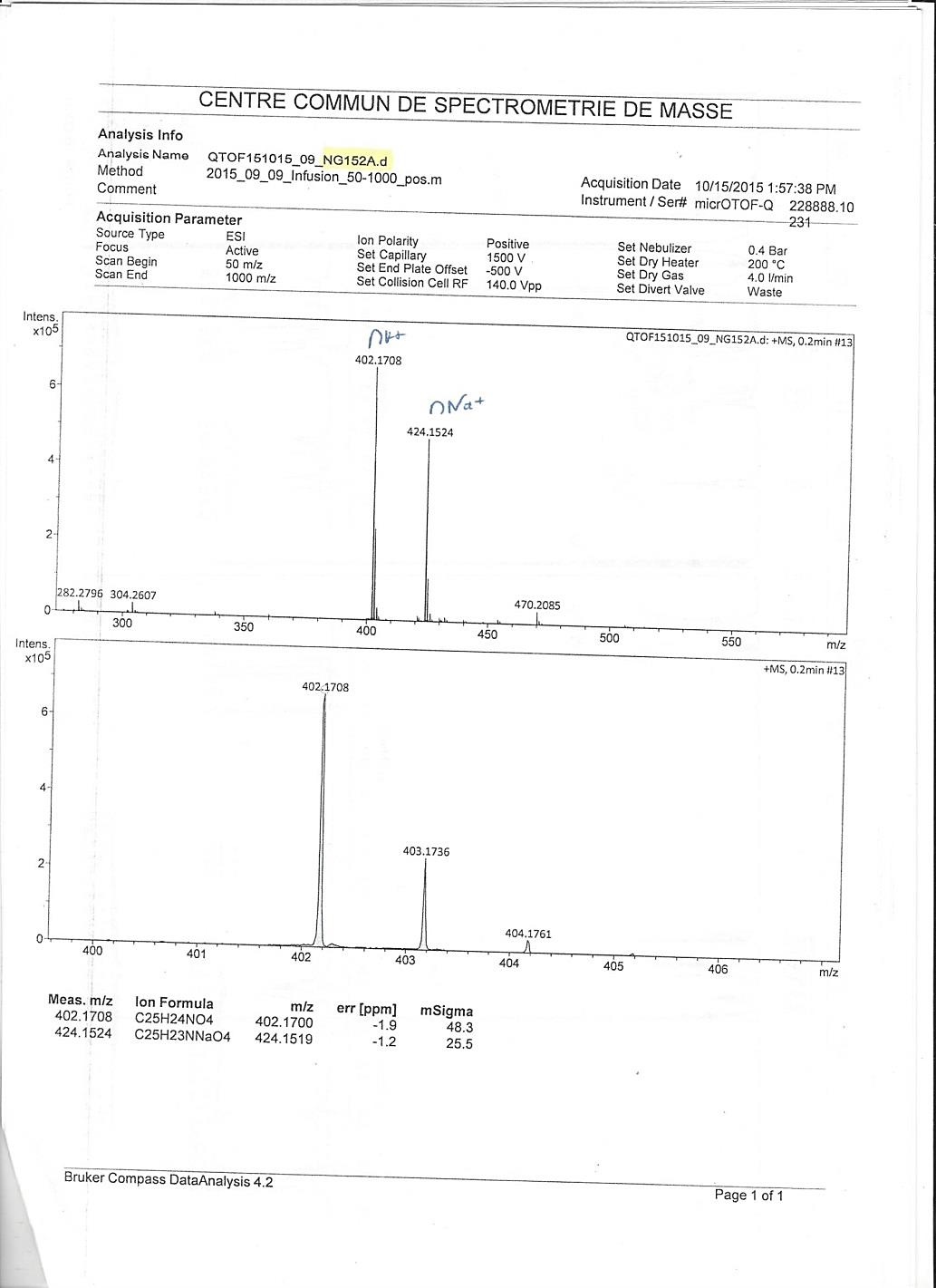


***5-(2-Methoxyphenethyl)-1-(3-methylbut-2-enyloxy)-5,6,7,8-tetrahydroindeno[1,2-b]indole-9,10-dione (5j)***

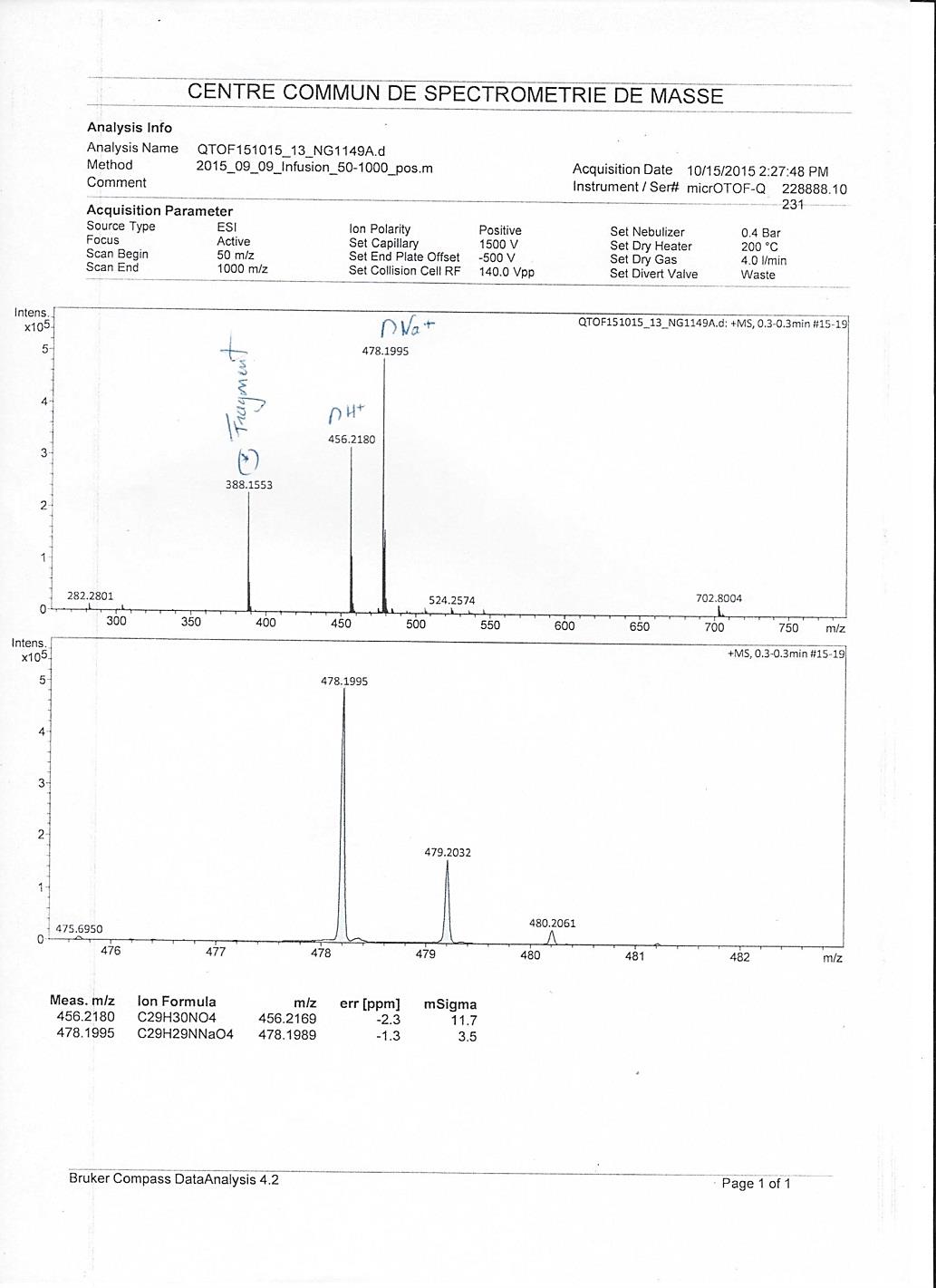


***5-(2-Methoxyphenethyl)-4-(3-methylbut-2-enyloxy)-5,6,7,8-hexahydroindeno[1,2-b]indole-9,10-dione (5k)***

**

**
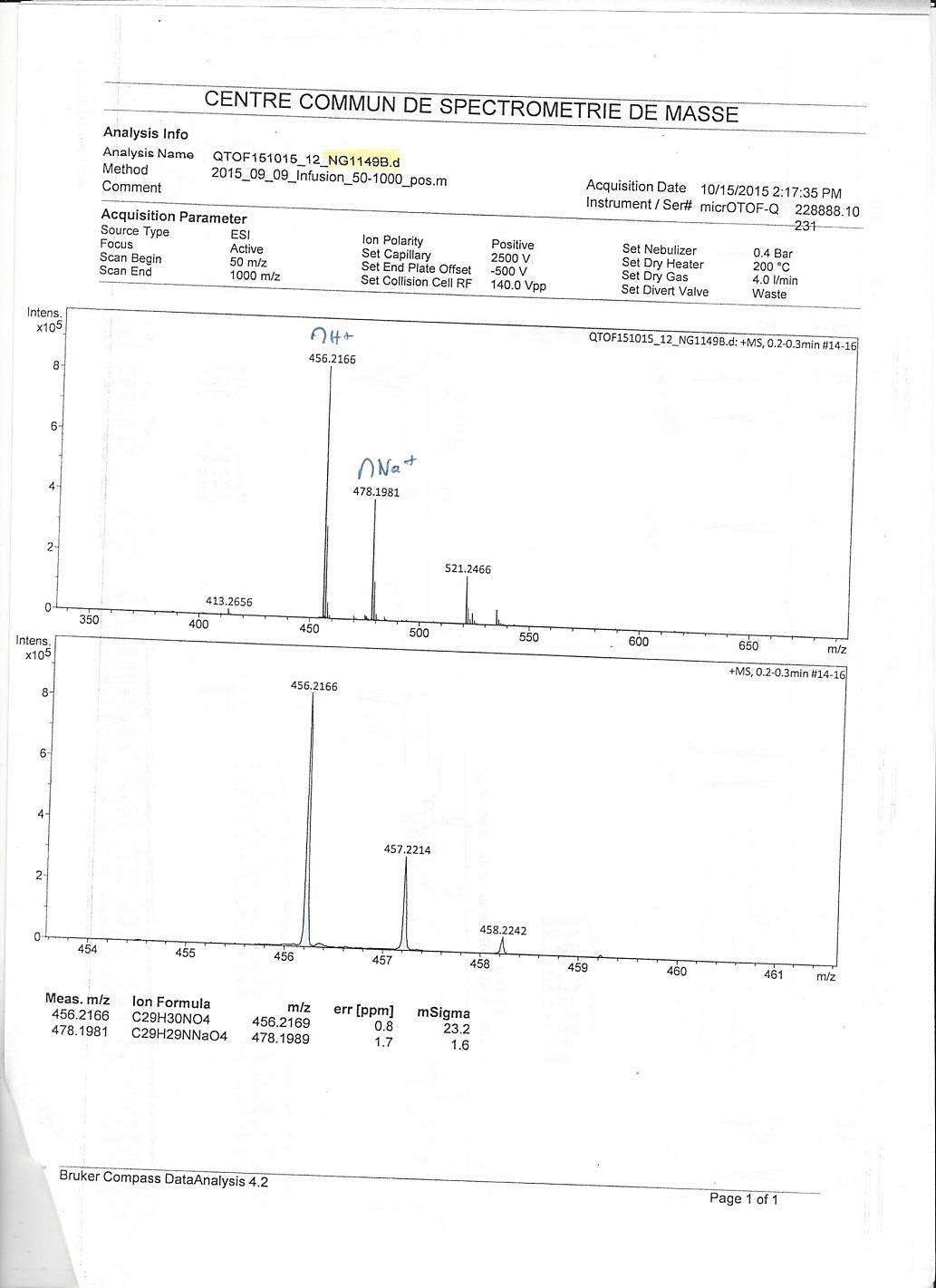
**

**5*-(2-Methoxyphenethyl)-7-methyl-1-((3-methylbut-2-enyl)oxy)-5,6,7,8-tetrahydroindeno[1,2-b]indole-9,10-dione (5l)***

**
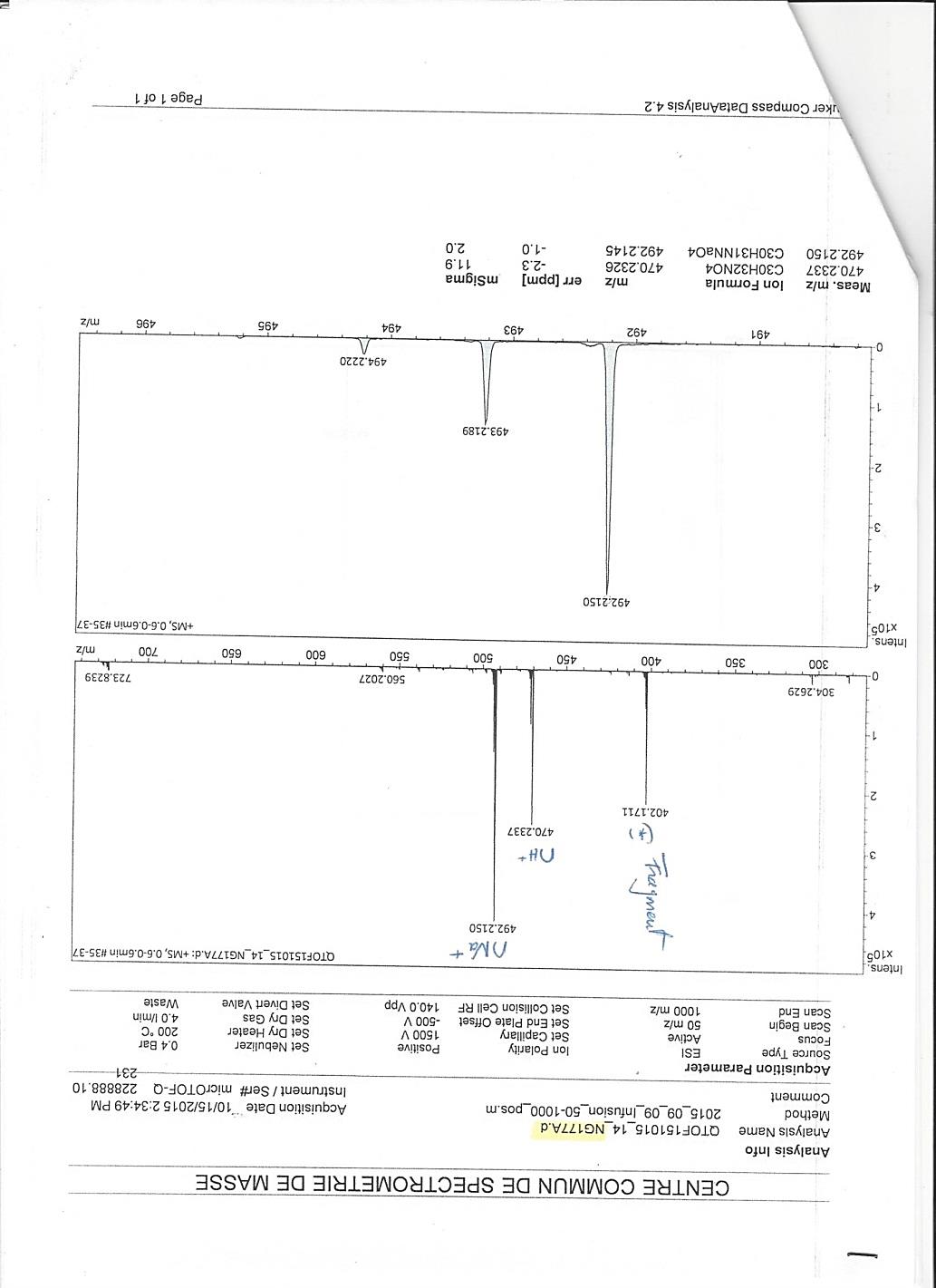
**

***5-(2-Methoxyphenethyl)-7-methyl-4-((3-methylbut-2-enyl)oxy)-5,6,7,8-hexahydroindeno[1,2-b]indole-9,10-dione (5m)***

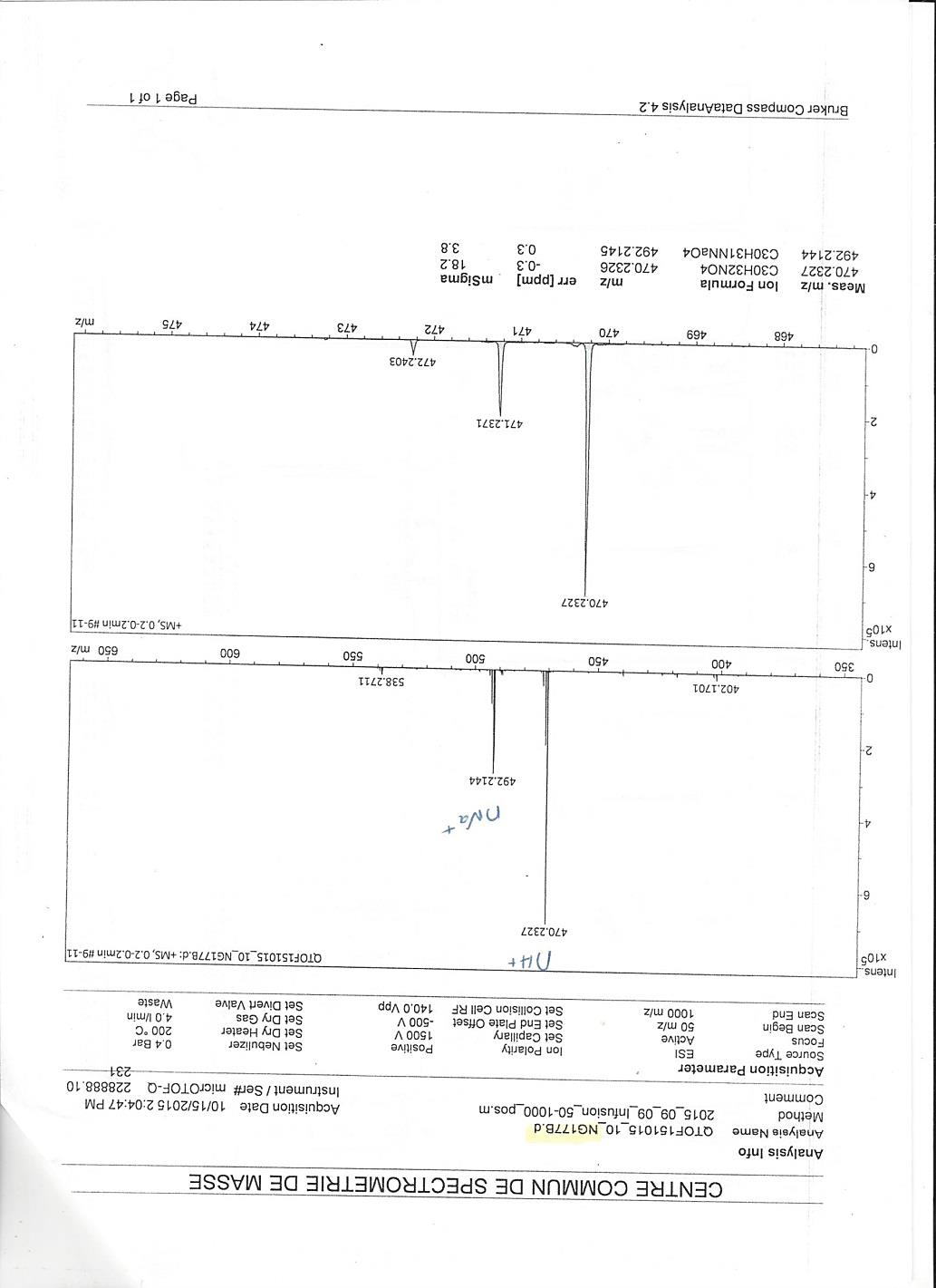


***7-Methyl-4-(3-methylbut-2-enyloxy)-5-phenethyl)-5,6,7,8-hexahydroindeno[1,2-b]indole-9,10-dione (5n)***

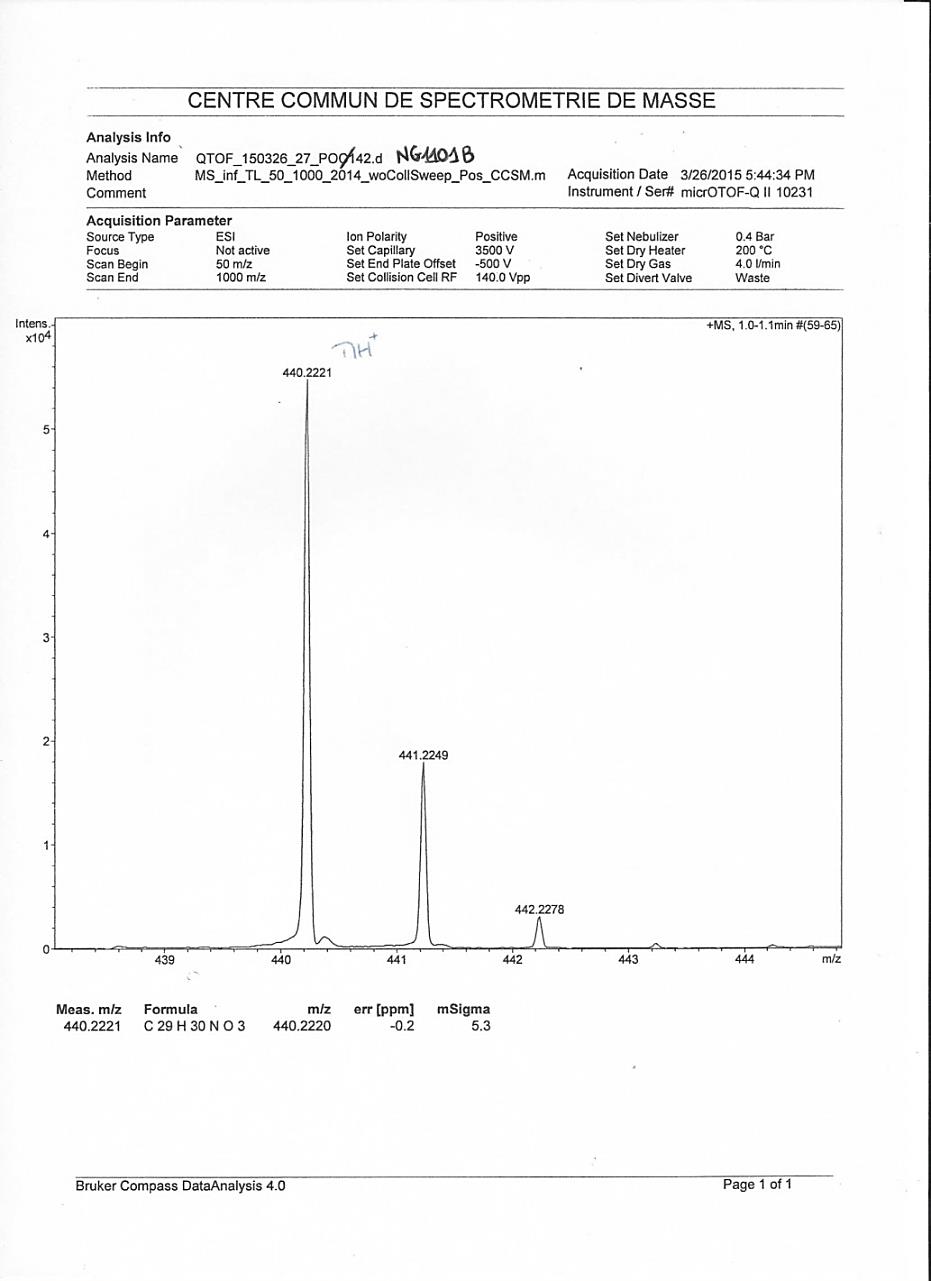


***9-Hydroxy-7-methyl-5-(2-methoxyphenethyl)-5H-indeno[1,2-b]indol-10(5H)-one (6a)***

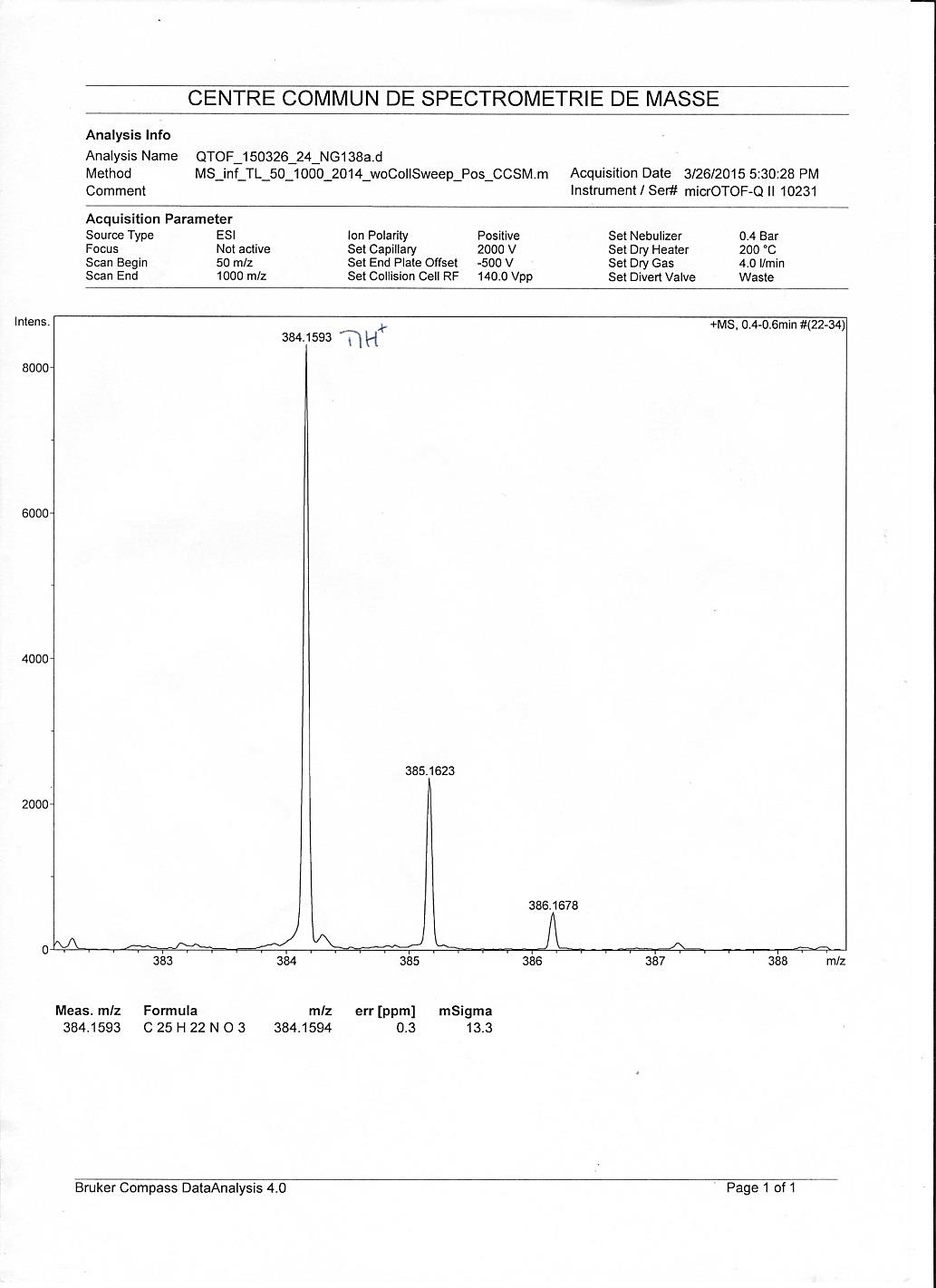


***9-Hydroxy-5-(2-(5-methoxy-1H-indol-3-yl)ethyl)indeno[1,2-b]indol-10(5H)-one (6c)***

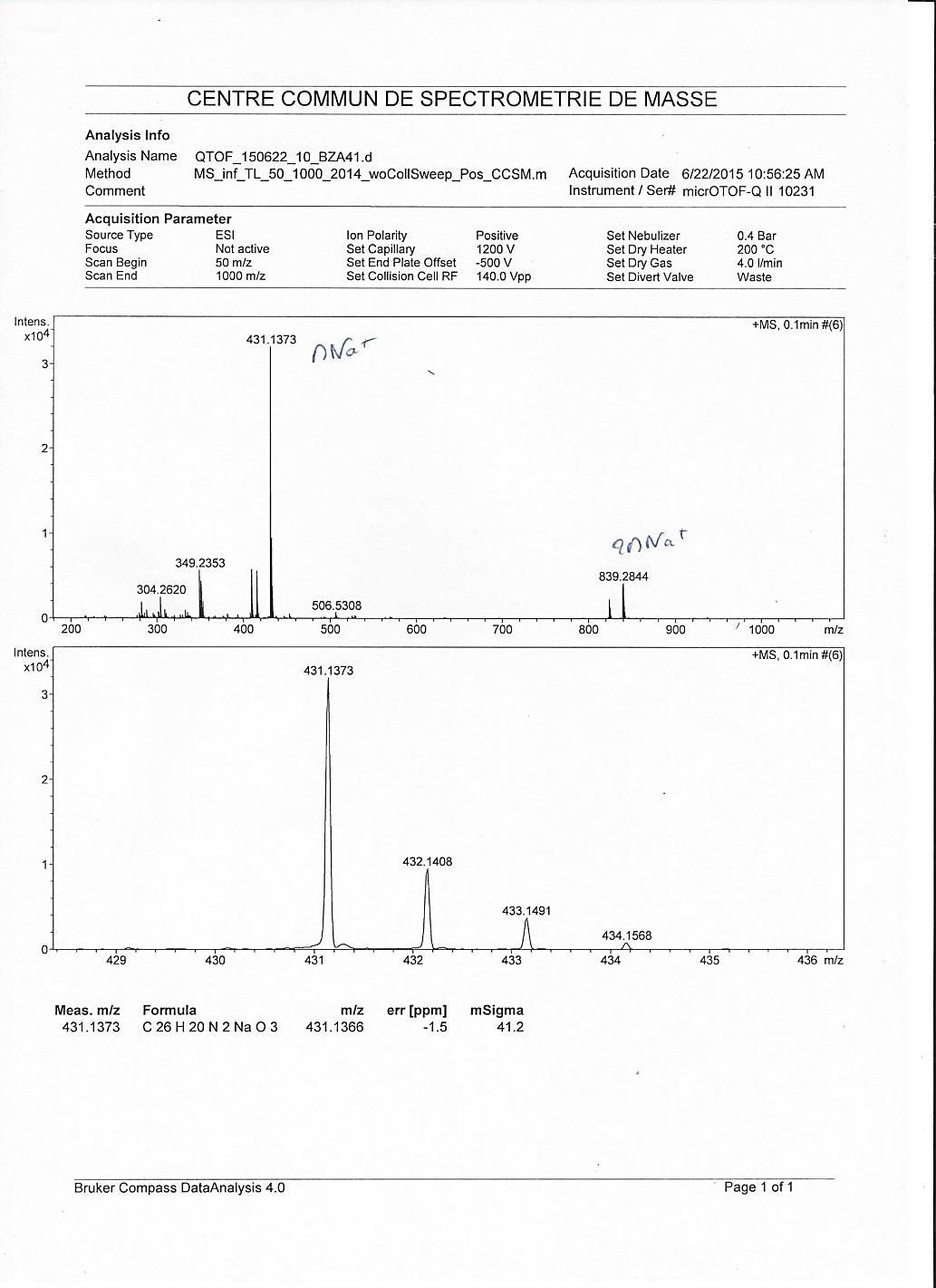


***3,9-Dihydroxy-5-(3-methoxyphenethyl)indeno[1,2-b]indol-10(5H)-one (6d)***

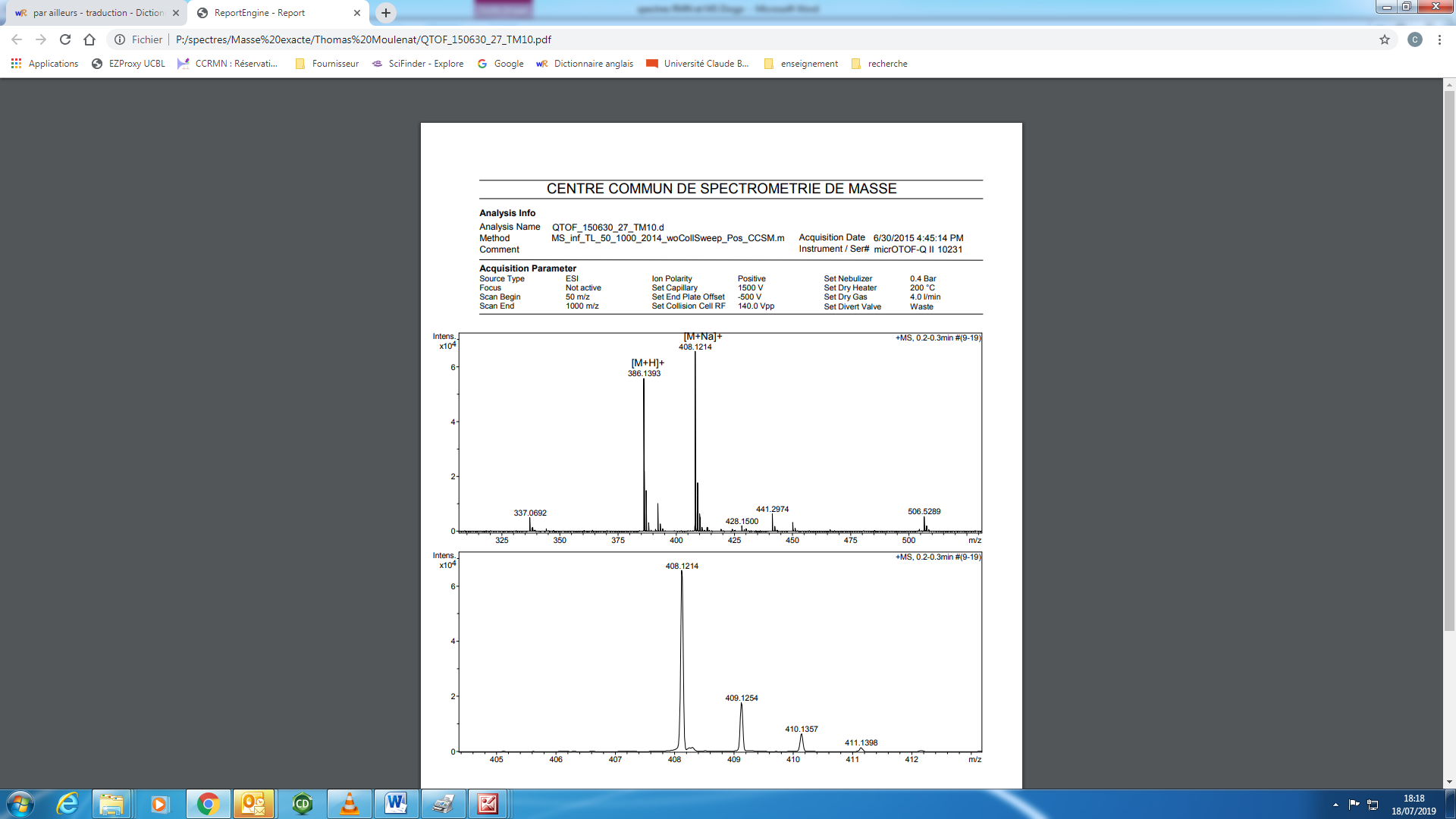


***9-Hydroxy-2,3-dimethoxy-7-methyl-5-phenethylindeno[1,2-b]indol-10(5H)-one (6e)***

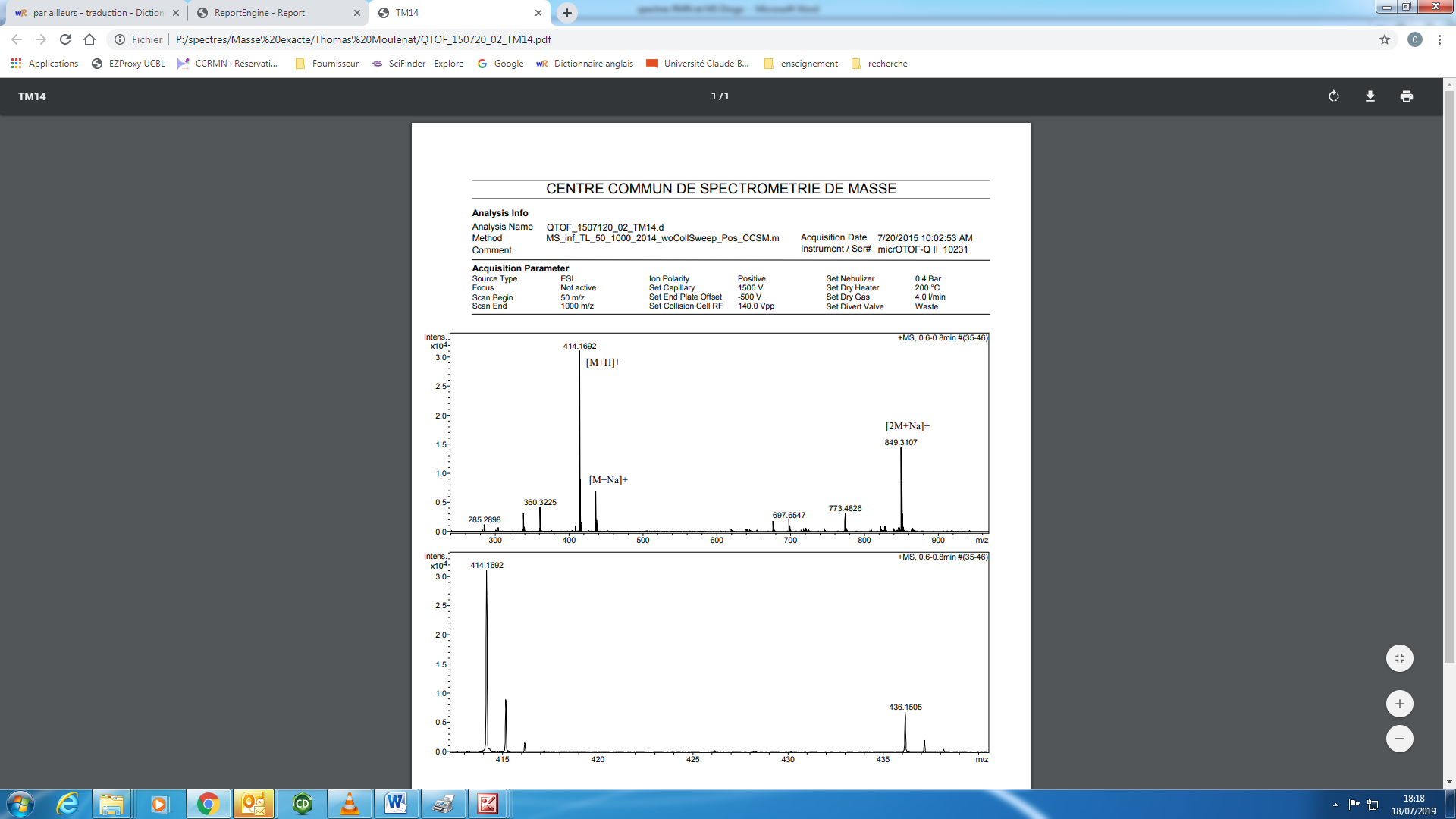


**Biology experiments**

**Figure S6.** Inhibition of mitoxantrone efflux (%) in HEK293-ABCG2 transfected cells at 1 and 10 µM by various compounds. The percent inhibition of ABCG2 transport was determined by flow cytometry using the reference inhibitor Ko143 as a control, which produces 100% inhibition. The data are the mean ± SD of three independent experiments performed in triplicate.


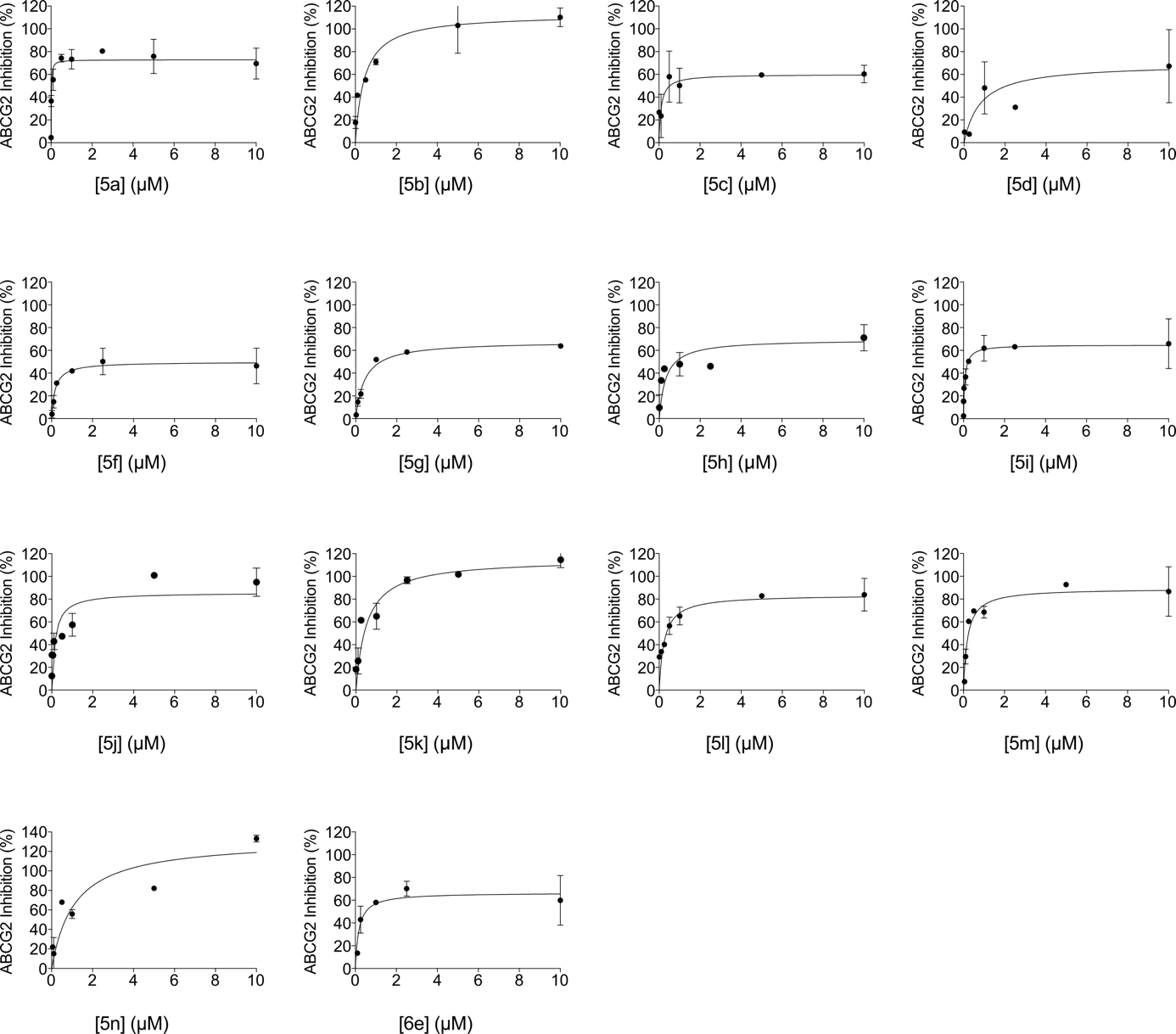


**Figure S7.** Inhibition potency of 14 indeno[1,2-*b*] indole derivatives. Representative IC_50_ curves.


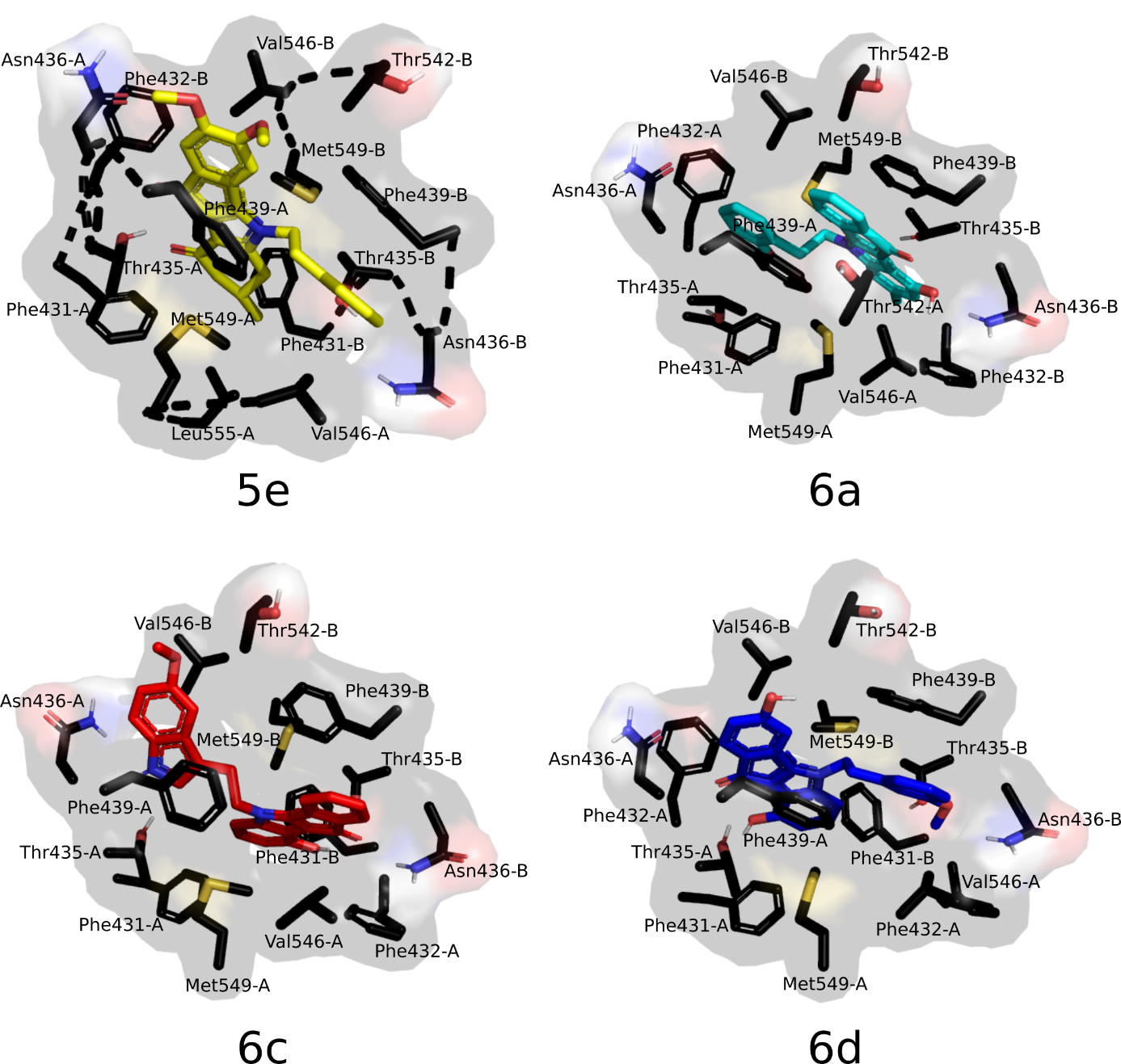


**Figure S8.** Docking analysis in human ABCG2 (PDB 6FFC) [S4].

**Figure S9.** Structural modifications that increase the ABCG2 inhibition potency.

| **Cells** | **Compound** | **Concentration (µM)** | **IG_50_ (SN-38 nM ± SD)^a^** | **FR^b^** |
| --- | --- | --- | --- | --- |
| HEK293 WT | **6a** | - | 10.03 ± 5.78 | 1.00 |
| HEK293-*ABCG2* |  | - | 103.88 ± 3.82 | 10.36 |
|  |  | 0.21 | 8.18 ± 2.35 | 0.82 |
|  |  | 5 | 4.69 ± 0.72 | 0.47 |
| HEK293 WT | **5e** | - | 7.58 ± 2.63 | 1.00 |
| HEK293-*ABCG2* |  | - | 159.13 ± 31.61 | 20.99 |
|  |  | 0.15 | 72.09 ± 3.42 | 9.51 |
|  |  | 5 | 23.38 ± 19.99 | 3.08 |
| HEK293 WT | **6c** | - | 8.24 ± 2.34 | 1.00 |
| HEK293-*ABCG2* |  | - | 87.59 ± 24.04 | 10.62 |
|  |  | 0.48 | 9.34 ± 404 | 1.13 |
|  |  | 5 | 3.06 ± 0.35 | 0.37 |
| HEK293 WT | **6d** | - | 7.53 ± 2.71 | 1.00 |
| HEK293-*ABCG2* |  | - | 502.02 ± 32.56 | 66.67 |
|  |  | 0.19 | 93.98 ± 43.33 | 12.48 |
|  |  | 5 | 4.80 ± 1.75 | 0.64 |

**Table S1.** Indeno[1,2-*b*]indole derivatives sensitize ABCG2 expressing cells to SN-38. Cell viability of HEK293-*ABCG2* and HEK293 control cells upon 72 h treatment with SN-38 at increasing concentrations, and HEK293-*ABCG2* cells upon co-treatment with SN-38 and inhibitors at either IC_50_ values or 5 µM. ^a^The cytotoxicity of SN-38 was expressed as IG_50_ values (concentrations giving a half-maximal cell viability). ^b^The fold reversal (FR) was calculated as the ratio between IG_50_ values of HEK293-*ABCG2* cells treated or not with inhibitors and IG_50_ values of HEK293 cells treated only with SN-38.

| **Compound** | **EC_50_ (nM ± SD) values of stimulation on the ATPase activity of ABCG2** |
| --- | --- |
| **5e** | 170 ± 11.00 |
| **6a** | 2.9 ± 0.45 |
| **6c** | 4.4 ± 0.71 |
| **6d** | 3.2 ± 0.44 |

**Table S2.** Effect of indeno[1,2-*b*]indole derivatives on the ATPase activity of ABCG2.The vanadate-sensitive ATPase activity of ABCG2 was measured using the total membranes of High-Five insect cells expressing this transporter as described previously [S5]. The EC_50_ (concentration required to achieve 50% stimulation) is given as the concentration ± SD (nM) of three independent experiments in duplicate.

| ABCG2 | | Number of poses | | | | | | | |
| --- | --- | --- | --- | --- | --- | --- | --- | --- | --- |
| **E3S** | | **5e** | | **6a** | | **6c** | | **6d** | |
| Chain A | Chain B | Chain A | Chain B | Chain A | Chain B | Chain A | Chain B | Chain A | Chain B |
| Thr435 | Thr435 | 7 | 7 | 5 | 8 | 8 | 7 | 9 | 8 |
| Asn436 |  | 4 | 4 | 3 | 5 | 7 | 5 | 8 | 8 |
| Phe439 | Phe439 | 9 | 9 | 9 | 9 | 9 | 9 | 9 | 9 |
|  | Thr542 | 9 | 9 | 5 | 8 | 7 | 7 | 4 | 8 |
|  | Ile543 | 0 | 0 | 0 | 0 | 0 | 0 | 0 | 0 |
| Val546 | Val546 | 9 | 8 | 6 | 8 | 7 | 9 | 8 | 9 |
| Met549 | Met549 | 9 | 8 | 9 | 9 | 9 | 9 | 8 | 8 |

**Table S3.** Comparison of residues that interact with estrone 3-sulfate and the indeno[1,2-*b*]indole derivatives in human ABCG2 (PDB 6HCO). The model of the transporter was derived from the Cryo-EM determined structure of human ABCG2 with bound to estrone 3-sulfate [S6].

**References**

S1 [Burla](http://scripts.iucr.org/cgi-bin/citedin?search_on=name&author_name=Burla,%20M.C.), M. C. *et al.* [*SIR2004*: an improved tool for crystal structure determination and refinement](http://journals.iucr.org/j/issues/2005/02/00/zm5026/index.html). [*J. Appl. Cryst.*](http://journals.iucr.org/j) **38**, 381–388, <https://doi.org/10.1107/S002188980403225X> (2005).

S2 [Sheldrick](http://scripts.iucr.org/cgi-bin/citedin?search_on=name&author_name=Sheldrick,%20G.M.), G. M. A short history of *SHELX*. [*Acta Cryst.*](http://journals.iucr.org/a) [A**64**](http://journals.iucr.org/a/contents/backissues.html), 112–122, <https://doi.org/10.1107/S0108767307043930> (2008).

S3 [Farrugia](https://scripts.iucr.org/cgi-bin/citedin?search_on=name&author_name=Farrugia%2C%20L%2EJ%2E), L. J. *WinGX* and *ORTEP for Windows*: an update. *J. Appl. Cryst.* [**45**](https://journals.iucr.org/j/services/archive.html), [849–854](https://journals.iucr.org/j/issues/2012/04/00/he5558/index.html), <https://doi.org/10.1107/S0021889812029111> (2012).

S4 Jackson, S. M. *et al.* Structural basis of small-molecule inhibition of human multidrug transporter ABCG2. *Nat. Struct. Mol. Biol.* **25**, 333–340, https://doi.org/ 10.1038/s41594-018-0049-1 (2018).

S5 Wu, C. P. *et al.* Evidence for dual mode of action of a thiosemicarbazone, NSC73306: A potent substrate of the multidrug resistance-linked ABCG2 transporter. *Mol. Cancer Ther.* **6**, 3287–3296, https://doi.org/10.1158/1535-7163.MCT-07-2005 (2007).

S6 Manolaridis, I. *et al.* Cryo-EM structures of a human ABCG2 mutant trapped in ATP-bound and substrate-bound states. *Nature* **563**, 426–432, https://doi.org/10.1038/s41586-018-0680-3 (2018).
